# Effect of Aspirin vs Enoxaparin on 90-Day Mortality in Patients Undergoing Hip or Knee Arthroplasty: A Secondary Analysis of the CRISTAL Cluster Randomized Trial

**DOI:** 10.1001/jamanetworkopen.2023.17838

**Published:** 2023-06-09

**Authors:** The CRISTAL Study Group, Verinder S. Sidhu, Thu-Lan Kelly, Nicole Pratt, Stephen E. Graves, Rachelle Buchbinder, Sam Adie, Kara Cashman, Ilana N. Ackerman, Durga Bastiras, Roger Brighton, Alexander W. R. Burns, Beng Hock Chong, Ornella Clavisi, Maggie Cripps, Mark Dekkers, Richard de Steiger, Michael Dixon, Andrew Ellis, Elizabeth C. Griffith, David Hale, Amber Hansen, Anthony Harris, Raphael Hau, Mark Horsley, Dugal James, Omar Khorshid, Leonard Kuo, Peter L. Lewis, David Lieu, Michelle Lorimer, Samuel J. MacDessi, Peter McCombe, Catherine McDougall, Jonathan Mulford, Justine Maree Naylor, Richard S. Page, John Radovanovic, Michael Solomon, Rami Sorial, Peter Summersell, Phong Tran, William L. Walter, Steve Webb, Chris Wilson, David Wysocki, Ian A. Harris

**Affiliations:** 1 School of Clinical Medicine, South Western Sydney Clinical School, Faculty of Medicine and Health, UNSW Sydney, Sydney, New South Wales, Australia; 2 Whitlam Orthopaedic Research Centre, Ingham Institute for Applied Medical Research, Liverpool, New South Wales, Australia; 3 Clinical and Health Sciences, Quality Use of Medicines Pharmacy Research Centre, University of South Australia, Adelaide, South Australia, Australia; 4 Australian Orthopaedic Association National Joint Replacement Registry, Adelaide, South Australia, Australia; 5 School of Public Health and Preventive Medicine, Monash University, Melbourne, Victoria, Australia; 6 School of Clinical Medicine, UNSW Medicine & Health, St George & Sutherland Clinical Campuses, Faculty of Medicine and Health, UNSW Sydney, New South Wales, Australia; 7 South Australian Health and Medical Research Institute, Adelaide, South Australia, Australia; 8 Orthopaedic Department, Westmead Private Hospital, Westmead, Sydney, New South Wales, Australia; 9 Orthopaedic Department, Lakeview Private Hospital, Baulkham Hills, Sydney, New South Wales, Australia; 10 Orthopaedic Department, Calvary John James Hospital, Deakin, Australian Capital Territory, New South Wales, Australia; 11 Department of Medicine, Faculty of Medicine, University of New South Wales, Sydney, New South Wales, Australia; 12 Department of Hematology, New South Wales Pathology, Kogarah Campus, Sydney, New South Wales, Australia; 13 Musculoskeletal Australia, Melbourne, Victoria, Australia; 14 Orthopaedic Department, Greenslopes Private Hospital, Greenslopes, Queensland, Australia; 15 Department of Surgery, Epworth Healthcare, University of Melbourne, Melbourne, Victoria, Australia; 16 Orthopaedic Department, Kareena Private Hospital, Sutherland, New South Wales, Australia; 17 Orthopaedic Department, Royal North Shore Hospital, St Leonards, New South Wales, Australia; 18 Sydney Musculoskeletal Health, University of Sydney, Sydney, New South Wales, Australia; 19 Orthopaedic Department, Hornsby and Kuringai Hospital, Hornsby, New South Wales, Australia; 20 Centre for Health Economics, Monash Business School, Monash University, Melbourne, Victoria, Australia; 21 Eastern Health Clinical School, Monash University, Box Hill, Victoria, Australia; 22 Orthopaedic Department, Royal Prince Alfred Hospital, Camperdown, New South Wales, Australia; 23 Bendigo Healthcare Group, Bendigo Hospital, Bendigo, Victoria, Australia; 24 Orthopaedic Department, Fremantle Hospital, Fremantle, Perth, Western Australia, Australia; 25 Orthopaedic Department, Canterbury Hospital, Canterbury, New South Wales, Australia; 26 Calvary Adelaide Hospital, Adelaide, South Australia, Australia; 27 Discipline of Medical Specialties, University of Adelaide, Adelaide, South Australia, Australia; 28 Orthopaedic Department, Fairfield Hospital, Fairfield, New South Wales, Australia; 29 Orthopaedic Department, St George Private Hospital, Kogarah, New South Wales, Australia; 30 Orthopaedic Department, Frankston Hospital, Frankston, Victoria, Australia; 31 Orthopaedic Department, The Prince Charles Hospital, Chermside, Queensland, Australia; 32 Orthopaedic Department, Launceston General Hospital, Launceston, Tasmania, Australia; 33 School of Medicine, St John of God Hospital and Barwon Health, Deakin University, Geelong, Australia; 34 Orthopaedic Department, Mater Hospital, Raymond Terrace, Brisbane, Queensland, Australia; 35 Orthopaedic Department, Prince of Wales Hospital, Randwick, New South Wales, Australia; 36 Orthopaedic Department, Nepean Hospital, Penrith, New South Wales, Australia; 37 Orthopaedic Department, Coffs Harbour Base Hospital, Coffs Harbour, New South Wales, Australia; 38 Orthopaedic Department, Western Health, Melbourne, Victoria, Australia; 39 The Kolling Institute, Faculty of Medicine and Health, The University of Sydney and the Northern Sydney Local Health District, Sydney, New South Wales, Australia; 40 Australian and New Zealand Intensive Care Research Centre, School of Public Health and Preventive Medicine, Monash University, Melbourne, Victoria, Australia; 41 St John of God Health Care, Perth, Western Australia, Australia; 42 Orthopaedic Department, Flinders Medical Centre, Bedford Park, South Australia, Australia; 43 Department of Medicine and Public Health, Flinders University, Adelaide, South Australia, Australia; 44 Orthopaedic Department, Sir Charles Gardiner Hospital, Perth, Western Australia, Australia; 45 Institute of Musculoskeletal Health, School of Public Health, The University of Sydney, Sydney, New South Wales, Australia

## Abstract

**Importance:**

Ischemic heart disease remains the leading cause of mortality following hip and knee arthroplasty. Due to its antiplatelet and cardioprotective properties, aspirin has been proposed as an agent that could reduce mortality when used as venous thromboembolism (VTE) prophylaxis following these procedures.

**Objective:**

To compare aspirin with enoxaparin in reducing 90-day mortality for patients undergoing hip or knee arthroplasty procedures.

**Design, Setting, and Participants:**

This study was a planned secondary analysis of the CRISTAL cluster randomized, crossover, registry-nested trial performed across 31 participating hospitals in Australia between April 20, 2019, and December 18, 2020. The aim of the CRISTAL trial was to determine whether aspirin was noninferior to enoxaparin in preventing symptomatic VTE following hip or knee arthroplasty. The primary study restricted the analysis to patients undergoing total hip or knee arthroplasty for a diagnosis of osteoarthritis only. This study includes all adult patients (aged ≥18 years) undergoing any hip or knee arthroplasty procedure at participating sites during the course of the trial. Data were analyzed from June 1 to September 6, 2021.

**Interventions:**

Hospitals were randomized to administer all patients oral aspirin (100 mg daily) or subcutaneous enoxaparin (40 mg daily) for 35 days after hip arthroplasty and 14 days after knee arthroplasty procedures.

**Main Outcomes and Measures:**

The primary outcome was mortality within 90 days. The between-group difference in mortality was estimated using cluster summary methods.

**Results:**

A total of 23 458 patients from 31 hospitals were included, with 14 156 patients allocated to aspirin (median [IQR] age, 69 [62-77] years; 7984 [56.4%] female) and 9302 patients allocated to enoxaparin (median [IQR] age, 70 [62-77] years; 5277 [56.7%] female). The mortality rate within 90 days of surgery was 1.67% in the aspirin group and 1.53% in the enoxaparin group (estimated difference, 0.04%; 95% CI, −0.05%-0.42%). For the subgroup of 21 148 patients with a nonfracture diagnosis, the mortality rate was 0.49% in the aspirin group and 0.41% in the enoxaparin group (estimated difference, 0.05%; 95% CI, −0.67% to 0.76%).

**Conclusions and Relevance:**

In this secondary analysis of a cluster randomized trial comparing aspirin with enoxaparin following hip or knee arthroplasty, there was no significant between-group difference in mortality within 90 days when either drug was used for VTE prophylaxis.

**Trial Registration:**

http://anzctr.org.au Identifier: ACTRN12618001879257

## Key Points

**Question:**

Is aspirin monotherapy more effective than enoxaparin in preventing mortality within 90 days following hip or knee arthroplasty procedures when either drug is used for venous thromboembolism (VTE) prophylaxis?

**Findings:**

In this secondary analysis from a cluster randomized, crossover trial that included 23 458 patients, there was no significant difference found in mortality within 90 days for patients allocated to aspirin compared with patients allocated to receive enoxaparin for VTE prophylaxis.

**Meaning:**

This secondary analysis of cluster randomized crossover trial found that in patients undergoing hip or knee arthroplasty procedures, aspirin was not associated with a lower mortality rate within 90 days of surgery compared with enoxaparin.

## Teaser

This secondary analysis of a cluster randomized crossover trial compares aspirin with enoxaparin as venous thromboembolism prophylaxis in reducing 90-day mortality for patients undergoing hip or knee arthroplasty.

## Introduction

Hip and knee arthroplasty are major orthopedic procedures that carry a risk of major complications, such as venous thromboembolism (VTE) and mortality. At least partly attributable to improvements in perioperative patient care and improved patient selection, mortality has decreased considerably over the last 2 decades.^1^ The current reported risk of 90-day mortality for patients undergoing primary, elective arthroplasty ranges between 0.03% and 0.10%.^2,3^ Risk factors for mortality have included increasing age, male sex, diabetes, liver disease, kidney disease, cerebrovascular disease, and previous cardiac ischemia.^4,5,6^ Ischemic heart disease has been demonstrated to be the leading cause of death within 30 and 90 days following surgery, whereas pulmonary embolism is a rare cause of early mortality.^6,7,8^

Given aspirin’s protective effects against myocardial infarction,^9^ it has been proposed as an agent that could reduce mortality compared with direct oral anticoagulants (DOACs) or low-molecular–weight heparin (LMWH) following hip and knee arthroplasty when used for VTE prophylaxis. However, most studies comparing perioperative mortality between aspirin and LMWH or DOACs have been observational and have produced inconsistent results.^10,11,12,13^

This study reports a planned secondary analysis of the CRISTAL trial,^2,14,15^ a registry-nested, cluster randomized, crossover, noninferiority trial of aspirin compared with enoxaparin, a LMWH, for VTE prophylaxis in hip or knee arthroplasty. Although enoxaparin was found to be more effective than aspirin in preventing symptomatic VTE within 90 days of total hip arthroplasty (THA) or total knee arthroplasty (TKA) for a diagnosis of osteoarthritis, the analysis for mortality in the primary analysis was limited by the low mortality rate in each group and the exclusion of patients undergoing partial and revision arthroplasty for nonosteoarthritis diagnoses, reducing the effective sample size for this outcome. The aim of this study was to investigate the hypothesis that aspirin would reduce mortality within 90 days compared with enoxaparin, as a secondary analysis of the CRISTAL trial.

## Methods

The CRISTAL trial was approved by the Sydney Local Health District human research ethics committee and by all relevant local ethic committees prior to commencement. All patients provided consent for provision and use of their data, and enrolled patients provided consent to be contacted postoperatively to collect outcome data for the primary study. A waiver of consent was granted by each ethics committee for the use of either study drug, as both drugs were considered common practice throughout Australia for prophylaxis prior to trial commencement. This secondary analysis follows the Consolidated Standards of Reporting Trials (CONSORT) reporting guideline for randomized trials.

### Trial Design

The CRISTAL trial was a cluster randomized, crossover, noninferiority trial performed across 31 hospitals in Australia between April 15, 2019, and December 18, 2020. It was nested within the Australian Orthopaedic Association National Joint Replacement Registry (AOANJRR). The primary aim was to determine whether aspirin was noninferior to enoxaparin in preventing symptomatic VTE after THA or TKA. The protocol and statistical analysis plan (which included this planned a priori study) are available in Supplement 1.

### Participating Hospitals and Patients

The clusters were defined as participating hospitals and were eligible if they had performed more than 250 hip or knee arthroplasty procedures the year prior to recruitment and agreed to follow the trial protocol. The trial protocol was applied to all patients undergoing any hip or knee arthroplasty procedure at participating hospitals during the course of the trial. Hospitals were advised to give all patients (enrolled or not) the study drug, except if they were not eligible to receive the study drug (eg, patients using preoperative DOACs, warfarin, or dual antiplatelet therapy or patients with a medical contraindication to either drug). Whether patients received their allocated treatment was assessed by the inpatient audit (which included enrolled and nonenrolled patients). This recorded whether patients received the correct drug dose, given daily, and commencing within 24 hours of surgery and were provided a discharge prescription.

The primary analysis included patients undergoing THA or TKA for a diagnosis of osteoarthritis only, who were eligible to receive the study drug.^2^ This secondary analysis broadened the inclusion criteria and included all patients undergoing any hip or knee arthroplasty procedure (including partial, revision, or total) for any indication at participating hospitals during the course of the study. Patients were included in this analysis regardless of whether they were enrolled into the primary study population or not.

### Randomization and Blinding

Following the protocol, each hospital was allocated to administer patients consecutive periods of a standard dose of enoxaparin or aspirin, with the initial treatment order being randomized. Hospitals were randomized in permuted blocks of 4 by statisticians from the South Australian Health and Medical Research Institute (SAHMRI), independent of study investigators. The randomization sequence was generated using an online application and provided to an unblinded data manager from SAHMRI. Each hospital was allocated to a treatment sequence by SAHMRI staff the week prior to enrolling patients. Hospitals were advised to crossover once the sample size for the first treatment group was met, which was monitored by AOANJRR staff.

Participating hospitals and patients were not masked to treatment allocation. Study investigators and the data safety monitoring board (DSMB) were masked to treatment assignment during the trial and all analyses.

### Interventions and Assessments

Patients in the aspirin group received oral aspirin 100 mg daily for 35 days after hip arthroplasty and for 14 days after knee arthroplasty, beginning within 24 hours postoperatively. Patients in the enoxaparin group received subcutaneous enoxaparin 40 mg daily for the same time periods, with the dose reduced to 20 mg for patients weighing less than 50 kg and for patients with an estimated glomerular filtration rate less than 30 mL/min/1.73 m^2^. All patients received intraoperative and postoperative intermittent pneumatic compression calf devices until mobile and compression stockings and were offered mobilization on day 0 or day 1 postoperatively.

Data from all patients included in this study were obtained through the AOANJRR. The AOANJRR routinely collects demographic and outcome data for all patients undergoing hip or knee arthroplasty in Australia, including age, sex, body mass index (BMI; calculated as weight in kilograms divided by height in meters squared), American Society of Anesthesiologists (ASA) classification,^16^ joint replaced (hip or knee), type of arthroplasty (partial, total, or revision), indication for surgery, and the outcomes for mortality and revision joint arthroplasty. These data were available for all included patients (enrolled and nonenrolled) in this study. Additional data on preoperative anticoagulant use and postoperative occurrence of VTE, bleeding, readmission and joint-related reoperation were only available for enrolled patients and were therefore reported in the previous publication^2^ and are not reported in this study.

Mortality data within 90 days of surgery were collected through linkage between the AOANJRR and the National Death Index. The National Death Index is a national database administered by the Australian Institute of Health and Welfare that relies on data submitted by jurisdictional death registers. A quality statement on the national death index is available elsewhere.^17^

### Outcome Measures and Sample Size Calculation

The primary outcome for this study was all-cause mortality within 90 days of surgery. No a priori power calculation was performed for this secondary analysis from the CRISTAL trial. Using the sample size included in each group, a post hoc sample size calculation was performed at a power of 80%.

### Interim Analysis

No interim analysis was planned. However, due to concerns raised from 1 ethics committee regarding a serious adverse event, an independent DSMB was assembled, and an interim analysis was performed. After the second interim analysis (December 2020), the DSMB recommended stopping patient enrollment, as the stopping rule had been met for the primary outcome of the CRISTAL trial (a between-group difference for symptomatic VTE within 90 days at *P* < .001).

### Statistical Analysis

All analyses were performed using SAS statistical software version 9.4 (SAS Institute) and R statistical software version 4.1.0 (R Project for Statistical Computing). Data were analyzed from June 1 to September 6, 2021.

Patients were analyzed according to their randomization group. There were no missing data on mortality within 90 days. Due to the higher mortality associated with arthroplasty for a diagnosis of fracture, a planned subgroup analysis was performed according to whether the patient underwent surgery for a diagnosis of fracture or not.

The analysis for the primary outcome tested the between-group difference for mortality within 90 days for superiority. This was estimated using cluster summary methods^18,19^ and used the same methods as those used for the secondary outcomes in the primary study.^2^ These estimates adjusted for unequal cluster sizes and for clusters that had incomplete crossover or did not achieve crossover. These have been shown to be appropriate for cluster randomized crossover trials with rare outcomes and the intracluster and interperiod correlation coefficients expected in this trial. The unit of analysis was considered to be the hospital, with outcomes reported at the individual level. The same statistical methods were used for the primary outcome and for the subgroup analyses. A 95% CI was used to detect a statistically significant difference between groups. A post hoc survival analysis for each group was performed using the Kaplan-Meier method, including the log-rank *P* value and a number at risk table. The log-rank *P* value was 2-sided, and statistical significance was set at *P* < .05.

## Results

**Figure 1.**
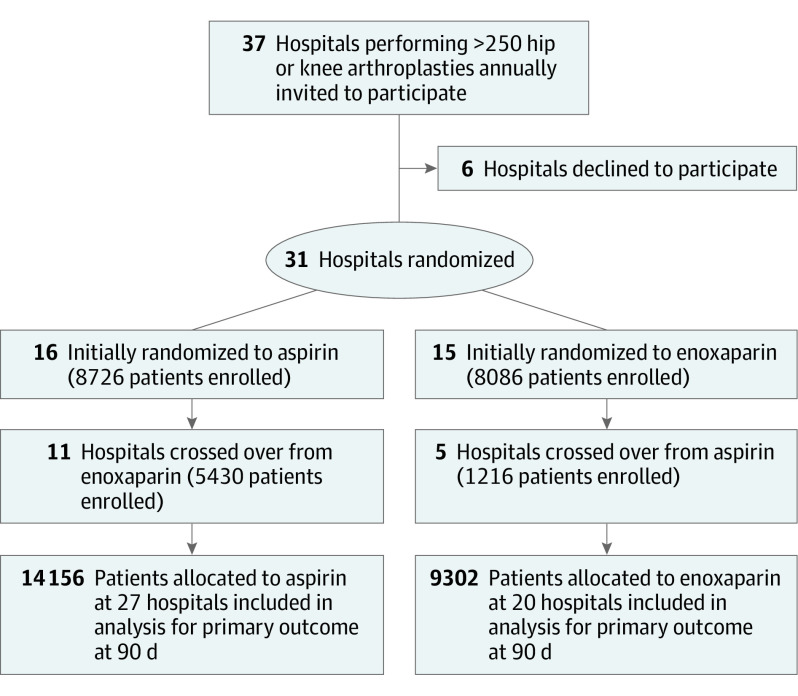
Site and Patient Enrollment, Randomization, and Follow-up of Participating Hospitals and Patients by Treatment Allocation Group

Thirty-one hospitals were randomized between April 15, 2019, and August 12, 2019. There were 21 public and 10 private hospitals included, and the median (range) number of hip and knee arthroplasty procedures at these hospitals performed in the year prior to study commencement was 580 (250 to 1617). All 31 hospitals were randomized, 16 to administer aspirin and 15 to administer enoxaparin. Between April 15, 2019, and December 18, 2020, 16 of 31 hospitals completed enrollment for their initially assigned therapy and crossed over to the alternate therapy prior to trial cessation (11 crossed over to aspirin and 5 crossed over to enoxaparin). The remaining 15 hospitals did not crossover prior to early cessation of the trial (Figure 1). During the study period, there were 23 458 patients who underwent an arthroplasty procedure at CRISTAL participating hospitals. There were 14 156 patients (median [IQR] age, 69 [62-77] years; median [IQR] BMI 30.0 [26.2-34.6]; 7984 [56.4%] female) allocated to receive aspirin and 9302 patients (median [IQR] age, 70 [62-77] years; median [IQR] BMI, 29.9 [26.1-34.5]; 5277 [56.7%] female) allocated to receive enoxaparin. Patient characteristics were similar between groups (Table 1).

**Table 1.** Baseline Patient Characteristics, According to Treatment Allocation

Characteristic	Patients, No. (%)	
	Aspirin (n = 14 156)	Enoxaparin (n = 9302)
Age, median (IQR), y	69 (62-77)	70 (62-77)
BMI, median (IQR)^a^	30.0 (26.2-34.6)	29.9 (26.1-34.5)
Sex		
Female	7984 (56.4)	5277 (56.7)
Male	6172 (43.6)	4025 (43.3)
ASA classification^b^		
1	621 (4.4)	383 (4.1)
2	6589 (46.7)	4196 (45.2)
3	6329 (44.9)	4273 (46.0)
4	563 (4.0)	436 (4.7)
5	4 (<0.1)	4 (<0.1)
Joint replacement		
Hip arthroplasty	6577 (46.4)	4316 (46.4)
Knee arthroplasty	7579 (53.6)	4986 (53.6)
Type of surgery		
Primary total	11 615 (82.1)	7509 (80.7)
Primary partial	1306 (9.2)	960 (10.3)
Primary resurfacing	60 (0.4)	53 (0.6)
Revision	1173 (8.3)	779 (8.4)
Other	2 (<0.1)	1 (<0.1)
Indication		
Osteoarthritis	11 123 (78.6)	7160 (77.0)
Inflammatory	127 (0.9)	79 (0.8)
Avascular necrosis	213 (1.5)	158 (1.7)
Fracture	1484 (10.5)	1077 (11.6)
Other	1209 (8.5)	828 (8.9)

Abbreviations: ASA, American Society of Anesthesiologists; BMI, body mass index (calculated as weight in kilograms divided by height in meters squared).

^a^ Includes data for 13 483 patients in the aspirin group and 8792 patients in the enoxaparin group.

^b^ Includes data for 14 106 patients in the aspirin group and 9292 patients in the enoxaparin group.

There were 1005 patients audited for inpatient drug adherence (543 in the aspirin group and 462 in the enoxaparin group, including enrolled and nonenrolled patients), and 178 patients were audited for postdischarge drug adherence (71 in the aspirin group and 107 in the enoxaparin group, only including enrolled patients). Overall adherence rates were 521 of 614 patients (85%) in the aspirin group and 491 of 569 patients (86%) for the enoxaparin group.

### Primary Outcome

Mortality within 90 days occurred in 236 of 14 156 patients (1.67%) in the aspirin group and in 142 of 9302 patients (1.53%) in the enoxaparin group. The difference between groups was not statistically significant (estimated difference, 0.04%; 95% CI, −0.38% to 0.46%) (Table 2).

**Table 2.** Primary Outcomes and Subgroup Analysis for Patients by Treatment Allocation

Outcome	Events, No./total No. of patients (%)		Estimated treatment difference % (95% CI)	*P* value
	Aspirin (n = 14 154)	Enoxaparin (n = 9299)		
Death within 90 d	236/14 156 (1.67)	142/9302 (1.53)	0.04 (−0.38 to 0.46)	.85
Subgroup analysis for mortality^a^				.38
Nonfracture diagnosis	63/12 832 (0.49)	34/8316 (0.41)	0.05 (−0.67 to 0.76)	.90
Fracture diagnosis	173/1324 (13.1)	108/986 (11.0)	1.0 (−1.0 to 3.0)	.33

^a^ *P* for interaction = .38.

### Subgroup Analysis

Subgroup analysis for the primary outcome based on diagnosis for surgery (fracture or not) demonstrated no difference between subgroups (*P *for interaction = .38). Death within 90 days occurred in 63 of 12 832 patients (0.49%) in the aspirin group and in 34 of 8316 patients (0.41%) in the enoxaparin group (estimated difference, 0.05%; 95% CI, −0.67% to 0.76%) for patients with a nonfracture diagnosis. For patients with a diagnosis of fracture, 173 of 1324 patients (13.1%) in the aspirin group and 108 of 986 patients (11.0%) in the enoxaparin group died within 90 days (estimated difference, 1.0%; 95% CI −1.0% to 3.0%).

### Survival Analysis

**Figure 2.**
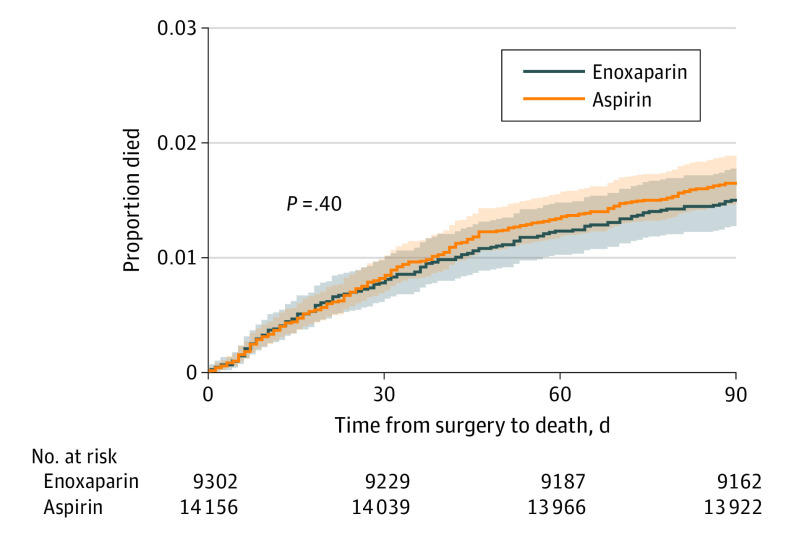
Kaplan-Meier Analysis of Mortality by Allocation Group

The median (IQR) time to mortality was 30 (13-51) days in the aspirin group and 28 (11-52) days in the enoxaparin group. There was no between-group difference found on log-rank testing (*P* = .40) (Figure 2).

### Post hoc Power Calculation

A post hoc power calculation was performed to determine what between-group difference the study was able to detect. Using the sample size included each group and accounting for the trial’s cluster randomized design, the study had 80% power to detect a difference for the primary outcome (for superiority of 1 treatment over the other) of 0.7%.

## Discussion

In this planned secondary analysis from the CRISTAL cluster randomized crossover trial, there was no between-group difference in mortality within 90 days for patients receiving either aspirin or enoxaparin for VTE prophylaxis following hip or knee arthroplasty. Mortality rates were lower in the enoxaparin group for all analyses, although none reached statistical significance. These findings complement the results of the primary study by demonstrating that enoxaparin reduces symptomatic VTE following these procedures, without resulting in an increase in mortality compared with aspirin.

Three randomized clinical trials and multiple observational studies have included short-term mortality (either 30 or 90 days) as a secondary outcome when investigating the use of aspirin for VTE prophylaxis following THA or TKA. The results from the 3 randomized trials support our finding of similar mortality outcomes for aspirin vs other chemoprophylaxis agents or placebo in reducing mortality following elective hip or knee arthroplasty. However, none of these trials compared aspirin monotherapy with other agents used for VTE prophylaxis (eg, DOACs, LMWH, or warfarin). The Pulmonary Embolism Prevention (PEP) trial compared aspirin with placebo and demonstrated no between-group difference in all-cause mortality or symptomatic VTE within 90 days for 2648 patients undergoing elective arthroplasty.^20^ Two randomized trials by Anderson et al^3,21^ compared aspirin for extended prophylaxis with either rivaroxaban after 5 days (3424 patients) or dalteparin after 10 days (778 patients) and did not demonstrate any between-group differences in mortality within 90 days. A fourth randomized trial in patients with trauma injuries compared aspirin monotherapy with LMWH for VTE prophylaxis and demonstrated similar results to this study and those reported in CRISTAL: symptomatic VTE rates were higher in patients allocated to aspirin but there was no between-group difference observed for mortality within 90 days.^22^

Three observational studies (including 59 747 patients,^23^ 30 499 patients,^24^ and 30 270 patients^25^) reported that mortality within 90 days did not differ between patients receiving aspirin and those receiving other forms of prophylaxis (eg, rivaroxaban, enoxaparin, dalteparin, or warfarin). In contrast, a 2019 observational study by Rondon et al^13^ (including 31 133 patients) reported that aspirin was associated with a significantly lower risk of mortality within 30 days and 1 year after THA or TKA compared with other forms of prophylaxis (ie, apixaban, rivaroxaban, dabigatran, dipyridamole, clopidogrel, fondaparinux, heparin, lepirudin, rivaroxaban, ticlopidine, or warfarin). However, the study by Rondon et al^13^ was conducted over 17 years (between 2000 to 2017). A temporal analysis was not conducted to evaluate time as a potential confounder in this study or the 3 other long-duration observational studies (which recruited patients over similar time periods). Multiple studies have demonstrated that mortality rates following THA and TKA have decreased over the course of the last 20 years. In a study using National Joint Registry data from England and Wales, early mortality after hip and knee arthroplasty for osteoarthritis decreased by nearly 50% between 2003 and 2011.^26,27^ Similar findings have been published from Australia, Sweden, and Denmark.^1,28,29^ During this time, the use of aspirin for VTE prophylaxis (compared with other agents) has increased.^30,31^ Thus, the lower risk of mortality in these observational studies may be attributed to general improvements in perioperative care over the last 2 decades rather than aspirin use.^1,26,27,28,29^

The strengths of this study include its prospective design, well-balanced demographic data between groups and broad inclusion criteria with respect to indication for surgery and type of surgery, which increase the generalizability of the results. The protocol was applied to all participating hospitals, and the results reflect the effect of implementing the use of these drugs at a department level.

### Limitations

This study has some limitations. First, outside of the audits, no data were available on whether patients received their intended treatment, and as-treated analyses could not be performed. However, the hospital audits conducted during the trial included both enrolled and nonenrolled patients and demonstrated high adherence rates. Second, some included patients may have received additional forms of anticoagulation (eg, DOACs, warfarin, or dual antiplatelet medications). Although the number of these patients could not be measured, it was assumed that they were balanced between groups (as they were for the primary study). Third, data on cause of death were not available. If available, this may have provided more specific information regarding the contribution of VTE prophylaxis to mortality. Fourth, despite the large sample size, due to the early cessation of the trial, the study was likely underpowered, given the between-group difference found and the results of the post hoc power calculation. Fifth, data on race and ethnicity were not collected for each group. However, the randomized study design would be expected to minimize unknown confounding.

## Conclusions

This secondary analysis of a cluster randomized crossover clinical trial found that among patients undergoing hip or knee arthroplasty, there was no difference in mortality within 90 days between patients allocated to enoxaparin vs aspirin used for VTE prophylaxis. In conjunction with the results from the primary CRISTAL study,^2^ these findings suggest that enoxaparin reduces the risk of symptomatic VTE without increasing the risk of mortality in comparison to aspirin after hip or knee arthroplasty.

## Notes

Supplement 1.
Trial Protocol and Statistical Analysis Plan

Supplement 2.
Data Sharing Statement
