# Supplementary material for: Effect of Aspirin vs Enoxaparin on 90-Day Mortality in Patients Undergoing Hip or Knee Arthroplasty: A Secondary Analysis of the CRISTAL Cluster Randomized Trial
Source: JAMA Netw Open. 2023 Jun 9;6(6):e2317838. doi: 10.1001/jamanetworkopen.2023.17838 (PMC10257098; doi:10.1001/jamanetworkopen.2023.17838)
Supplement: Supplement 1. — Trial Protocol and Statistical Analysis Plan [file jamanetwopen-e2317838-s001.pdf]

INITIAL PROTOCOL: 28 September 2018

STUDY PROTOCOL

CRISTAL: A cluster randomised, crossover, non-inferiority trial of aspirin compared to low molecular weight heparin for venous thromboembolism prophylaxis and safety in hip or knee arthroplasty, a registry nested study

TABLE OF CONTENTS

This supplement contains the following items:

i.	Initial Protocol	Pages 3 – 31
ii.	Final Protocol	Pages 32 – 65
iii.	Summary of Changes	Pages 66 – 73

Study Protocol

CRISTAL: a cluster randomised, crossover, non-inferiority trial of aspirin compared to low molecular weight heparin for venous thromboembolism prophylaxis in hip or knee arthroplasty, a registry nested study.

1. **Administrative information**

1.1. Registration

CRISTAL will be registered with the Australian and New Zealand Clinical Trials Registry (anzctr.org.au).

1.2. Funding

This study is fully and solely funded by a 4-year Medical Research Futures Fund Lifting Clinical Trials and Registries Capacity Grant (Application ID: APP1152285) awarded in January 2018. The funding source had no role in the design of this study and will not have any role during its execution, analyses, interpretation of the data, dissemination or decision to publish.

1.3. Contributors

Ian A Harris (IAH)
Professor of Orthopaedic Surgery UNSW Sydney
Deputy Director, AOANJRR (Australian Orthopaedic Association National Joint Replacement Registry)
Executive Member of ANZMUSC (Australian and New Zealand Musculoskeletal Clinical Trials Network)
Director, Whitlam Orthopaedic Research Centre

Stephen E Graves (SEG)
Director, AOANJRR
Adjunct Professor or Orthopaedic Surgery, University of South Australia

Rachelle Buchbinder (RB)
NHMRC Senior Principal Research Fellow, Director, Monash Department of Clinical Epidemiology, Cabrini Institute and Professor, Department of Epidemiology and Preventive Medicine, School of Public Health and Preventive Medicine, Monash University, Chair, Executive Committee, ANZMUSC (Australia and New Zealand Musculoskeletal Clinical Trials Network), rheumatologist.

Sam Adie (SA)
Orthopaedic Surgeon
St. George and Sutherland Clinical School, University of New South Wales

Verinder Sidhu (VS)
Orthopaedic Registrar
PhD candidate

Justine Maree Naylor (JMN)
Senior Principal Research Fellow (Orthopaedics), South Western Sydney Local Health District
Director, Whitlam Orthopaedic Research Centre

Ilana Ackerman (IA)
Victorian Health and Medical Research Fellow and Associate Professor (Research),
Department of Epidemiology and Preventive Medicine, Monash University

Beng Hock Chong (BHC)
Haematologist, St George Hospital

Nicole Pratt (NP)
Lead statistician, UniSA

Richard de Steiger (RdS)
Professor of Surgery, University of Melbourne
Deputy Director of the Australian Orthopaedic Association National Joint Replacement Registry

Anthony Harris (AH)
Health Economist

Amber Hansen
Consumer

Maggie Cripps
Consumer

Michelle Lorimer
Senior statistician, AOANJRR

Liddy Griffith
Senior Manager Clinical Research, Heart Health

Ornella Clavisi
Consumer

Steve Webb
Methodologist
Senior Staff Specialist, Intensive Care Medicine, Royal Perth Hospital, Clinical Professor, School of Medicine and Pharmacology & School of Population Health, University of Western Australia, Clinical Professor, Department of Epidemiology and Preventive Medicine, School of Public Health and Preventive Medicine, Monash University.

IAH conceived the study. IAH, SEG, RB, SA, JMN, IA, BHC, NP, RdS and AH are Chief Investigators on the MRFF grant. NP is the primary statistician. AH is the primary health economist. All contributors participated in protocol development.

1.4. Sponsor

South Western Sydney Clinical School, Faculty of Medicine, UNSW Sydney
Liverpool Hospital, Elizabeth St, LIVERPOOL, NSW, 2170, Australia

1.5. Study Coordination

Committees

**Committee**	**Members**	**Responsibilities**
Writing Committee	IAH, SG, RB, NP, SA, VS	Protocol development and publication<br>Preparation of principal publications (primary and secondary outcomes)
Steering committee	All investigators listed above (contributors)	Final protocol approval<br>Study oversight<br>Principal publication approval
Trial management committee	IAH, SEG, VS, SA, RdS, Project Coordinator, AOANJRR Registry Manager, AOANJRR PROMs project manager, SAHMRI rep (LG), RdS, SAHMRI IT, SAHMRI Data Management, SAHMRI Statistician	Integration with AOANJRR PROMs program<br>Ethics approval<br>Site liaison (recruitment and maintenance)
Data Quality Committee	IAH, Project Manager, ML, RdS	Data management<br>Data quality audits
Outcome Verification Committee	ACORN Manager, JN, IA, BHC	Validating imaging verification of DVT and PE reported during patient follow up

Coordinating Centre
The day to day management of the trial will be the responsibility of the Australian Orthopaedic Association National Joint Replacement Registry (AOANJRR) and South Australian Health & Medical Research Institute (SAHMRI).

Other expert subgroups may be established throughout the project to advise on specific elements and make recommendations should the need arise.

1.6. Abbreviations

AOANJRR	Australian Orthopaedic Association National Joint Replacement Registry
AAOS	American Academy of Orthopaedic Surgeons
ACORN	Arthroplasty Clinical Outcomes Registry
DVT	Deep Venous Thrombosis
ICJME	International Committee of Medical Journal Editors
LMWH	Low Molecular Weight Heparin
NICE	National Institute of Health and Care Excellence
NOAC	Novel Oral Anticoagulant
PE	Pulmonary Embolus
PROMs	Patient Reported Outcome Measures
THA	Total Hip Arthroplasty
TKA	Total Knee Arthroplasty
HA	Hip Arthroplasty
KA	Knee Arthroplasty
VTE	Venous Thromboembolism

2. Introduction

2.1. Background

Over 100,000 total hip and total knee arthroplasty (THA, TKA) procedures are performed each year in Australia.[1] Venous thromboembolism (VTE) comprises deep venous thrombosis (DVT) and pulmonary embolus (PE) and is a recognised serious complication of hip and knee arthroplasty surgery. Patients undergoing THA and TKA receive chemoprophylaxis for VTE prevention, with most patients in Australia receiving either low molecular weight heparin (LMWH) or aspirin (manuscript in preparation).

Guideline recommendations and surgeon preference for VTE prophylaxis vary due to a lack of evidence regarding the comparative safety and effectiveness of these two common chemoprophylaxis agents. Aspirin is a low cost, over-the-counter, safe medication that is easy to take (one oral tablet daily). LMWH requires daily injection (often requiring professional or family support), is more expensive and requires prescription, but has a larger body of evidence of effectiveness. Previous studies comparing LMWH and aspirin have been underpowered for effectiveness and for safety.

Currently, practice guidelines provide conflicting recommendations for VTE prophylaxis. The National Institute of Health and Care Excellence (NICE) guidelines (United Kingdom) now (2018) recommend using LMWH, aspirin or Novel Oral Anticoagulants (NOACs) for VTE prophylaxis in TKA (aspirin is not recommended for THA) whereas aspirin was not recommended in the previous verision.[2] In the US, two main guidelines are used: those recommended by the American College of Chest Physicians (ACCP)[3] and those produced by the American Association of Orthopaedic Surgeons (AAOS).[4] Both recommend the use of LMWH, NOACs or aspirin. Previously, the ACCP guidelines recommended against aspirin whereas the AAOS guidelines recommended its use. As of 2012, both guidelines now allow the use of aspirin for VTE prophylaxis, and as a result the prevalence of aspirin prescription has increased.[5] The Australian National Health and Medical Research Council guidelines (2011) did not recommend aspirin, however these guidelines were rescinded in 2016 as they were considered outdated.[6]

A number of systematic reviews (including data from up to 22 trials) have summarised the evidence for VTE prophylaxis in joint arthroplasty, but most do not assess aspirin, despite being commonly used and recommended by some practice guidelines.[3-4, 7-13]

Two small systematic reviews were found, including data from six pharmacological trials that had aspirin as a comparator.[14,15] In both reviews, the evidence was dominated by one trial of 778 patients comparing aspirin to LMWH in THA.[16] This trial was stopped early due to poor recruitment. Furthermore, all patients in the trial received LMWH for the first 10 days before random allocation to aspirin or continued LMWH. This does not reflect the way that aspirin is commonly used in Australia as aspirin is commenced during the acute care period. Another five trials were also described, including a total of 936 patients, but these trials were small, measured different outcomes, and were subject to bias.[14] Both reviews concluded that there is insufficient evidence to support recommendations on the use of

aspirin, and suggest larger trials are needed.[14,15] A recent large trial compared aspirin to rivaroxaban (a NOAC) for VTE prophylaxis in TKA and THA. A total of 3424 patients were recruited in this cluster-randomised trial, however both groups were treated with rivaroxaban for the first 5 days before being randomised to aspirin or rivaroxaban for the following 2-4 weeks.[17]

While studies using administrative datasets should be interpreted with caution due to risk of coding errors, incomplete data and difficulty fully adjusting for possible confounding, two studies of aspirin using large administrative datasets have been reported. The first, from the US, used data from 93,804 patients undergoing elective total knee replacement surgery.[18] The study compared early (30 day) mortality and VTE between patients given warfarin, LMWH and aspirin, adjusted for patient factors (age, sex, race, VTE risk, comorbidities), institution factors (size, urban/rural) and a separate propensity score. No difference was found in the mortality rates or rates of post-operative bleeding complications between the three groups, and there was no difference in the rate of VTE comparing LMWH to aspirin. A study using data from the National Joint Registry for England, Wales, Northern Ireland and the Isle of Man analysed data from 108,584 patients undergoing THA comparing LMWH to aspirin for VTE prophylaxis using multivariable modelling and propensity score matching.[19] The adjusted analysis showed no significant difference in mortality up to 90 days post-operatively but this difference became significant (favouring LMWH) on propensity score matching. There was no difference in VTE complications or re-operations (up to 90 days) between groups. The reported rates of VTE were very low, possibly due to under-detection.

The existing uncertainty regarding the relative safety and effectiveness of these different medications to prevent VTE following arthroplasty and inconsistencies in available clinical practice guidelines likely contribute to widespread clinical practice variation in Australia. A national survey[20] and recent large cohort study involving 1,900 patients from 19 institutions across Australia (manuscript under preparation) show that nearly all surgeons use some form of chemoprophylaxis, with approximately 80% using LMWH and nearly half using aspirin (approximately 40% of patients had more than one drug). The survey indicated that those using LMWH were more likely to do so for fear of litigation.[20] Aspirin does not require a prescription, is easier for patients to take (tablet rather than injection), is safe and is inexpensive. Therefore, establishing non-inferiority would provide patients with a preferred, effective, safe, cheaper and simpler method of VTE prophylaxis compared to LMWH.

The Australian Orthopaedic Association National Joint Replacement Registry (AOANJRR) was established in 1999 and reports on revision surgery and mortality after joint arthroplasty in Australia, with close to complete national coverage. The AOANJRR has established a system to directly capture data entered by patients pre- and post-operatively; this system is a platform for the conduct of clinical trials and is incorporated as part of the AOANJRR (not a standalone project). The proposed CRISTAL trial will be embedded within the Clinical Trials Platform of the AOANJRR.

2.2. Choice of comparators

Wide practice variation is evident for VTE chemoprophylaxis in Australia due to both surgeon uncertainty in the evidence for effect, and differences in surgeon preference. Aspirin and LMWH are the two most frequently used drugs for VTE prophylaxis in THA and

TKR in Australia. Alternatives, such as warfarin and unfractionated heparin are not commonly used in Australia and are not reflected in practice guidelines in this country. NOACs are an alternative to LMWH and aspirin but are not commonly used in Australia. Given aspirin's popularity, ease of use, safety profile and low cost but lack of evidence of comparative effectiveness against the most commonly recommended drug (LMWH), we considered aspirin to be the most suitable comparator to LMWH for this trial.

2.3. Study hypothesis

Aspirin is non-inferior to LMWH for prevention of VTE after THA and TKA surgery.

2.4. Primary aim

To compare the effectiveness and safety of aspirin to LMWH in preventing symptomatic VTE after primary elective TKA and THA for osteoarthritis.

2.5. Secondary aims

- To compare the effectiveness and safety of aspirin to LMWH in preventing symptomatic VTE after TKA and THA (elective *primary and revision*, for any reason)
- To compare the effectiveness and safety of aspirin to LMWH in preventing symptomatic VTE after *revision* TKA and THA
- To compare the cost effectiveness of aspirin to LMWH for VTE prophylaxis after primary elective TKA and THA for osteoarthritis
- Compare the safety (proportion of non-VTE complications) between groups
- An extension of the primary analysis for: THA and TKA separately; unilateral and bilateral separately; below-knee DVT, above-knee DVT and PE separately

2.6. Trial design

A pragmatic multicentre, cluster-randomised, crossover, non-inferiority study with a primary endpoint of patient-reported VTE at 90 days.

3. Methods

3.1. Setting

Eligible hospitals (public and private) performing HA and KA in Australia. The study will be nested within the AOANJRR Clinical Trials Platform, an electronic platform for recruitment and data collection for patients undergoing joint replacement surgery.

3.2. Eligibility

Hospital level

- Departmental (or surgeon group) agreement to participate in the study and adhere to study protocols
- Use of intermittent calf compression device intra-operatively and post-operatively until mobile
- Offer mobilisation day 1 post-operatively or earlier
- No change in other practices or protocols relevant to VTE over the course of the study (e.g. tourniquet use, anaesthetic type).

Patient level

- Adults (age 18 and older)
- Receiving elective primary or revision KA or HA for any reason

Exclusion criteria

As a pragmatic study, all patients undergoing elective revision and primary arthroplasty for any reason during the study period will be included. The only exclusion for a department is if the volume of primary elective THA and TKA for osteoarthritis is less than 222 joints per year (as this renders the site unable to recruit the required sample size required for the primary analysis within the prespecified study timeframe). For the primary analysis, the following exclusions will apply (at a patient level):

- Partial joint replacement
- Revision surgery
- Non-osteoarthritis diagnosis
- Use of warfarin, NOAC or dual antiplatelet therapy pre-operatively

3.3. Intervention

Each site will be allocated to two consecutive periods of standard protocol of aspirin and standard protocol of LMWH for VTE prophylaxis with the order of the two periods determined by randomisation at a 1:1 ratio on an open label basis.

Each site will adhere to the initially randomised protocol for a time period based on surgical volume aiming for 222 patients eligible for the primary analysis per group. Maximum time period for each group will be 12 months. Total recruitment (all arthroplasties) to each group is expected to be 250-300 patients.

Patients will be informed of the trial during initial data entry. Patients will be specifically asked at the time of study entry for consent for follow up, for use of their data in research, and use of linked data to measure and verify surgical outcomes (Appendix 1). Patients will not be individually consenting to be randomised to either aspirin or LMWH, as both drugs represent standard practice and randomisation is not at the patient level. Further details on the consent process is listed in protocol (section 4.3).

Patients will be followed at 90 (75-105) days and 6 (5-7) months, electronically with telephone back up. To ensure minimal inconvenience a maximum of three reminders will be sent to the patient to complete their follow up CRISTAL questions.

- 1st reminder – upon registration
- 2nd reminder – 3 days from the first reminder
- 3rd reminder - 4 days from the second reminder

There will be no change to usual medical follow up (clinic attendance, investigations etc.) except that routine venous imaging is not to be used (currently not recommended and not commonly used).

Aspirin will be administered orally at 100mg (85-150 mg permitted if previously prescribed) daily for 28-35 days (hips) or 10-14 days (knees) commencing the day of or day after surgery.

LMWH will be administered as enoxaparin (Clexane) 40mg subcutaneously daily, for 28-35 days (hips) or 10-14 days (knees) commencing the day of or day after surgery. Patients will be taught to self-administer while in hospital. For those unable to self-administer, the injections will be given by family members, a community nursing service or their local doctor, depending on local arrangements.

3.4. Adherence

Patients may discontinue the drug if they have an allergy or adverse event related to the drug.

The study drug may be withheld if post-operative wound ooze continues to 36 hours post-operatively, with recommencement 48 hours later if settled.

Inpatient adherence during the acute care period will be determined by a chart audit of a sample of patients from a sample of hospitals.

Post-discharge adherence will be determined by patient report during follow up at 90 days.

3.5. Concomitant care

All patients will have intermittent compression devices intra-operatively and post-operatively until mobile. All patients will be offered mobilisation on day one post-surgery unless surgically or medically contraindicated.

Patients taking (non-aspirin) oral anti-platelet therapy pre-operatively may have their medication withheld for one week pre-operatively if advised by their treating doctor and will recommence their usual medication (in addition to any study medication) at day 7 post-operatively or when safe to do so.

Routine doppler screening for DVT (in asymptomatic patients) will not be permitted by participating sites.

Patients taking aspirin (85-125mg daily) pre-operatively will take this drug in the usual dose post-operatively in place of the study drug for those in the aspirin group, and in addition to LMWH for those in the LMWH group. The aspirin may be stopped pre-operatively if advised by their treating doctors.

3.6. Primary outcome

The primary outcome is verified, symptomatic VTE (DVT or PE) at 90 day follow up.
All reports of VTE will be verified by contact with treating doctors and institutions and obtaining any imaging reports. VTE will be deemed verified by the independent Outcome Verification Committee. The absence of VTE will be verified in a random sample of 222 patients reporting an absence of VTE, by auditing treating doctors and institutions. Patients will be asked if they are still taking anticoagulant medication at 90 day follow up and those who are will be probed for the reasons for ongoing anticoagulation.

3.7. Secondary outcomes

1. Safety. Any bleeding complication leading to reoperation. Any reoperation or readmission related to the surgery or anticoagulation (defined as haemorrhage, infection, dislocation, manipulation, fracture, loosening or migration of implant, death, other)
2. Costs. Cost of anticoagulation, cost of hospital stay, need for further health resource utilisation (e.g. nurse or GP visits) and complications (see list below)
3. Adherence. Proportion of patients taking the drug continuously (no more than 2 consecutive days missed) for the recommended minimum period
4. PROMs. Health-related quality of life (EQ5D-5L), Oxford hip and knee scores and patient-rated satisfaction and improvement

Complications will be classified into the following groups by the Outcome Verification Committee:

- Readmission related to the original surgery or associated treatment (including bleeding and VTE related)
- Reason for readmission (infection, dislocation, stiffness, fracture, wound dehiscence, implant loosening, migration or failure, wound bleeding, other bleeding)
- Reoperation on the same joint
- Type of reoperation (treatment of infection, reduction of dislocation, manipulation under anaesthesia, fracture treatment, wound repair, implant loosening, migration or failure, non-joint related surgery)

- DVT below knee
- DVT above knee
- PE
- Death

3.8. Participant timeline

Time Point	Data Collection Questions and Instruments
Pre-operative	Current anticoagulation use (yes/no and drug)<br>Age<br>Sex<br>Joint (hip or knee)<br>Side<br>Unilateral vs bilateral<br>Primary or revision<br>ASA grade<br>BMI<br>Oxford Hip or Knee Score<br>EQ-5D-5L<br>EQVAS<br>Low back pain<br>Joint pain (numeric rating scale 0-10)<br>Expectations (pain and improvement)
90 days	VTE (DVT or PE)<br>Adherence (did you use pills or injections to prevent a blood clot post-operatively, for how long?)<br>Current use of anticoagulants (yes/no, which one)<br>Complications (asked individually, as per complication list)
6 months	VTE (DVT or PE)<br>Complications (asked individually, as per complication list)<br>Oxford Hip or Knee Score<br>EQ-5D-5L<br>Joint pain (scale 0-10)<br>Satisfaction with outcome of surgery<br>Patient-rated improvement

<u>EQ-5D-5L (Appendix 2)</u>
The EQ-5D is a standardised measure of health status developed by the EuroQol Group in order to provide a simple, generic measure of health for clinical and economic appraisal. (22) The survey includes 5 health outcome domains that can be summarised into a utility score. (21) These include:

- Mobility
- Self-care
- Usual Activities
- Pain/Discomfort
- Anxiety/Depression

There are five descriptive sentences under each heading and patients are directed to tick one box that best describes their health on that day. There is also a visual analogue scale (VAS) that addresses health state. (21)

Oxford Hip or Knee Scores (Appendix 3)
The Oxford hip (OHS) and knee (OKS) scores were developed in the mid-1990s. The scores were developed to assess the outcome of hip and knee replacements as well as shoulder surgery (including shoulder replacement) and were designed to be completed by patients in order to minimise potential bias. (23) Both two instruments include 12 questions to assess a patient's capacity to undertake general activities of daily living, about their affected hip or knee.

Pre-operative anticoagulation use (Appendix 4)
Questions listed below will be presented pre-operatively to determine any history of VTE and current use of Anti-coagulant medications. The questions were reviewed and approved by consumer representatives and will be specific to the medications and the site of DVT.

1. Do you normally take (or are you currently taking) blood thinning medication routinely?
2. Do you know what blood thinning medication are taking?
3. Have you ever been diagnosed with a clot in your legs (Deep Vein Thrombosis (DVT) or lungs (Pulmonary Embolism (PE)?
4. When did your most recent (or only) clot in your legs (DVT) or lungs (PE) occur?

Post-operative VTE symptoms and occurrence (Appendix 4)
The following questions will be presented post-operatively at both 90 days and 6 months to gauge patient's surgery outcome Questions 1 to 3 are only specific for the 90 days data collection point and will not be asked on the 6 months data collection point.

1. Did you take your POST-surgery blood thinning medication (to avoid blood clots) after leaving hospital following your joint replacement operation?
2. Do you know what blood thinning medication are taking?
3. How many days di you take the blood thinning medication after your (HIP/KNEE) replacement operation?
4. Since your joint replacement surgery, have you been diagnosed with a blood clot in your legs (DVT) or lungs (PE)?
5. Have you had any further surgery on your replaced joint (apart from when it was put in)?
6. Please select the reason(s) for the additional surgery/surgeries? (select all that apply)
7. Have you had any serious bleeding from anywhere else in your body not related to your joint replacement?

3.9. Sample size

A pooled analysis of 47 RCTs and cohort studies estimated the incidence of symptomatic VTE to be approximately 1%. The previous RCT of aspirin versus LMWH used an event rate of 1.5% and a minimum clinically important difference of 2.0%. A recent large cohort study

of 1900 hip and knee replacement patients from 19 institutions across Australia (manuscript under preparation) showed an incidence of symptomatic VTE of 2.9% up to 90 days post surgery.

Using an estimated overall event rate of 2%, and a non-inferiority margin (for aspirin compared to LMWH) of 1% and using a power of 80% and a one-sided significance level of 0.05, 4,800 patients would be required for an individual randomised trial. For a cluster randomised crossover trial, we will require 8,000 patients to account for correlation between and within clusters. We will therefore recruit a minimum of 400 patients (200 per treatment arm) from each of 20 institutions (clusters).

Allowing for 10% loss to follow up (estimated at 5% based on current similar systems), 8,888 eligible patients (a minimum of 222 per group from a minimum of 20 sites) will be recruited.

3.10. Recruitment

Hospitals will be approached individually by the lead CI and the study team, as appropriate. A site will be considered eligible if they can recruit 222 eligible patients (for the primary analysis) within 12 months, and recruit for up to 24 months. Departmental (or surgeon group) agreement with the study protocol and the individual treatment protocols (for each group) will be required. Sites where a subgroup of attending surgeons agree to participate will be included if the number of eligible patients for that group of surgeons per year it at least 222. A site investigator will be nominated for each site

3.11. Randomisation

Each site will be randomised with a 1:1 allocation with a computer generated random sequence. Simple randomisation will be used (no use of blocks, no stratification). The allocation will refer to the first intervention.

3.12. Blinding

Sites will not be blinded to group allocation. Patients will be aware of a study comparing different treatments for VTE prevention but will not know the specific details of the study and will therefore be blind to the specific interventions and outcomes of the trial. Outcomes will be self-reported with verification of the primary outcome by the Outcome Verification Committee. Where outcome reporting is by phone (back up for failure to capture patients reported outcomes electronically and where verification is performed) those outcome assessors will be blinded to group allocation.

The Outcome Verification Committee will be presented with deidentified cases for assessment. The statistical analysis will be blinded. The Writing Committee will be blinded and will prepare separate manuscripts based on the possible group allocations.

3.13. Data collection

Data collection for baseline data and follow up at 90 days and 6 months will be patient-reported electronically (via tablet, phone or computer) using direct data entry. For patients

not responding to email and SMS follow up, telephone contact will be used to administer the surveys verbally.

Pre-Operative

When collected electronically by patients, data will be directly entered into the AOANJRR Clinical Trials Platform.

Some patients may not have direct access to the internet. This is especially a factor for older patients and patients from lower socioeconomic groups[27]. Another group who could potentially be excluded from implementing this approach to data collection are non-English speaking patients. To overcome these barriers patients will be given the option to nominate a 'proxy' e.g. family member or friend to assist them with completing the instruments and receive reminders electronically on their behalf. Information will be collected on whether the patient had assistance to complete the CRISTAL questions and these data will be reviewed during the analysis.

There will be various methods and procedures implemented at the different hospitals to register patients and request them to complete the CRISTAL data online. Ideally the patients will complete pre-operative CRISTAL data immediately when first approached, however, this may not always be possible. There is functionality built into the system to email a patient the link to the website to complete the CRISTAL requirements at a time that is convenient for them.

Some hospitals participating in CRISTAL already routinely collect PROMs and wish to continue to do so using their own systems. In these cases, the AOANJRR will work with the hospital to simplify the data collection process and avoid duplication of collection. A data sharing agreement will be entered into between the hospital and the AOANJRR whereby data can be exported from the current system and imported into the AOANJRR Clinical Trials Platform. There will be a secure file sharing facility established within the web application to ensure secure transfer of confidential information. The data provided will be reviewed by the data manager prior to upload into the database to confirm quality and completeness.

Post-Operative

Follow up will be by telephone until the electronic data capture system is built and telephone follow up will be used as back up for the electronic follow up once in place. Patients will be able to login and complete their 90 day follow-up from 75 days and 6-month follow-up from 5 months.

The Arthroplasty Clinical Outcomes Registry (ACORN) has been contracted to complete the follow-up phone calls. ACORN was selected because this Registry already collects PROMs centrally for hospitals, predominately in NSW, and the staff have expertise in this area.

3.14. Data management

Data quality will be checked monthly under the supervision of the Data Quality Committee.

3.15. Statistical analysis

The primary analysis will be restricted to elective primary THA and TKA for osteoarthritis and will test between-group difference in the proportion of cases developing VTE within 90 days for non-inferiority of aspirin at a margin of 1%, on an intention to treat basis. The primary analysis will use a multi-level modelling (MLM) framework. This framework effectively models the complex correlation structure of the cluster randomised crossover design but utilizes the power available from individual level data. The model allows for correlation of patients within hospitals and also correlation between study periods within the same hospital. Multiple imputation will be used to account for missing outcome data, should a patient be lost to follow-up at 90 days. Possible confounders will be gathered from routine AOANJRR data (including age, sex, baseline health pain and function, diagnosis and surgical factors).

This analysis will be performed on the primary and secondary outcomes.

Secondary analyses:

1. An extension of the primary analysis to patients receiving elective Knee Arthroplasty KA and Hip Arthroplasty (HA) (primary and revision, for any reason)
2. An extension of the primary analysis to patients receiving revision KA and HA
3. Economic analysis (see below)
4. Compare the safety (proportion of non-VTE complications) between groups, as a total and for each individual complication
5. An extension of the primary analysis for: THA and TKA separately; unilateral and bilateral separately; below-knee DVT, above-knee DVT and PE separately
6. Compare VTE and complication rates in those receiving LMWH alone to those receiving LMWH and oral anticoagulation (patients in the LMWH group already taking oral anti-platelet therapy).

Cost effectiveness of prophylactic aspirin compared to LMWH will be evaluated from a health system perspective. Data for resource use associated with treatments and complications will be taken from trial data within the AOANJRR, supplemented by linked MBS and PBS data and valued at total public prices (Medicare and national average diagnosis related group [DRG] costs). Costs include drug acquisition, out of hospital doctor fees charged, tests, in hospital medical and pharmaceutical costs post-surgery. Survival at one year and quality of life measured using EQ5D at baseline, 90 days and six months will allow calculation of differences in Quality Adjusted Life Years (QALYs) between groups. We will calculate the cost per QALY for each treatment comparison as the difference in mean costs divided by the difference in mean outcomes (quality adjusted survival as QALYs) over the duration of the trial, using mixed model regression analysis to adjust for differences at baseline and clustering by site. We will extrapolate from the trial evidence and simulate the long-term comparative cost effectiveness of each treatment including long term complication and outcomes stage to age 101 years in a decision analytic model.

3.16. Data monitoring and cleaning

A separate Data Quality Committee will be established to monitor data management and quality.

A separate safety monitoring committee will not be established and no stopping rules will be used as both interventions are commonly used and recommended treatments. No interim analysis will be performed; this will reduce the chance of early stopping due to spurious findings. Adverse events (separate to complications listed under secondary outcomes) will be monitored by the Trial Management Committee).

3.17. Auditing and Data validation

Positive outcomes (reported VTE) reported by patients will be confirmed by contacting hospitals or treating doctors and retrieving imaging reports. These will be adjudicated by the Outcome Verification Committee.

A sample of negative outcomes (patients reporting the absence of VTE) will be verified in a similar manner in a random sample of patients with negative outcomes.
Complications will be adjudicated by the Outcome Verification Committee.
ANZMUSC will audit the study.

The AOANJRR Data Linkage project will be used to test the accuracy of outcome reporting (readmission, re-operation, drug prescriptions). The AOANJRR also links to the National Death Index (NDI) twice a year (February and September). If a patient, who has participated in the CRISTAL project, is flagged as deceased in the AOANJRR database this can also be transferred to the CRISTAL system and no further contact with be made, reducing distress for families.

4. Ethics and dissemination

4.1. Ethics approval

The study will be submitted to Sydney Local Health District (RPAH Zone) human research ethics committee for approval. Following approval, the study will be submitted to local ethics committees and Research Governance Offices as required for each site. Refer to the Table 1 for sites.

Table 1: List of Sites for Ethics approval by Sydney Local Health District (RPAH Zone)

State	Hospital
NSW	Canterbury Hospital
NSW	Coffs Harbour
NSW	Fairfield Hospital
NSW	Gosford Public Hospital
NSW	Hornsby Ku-ring-gai Hospital
NSW	Nepean Hospital
NSW	Prince of Wales Hospital
NSW	Royal Prince Alfred Hospital (Institute of Rheumatology and Orthopaedic Surgery)
NSW	Royal North Shore Hospital
NSW	Ryde Hospital
NSW	Sutherland Hospital
QLD	Mater Adults Hospital
QLD	Prince Charles Hospital
SA	Flinders Medical Centre
VIC	Bendigo Hospital
VIC	Epworth Private Hospital
VIC	Frankston Hospital
VIC	University Hospital Geelong Barwon Health
VIC	Western Hospital Footscray
VIC	Western Hospital Williamstown
WA	Fremantle Hospital
WA	Osborne Park Hospital
WA	Royal Perth Hospital
WA	Sir Charles Gairdner Hospital

4.2. Amendments

Any modifications to the protocol which may impact on the conduct of the study, potential benefit of the patient or may affect patient safety, including changes of study objectives, study design, patient population, sample sizes, study procedures, or significant administrative aspects will require a formal amendment to the protocol. Such amendment will be agreed upon by the Steering Committee and approved by the Ethics Committee prior to implementation and site notification.

Administrative changes of the protocol are minor corrections and/or clarifications that have no effect on the way the study is to be conducted. These administrative changes will be agreed upon by Trial Management Committee and will be documented in a memorandum. The Ethics Committee/IRB may be notified of administrative changes at the discretion of Trial Management Committee.

4.3 Consent to project participation

Individual consent is not being sought for randomisation or use of the study drugs. This is because randomisation is not occurring at the patient level and because both study drugs represent current standard practice. Consent is being sought for the collection and use of patient data, as per standard protocol for the AOANJRR Clinical Trials Platform. The Clinical Trials Platform uses the same consent process (and near identical data collection) as the AOANJRR PROMS Pilot Project which has received ethics approval (Reference: X18-0057 & HREC/18/RPAH/90).

Consent to AOANJRR Clinical Trials Platform

Consent will be obtained electronically. All data collection for this project is electronic. This provides efficiency and effectiveness (less error) and allows a better way to impart information relevant to the consent process. The participant information and consent form will be displayed on the screen. It contains all elements required for a consent form (see Appendix 1). The information under each statement will be expandable. Patients will be provided the option to ‘agree to the statement’ or ‘learn more’. If the patient agrees they will be navigated to the next statement. If the patient chooses to learn more the additional information will be displayed. Once all statements have been agreed to the patient will be able to choose whether they give consent or no longer wish to participate in the study. If the patient consents to participate they will be directed to the next page where they can complete the required pre-operative CRISTAL questions relevant to their procedure. If the patient chooses not to consent after the initial registration, then all personal information collected at registration will be deleted from the database. The only data that will be retained is:

- Hospital Name (if available)
- Surgeon Name (if available)
- Date of registration If the patient elects to withdraw at the time of their post-operative assessment, then no further follow-up will be undertaken.

Electronic Consent Process Flowchart

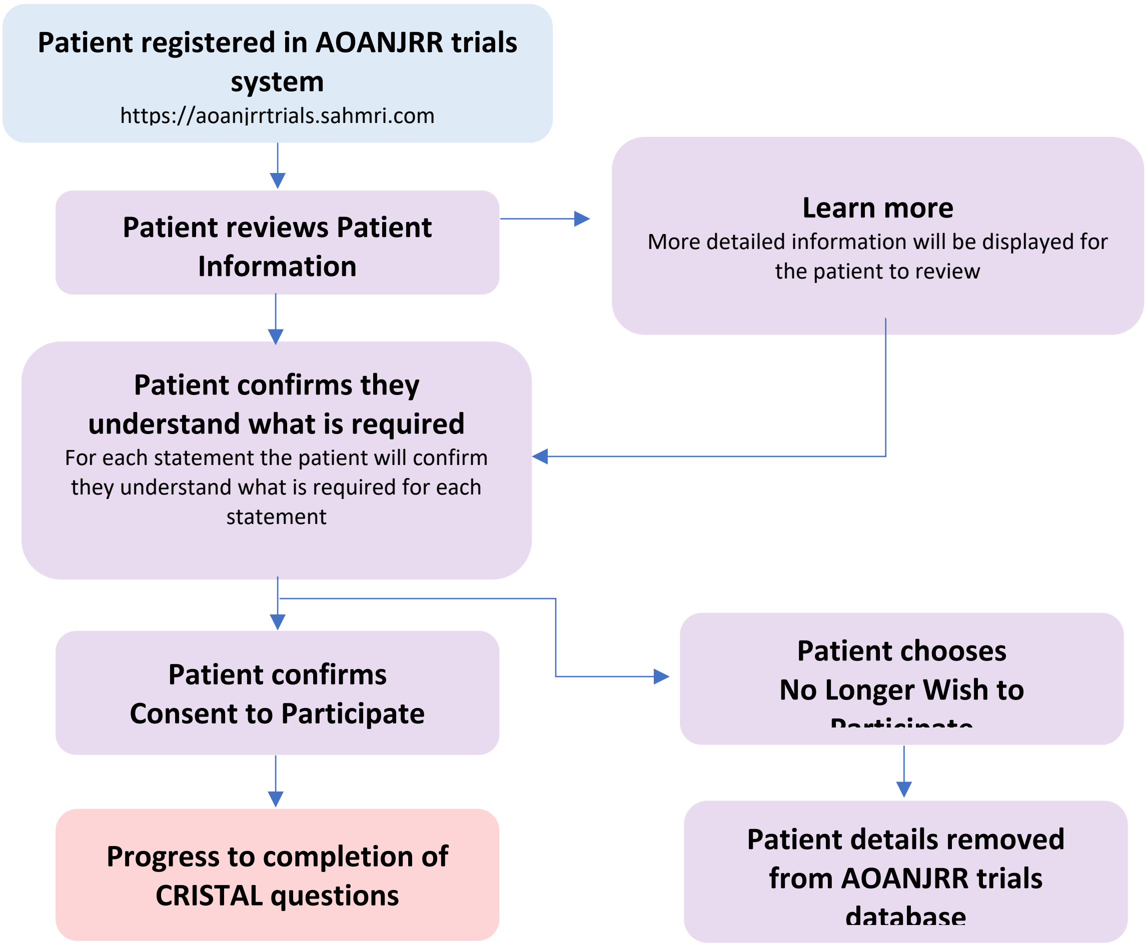

Consent to share data

After completing the pre- and post-operative CRISTAL questions, patients will be given the option (at each time point) to share their data with their treating surgeon. If the patient consents for their results to be shared the treating surgeon will be able to download patient level data from the CRISTAL system. If patients do not consent to share these data, their information will not be provided to the surgeon.

Waiver of Consent

We are requesting waiver of consent for two different aspects of this project. Firstly, we require waiver of consent for the ‘registration’ process which involves getting basic contact information from potential participants so that they can be contacted and formally invited to participate and consent. An identical process of registration has been previously approved for the AOANJRR PROMs pilot. Secondly, we request waiver of consent for randomisation and treatment with the study drugs (the intervention) that make up the CRISTAL project.

Each request for waiver of consent are listed below: firstly, for the ‘registration’ process (clinical trials platform), and secondly for specific involvement (randomisation and treatment) in the CRISTAL study.

Waiver of consent Part 1: patient registration prior to initial patient contact.

The request for waiver of consent only applies in some instances. Specifically, some patients will be registered in the system by hospital administrative staff or their treating surgeon. Once this registration occurs the patient will subsequently be sent an email by the AOANJRR to obtain consent electronically prior to completing the CRISTAL questions. The data that will be stored within the AOANJRR between registration and consent includes:

- Patient First Name
- Patient Middle Name
- Patient Surname
- Date of Birth
- Postcode
- Hospital
- Surgeon name
- The joint that will be operated on (Hip, Knee, Shoulder)
- The side that will be operated on (Left, Right, Both)
- Patient contact details such as phone number and email address

It is important to emphasise that the AOANJRR will collect almost all the registration information when it is provided at the time of surgery except for phone number and email address. We believe this request satisfies the criteria as detailed in the National Statement on Ethical Conduct in Human Research (2013, chapter 2.3) for providing a waiver of consent. This waiver of consent is only for collecting information through the registration process and not to the completion of the CRISTAL instruments.

a. Involvement in the research carries no more than low risk

   - This is a low risk project particularly as the waiver is specifically required to defer involvement in the project after the registration as a mechanism to ensure that patients that take this option can complete their involvement in the project at a time that is most suitable to them.

b. the benefits from the research justify any risks of harm associated with not seeking consent

   - There is no risk of harm associated with storing the patient’s details prior to collecting consent. The Registry will receive almost all of this data at the time of the procedure. It is being requested to enhance participant convenience. The AOANJRR is a declared Federal Quality Assurance Activity and all data is managed in accordance with that declaration which includes the use of high level security systems.

c. it is impracticable to obtain consent (for example, due to the quantity, age or accessibility of records)

   - It is not feasible to collect patient consent prior to the registration as it is necessary to link the electronic consent to the individual patient identified by the registration process. If the consent is completed prior to registration then the consent will be unidentified.

d. there is no known or likely reason for thinking that participants would not have consented if they had been asked

   - Patients will verbally consent to have their details recorded at registration and this will be subsequently confirmed prior to completion of the CRISTAL Questions instruments. It is the AOANJRR experience that very few patients are reluctant to have their data included in the Registry.

e. there is sufficient protection of their privacy

   - The AOANJRR is a declared Federal Quality Assurance Activity
   - Systems are in place to ensure individual patient data remains confidential
   - A third-party security review and penetration testing will be undertaken prior to commencement of data collection

f. there is an adequate plan to protect the confidentiality of data

   - SAHMRI which is the organisation responsible for managing AOANJRR data has existing security systems, policies and procedures in place as well as software barriers to protect personal information and ensure confidentiality. These systems are already in place for data contained within the AOANJRR and the CRISTAL data will be treated identically (see Appendix 5).

g. in case the results have significance for the participants' welfare there is, where practicable, a plan for making information arising from the research available to them (for example, via a disease-specific website or regional news media)

   - Patients will be able to review their own results and how they compare to the national average via online dashboards.

h. the possibility of commercial exploitation of derivatives of the data or tissue will not deprive the participants of any financial benefits to which they would be entitled

   - The AOANJRR is a not for profit organisation which does not use the data it collects for commercial gain.

i. the waiver is not prohibited by State, federal, or international law

   - There are no applicable laws prohibiting this waiver.

<u>Waiver of consent Part 2: participant randomisation and treatment under the CRISTAL study.</u>

The CRISTAL study is seeking a waiver of individual consent for the intervention proposed by the study (the administration of either aspirin or LMWH). It is recommended that all patients who undergo THA or TKA require chemoprophylaxis to prevent VTE, with-holding or not giving chemoprophylaxis is considered against the current standard of care. We believe this request satisfies the criteria as detailed in the National Statement on Ethical Conduct in Human Research (2013, chapter 2.3) for providing a waiver of consent.

a. involvement in the research carries no more than low risk

- This is a low risk project as it involves interventions that are currently used in standard practice (aspirin and LMWH for VTE prophylaxis). The additional questions asked of patients is not considered of sufficient risk or burden to justify specific consent as they are also part of routine practice (follow up health and complication questionnaires) for most sites. Furthermore, randomisation is not at the patient level – it occurs at the site level (cluster randomisation).

b. the benefits from the research justify any risks of harm associated with not seeking consent

- There is no additional harm from this study as both intervention arms are standard practice. There may be an imbalance of harms between groups, but this study is necessary to determine this, and this highlights the benefits that will arise from the research as there is currently insufficient evidence to guide practice which has resulted in widespread practice variation.

c. it is impracticable to obtain consent (for example, due to the quantity, age or accessibility of records)

- Specific consent for CRISTAL would require an additional consent process (in addition to the consent for the use of data). This would make entry into the research cumbersome and confusing and would likely lead to a higher proportion of patients abandoning data entry, reducing the scientific validity of the study.

d. there is no known or likely reason for thinking that participants would not have consented if they had been asked

- Patients currently receive VTE prophylaxis without consent as part of standard practice and we consider the process of this trial to be similar to standard practice.

e. there is sufficient protection of their privacy

- The AOANJRR is a declared Federal Quality Assurance Activity
- Systems are in place to ensure individual patient data remains confidential
- A third-party security review and penetration testing will be undertaken prior to commencement of data collection

f. there is an adequate plan to protect the confidentiality of data

- SAHMRI which is the organisation responsible for managing AOANJRR data has existing security systems, policies and procedures in place as well as software

barriers to protect personal information and ensure confidentiality. These systems are already in place for data contained within the AOANJRR Clinical Trials platform and the CRISTAL data will be treated identically (see Appendix 5)

g. in case the results have significance for the participants' welfare there is, where practicable, a plan for making information arising from the research available to them (for example, via a disease-specific website or regional news media)

    - It is the intent of the researchers that the results of the CRISTAL study will be synthesised and published as a clinical trial in a peer reviewed journal. The only trial data of relevance to the patients will be the development of adverse events or VTE, which will be known to them at the time. Other data collected as part of the Registry will be made available to patients as per usual practice.

h. the possibility of commercial exploitation of derivatives of the data or tissue will not deprive the participants of any financial benefits to which they would be entitled

    - The CRISTAL study is receiving no industry or pharmaceutical corporation support and does not aim to make any financial profit or gain during the trial or after publication of the results. It will not deprive participants of any financial benefits.

i. the waiver is not prohibited by State, federal, or international law

    - There are no applicable laws prohibiting this waiver.

4.4 Confidentiality

AOANJRR is required to have highly secure data protection systems to secure the identified information which it currently holds as this is an absolute requirement under its Federal Quality Assurance Activity.

SAHMRI has been contracted to build the AOANJRR Trials which will be utilised for CRISTAL. SAHMRI has existing security systems, policies and procedures in place as well as software barriers to protect personal information and ensure confidentiality. (Appendix 5)

As this will be a fully electronic system, accessible online, which will store patient's personal and contact details, additional security activities have been included in the AOANJRR Trials system development:

- A third-party security review of the infrastructure and application design, prior to development starting
- A penetration test of the application prior to commencement of data collection

<u>Patient Confidentiality</u>
All patient data will be managed in accordance with the Guidelines for the Protection of Privacy in the Conduct of Medical Research. Patient contact details will only be used for the

purpose for which they were collected and will be stored securely and confidentially. Patients will not be identified in any reports, manuscripts or presentations derived from the CRISTAL project.

Surgeon Confidentiality
No individual surgeons will be identified in any reports or manuscripts.

4.5 Risk to Patients

As patients will be treated with the standard protocol for both LWMH and Aspirin, this study poses no foreseeable risk, harm or discomfort to patients beyond the inconvenience associated with completing the study questionnaires at three-time points. We recognise the burden of survey completion but also recognise that patient outcome collection is now becoming a standard part of patient care and will be standard practice in most sites recruiting for CRISTAL.

As patient follow-up is a requirement of this project all efforts will be made not to contact the relatives of a deceased participant. The AOANJRR links to the National Death Index (NDI) twice a year (February and September). If a patient, who has participated in the CRISTAL project, is flagged as deceased this will be transferred to the AOANJRR trials when the procedure date is linked. This will stop any automated and manual reminders being triggered

4.6 Declaration of interests

Ian Harris (IH), Stephen Graves (SG), Richard de Steiger (Rds) and Michelle Lorimer are employed by the AOANJRR. Nicole Pratt (NP)'s salary is partly supported by the MRFF grant received for CRISTAL.

4.7 Data access

All principal investigators involved in data analysis will have access to deidentified datasets. All principal investigators involved in subcommittees will have access to relevant deidentified data necessary for undertaking their specific role (e.g. outcome validation).

4.8 Additional care

As both interventions are standard, recommended practice, no additional treatment will be provided for participants.

4.9 Dissemination

A writing committee will be established to write the principal papers (primary and secondary outcomes). Dissemination will be by peer reviewed journal publication, conference presentation and through media. All study findings will be reported, regardless of statistical significance or the size or direction of effect.

Study findings will be released to participating sites and investigators.

Input will be sought into guideline development by state and national bodies (e.g. ACSQHC). The results of the study are expected to be published in a journal with high impact and to be of interest to a wide audience (beyond orthopaedics and haematology, including hospitalists and public health). They are expected to have clinical importance and statistical power that will enable the results to influence practice, which currently lacks studies on this size and quality.

4.10 Implementation

Surgeons will be surveyed prior to commencement (separate study) to assess their willingness to change practice based on the results of the trial, allowing for current practice, study findings, experience, gender.

Following the study, practice change at departmental and surgeon level will be measured for each surgeon at each site by assessing departmental and individual surgeon prophylaxis methods.

Practice change more broadly will be assessed through data linkage, assessing the increase or decrease in post-operative LMWH prescriptions.

4.11 Authorship

Authorship for principal papers will be by the members of the writing committee and the CRISTAL Study Group (consisting of all investigators according to the authorship guidelines of the ICMJE).

5. Statement for compliance with NHMRC National Statement on Ethical Conduct of Research Involving Humans

This study will be conducted in accordance with the ethical principles that have their origin from the Declaration of Helsinki and are consistent with ICH/GCP. This study will comply with National Health and Medical Research Council (NHMRC) National Statement on Ethical Conduct in Research Involving Humans.

6. References

1. Australian Orthopaedic Association National Joint Replacement Registry. Annual Report. Adelaide: AOA; 2017. 2017, Australian Orthopaedic Association
2. NICE (United Kingdom). Venous thromboembolism in over 16's: reducing the risk of hospital-acquired deep vein thrombosis or pulmonary embolism. 2018
3. Falck-Ytter Y, Francis CW, Johanson NA et al. Prevention of VTE in Orthopedic Surgery Patients. Chest 2012;141(2):e278S-e325S
4. Mont MA, Jacobs JJ, Boggio LN et al: Preventing venous thromboembolic disease in patients undergoing elective hip and knee arthroplasty. In: Journal of the American Academy of Orthopaedic Surgeons. vol. 19: Am Acad Ortho Surgeons; 2011: 768-776
5. Shah SS, Satin AM, Mullen JR et al. Impact of recent guideline changes on aspirin prescribing after knee arthroplasty. Journal of orthopaedic surgery and research 2016;11(1):123
6. National Health and Medical Research Council. Clinical Practice Guideline for the Prevention of Venous Thromboembolism- Rescinded. Melbourne: National Health and Medical Research Council; 2009
7. Gómez-Outes A, Terleira-Fernández AI, Suárez-Gea ML et al. Dabigatran, rivaroxaban, or apixaban versus enoxaparin for thromboprophylaxis after total hip or knee replacement: systematic review, meta-analysis, and indirect treatment comparisons. BMJ 2012;344(e3675-e3675
8. Adam SS, McDuffie JR, Lachiewicz PF et al. Comparative effectiveness of new oral anticoagulants and standard thromboprophylaxis in patients having total hip or knee replacement: a systematic review. Annals of Internal Medicine 2013;159(4):275-284.
9. Cao YB, Zhang JD, Shen H et al. Rivaroxaban versus enoxaparin for thromboprophylaxis after total hip or knee arthroplasty: a meta-analysis of randomized controlled trials. European journal of clinical pharmacology 2010;66(11):1099-1108.
10. Cohen A, Drost P, Marchant N et al. The Efficacy and Safety of Pharmacological Prophylaxis of Venous Thromboembolism Following Elective Knee or Hip Replacement. Clinical and Applied Thrombosis/Hemostasis 2012;18(6):611-627.
11. Harenberg J, Marx S, Dahl OE et al. Interpretation of endpoints in a network meta-analysis of new oral anticoagulants following total hip or total knee replacement surgery. Thrombosis and haemostasis 2012;108(5):903-912.
12. Neumann I, Rada G, Claro JC et al. Oral direct Factor Xa inhibitors versus low-molecular-weight heparin to prevent venous thromboembolism in patients undergoing total hip or knee replacement: a systematic review and meta-analysis. Annals of Internal Medicine 2012;156(10):710-719.
13. Turun S, Banghua L, Yuan Y et al. A systematic review of rivaroxaban versus enoxaparin in the prevention of venous thromboembolism after hip or knee replacement. Thrombosis research 2011;127(6):525-534
14. Wilson DGG, Poole WEC, Chauhan SK. Systematic review of aspirin for thromboprophylaxis in modern elective total hip and knee arthroplasty. Bone and Joint Journal 2016;Aug;98-B(8)(1056-1061.
15. Drescher FS, Sirovich BE, Lee A et al. Aspirin versus anticoagulation for prevention of venous thromboembolism major lower extremity orthopedic surgery: a systematic review and meta-analysis. Journal of hospital medicine 2014;9(9):579-585

16. Anderson DR, Dunbar MJ, Bohm ER et al. Aspirin versus low-molecular-weight heparin for extended venous thromboembolism prophylaxis after total hip arthroplasty: a randomized trial. Annals of Internal Medicine 2013;158(11):800-806
17. Anderson DR, Dunbar MJ, Murnaghan J. Aspirin or Rivaroxaban for VTE Prophylaxis after Hip or Knee Arthroplasty. N Eng J Med 2018;378(8):699-707
18. Bozic KJ, Vail TP, Pekow PS et al. Does aspirin have a role in venous thromboembolism prophylaxis in total knee arthroplasty patients? The Journal of arthroplasty 2010;25(7):1053-1060
19. Jameson SS, Charman SC, Gregg PJ et al. The effect of aspirin and low-molecular-weight heparin on venous thromboembolism after hip replacement: a non-randomised comparison from information in the National Joint Registry. J Bone Joint Surg Br 2011;93(11):1465-1470
20. Molnar RB, Jenkin DE, Millar MJ et al. The Australian arthroplasty thromboprophylaxis survey. J Arthroplasty 2012;27(2):173-179
21. Rolfson O., Bohm E., Franklin P., Lyman S., Denissen G., Dawson J., Dunn J., Eresian Chenok K., Dunbar M., Overgaard S. Patient-reported outcome measures in arthroplasty registries: Report of the Patient-Reported Outcome Measures Working Group of the International Society of Arthroplasty Registries Part II. Recommendations for selection, administration, and analysis. Acta orthopaedica. 2016;87(sup1):9-23.
22. The EuroQol Group. EuroQol-a new facility for the measurement of health-related quality of life. Health Policy. 1990;16(3):199-208.
23. Murray D., Fitzpatrick R., Rogers K., Pandit H., Beard D., Carr A., Dawson J. The use of the Oxford hip and knee scores. Bone & Joint Journal. 2007;89(8):1010-4.24. Roos E.M., Lohmander L.S. The Knee injury and Osteoarthritis Outcome Score (KOOS): from joint injury to osteoarthritis. Health and Quality of Life Outcomes. 2003;1(64)
25. Nilsdotter A.K., Lohmander L.S., Klässbo M., Roos E.M. Hip disability and osteoarthritis outcome score (HOOS) - validibity and resposiveness in total hip replacement. BMC Musculoskeletal Disorders. 2003;4(10).
26. Lyman S., Lee Y.Y., Franklin P.D., Li W., Mayman D.J., Padgett D.E. Validation of the HOOS, JR: A Short-form Hip Replacement Survey. Clinical orthopaedics and Related Research. 2016;476(6):1472-1482.
27. Lyman S., Lee Y.Y., Franklin P.D., Li W., Cross M.B., Padgett D.E. Validation of the KOOS, JR: A Short-form Knee Arthroplasty Outcomes Survey. Clinical Orthopaedics and
28. Jenkins P., Sng S., Brooksbank K., Brooksbank A. Socioeconomic deprivation and age are barriers to the online collection of patient reported outcome measures in orthopaedic patients. The Annals of The Royal College of Surgeons of England. 2016;98(1):40-4
29. Valier A.R.S., Jennings A.L., Parsons J.T., Vela L.I. Benefits of and barriers to using patient-rated outcome measures in athletic training. Journal of athletic training. 2014;49(5):674-8

STUDY PROTOCOL

CRISTAL: A cluster randomised, crossover, non-inferiority trial of aspirin compared to low molecular weight heparin for venous thromboembolism prophylaxis and safety in hip or knee arthroplasty, a registry nested study

Study Protocol

CRISTAL: a cluster randomised, crossover, non-inferiority trial of aspirin compared to low molecular weight heparin for venous thromboembolism prophylaxis in hip or knee arthroplasty, a registry nested study.

1. Administrative information

1.1. Registration

CRISTAL has been registered with the Australian and New Zealand Clinical Trials Registry (anzctr.org.au, ACTRN12618001879257).

1.2. Funding

This study is fully and solely funded by a 4-year Medical Research Futures Fund Lifting Clinical Trials and Registries Capacity Grant (Application ID: APP1152285) awarded in January 2018. The funding source had no role in the design of this study and will not have any role during its execution, analyses, interpretation of the data, dissemination or decision to publish.

1.3. Contributors

Ian A Harris (IAH)
Professor of Orthopaedic Surgery UNSW Sydney
Deputy Director, AOANJRR (Australian Orthopaedic Association National Joint Replacement Registry)
Executive Member of ANZMUSC (Australian and New Zealand Musculoskeletal Clinical Trials Network)
Director, Whitlam Orthopaedic Research Centre

Stephen E Graves (SEG)
Director, AOANJRR
Adjunct Professor of Orthopaedic Surgery, University of South Australia

Rachelle Buchbinder (RB)
NHMRC Senior Principal Research Fellow, Director, Monash Department of Clinical Epidemiology, Cabrini Institute and Professor, Department of Epidemiology and Preventive Medicine, School of Public Health and Preventive Medicine, Monash University, Chair, Executive Committee, ANZMUSC (Australia and New Zealand Musculoskeletal Clinical Trials Network), Rheumatologist.

Sam Adie (SA)
Orthopaedic Surgeon
St. George and Sutherland Clinical School, University of New South Wales

Verinder Sidhu (VS)
Orthopaedic Registrar
PhD candidate

Justine Maree Naylor (JMN)
Senior Principal Research Fellow (Orthopaedics), South Western Sydney Local Health District
Director, Whitlam Orthopaedic Research Centre

Ilana Ackerman (IA)
Victorian Health and Medical Research Fellow and Associate Professor (Research), Department of Epidemiology and Preventive Medicine, Monash University

Beng Hock Chong (BHC)
Haematologist, St George Hospital

Nicole Pratt (NP)
Lead statistician, UniSA

Thu-Lan Kelly (TLK)
Statistician, UniSA

Richard de Steiger (RdS)
Professor of Surgery, University of Melbourne
Deputy Director of the Australian Orthopaedic Association National Joint Replacement Registry

Anthony Harris (AH)
Health Economist

Amber Hansen
Consumer

Maggie Cripps
Consumer

Michelle Lorimer
Senior statistician, AOANJRR

Liddy Griffith
Senior Manager Clinical Research, Heart Health

Ornella Clavisi
Consumer

Qazi Sarem Shahab
Student, University of New South Wales

Emma Tsz Lou Cheng
Student, University of New South Wales

Steve Webb
Methodologist
Senior Staff Specialist, Intensive Care Medicine, Royal Perth Hospital, Clinical Professor, School of Medicine and Pharmacology & School of Population Health, University of Western Australia, Clinical Professor, Department of Epidemiology and Preventive Medicine, School of Public Health and Preventive Medicine, Monash University.

IAH conceived the study. IAH, SEG, RB, SA, JMN, IA, BHC, NP, RdS and AH are Chief Investigators on the MRFF grant. NP is the primary statistician. AH is the primary health economist. All contributors participated in protocol development.

1.4. Sponsor

South Western Sydney Clinical School, Faculty of Medicine, UNSW Sydney
Liverpool Hospital, Elizabeth St, LIVERPOOL, NSW, 2170, Australia

1.5. Study Coordination

Committees

Committee	Members	Responsibilities
Writing Committee	IAH, SG, RB, NP, SA, VS	Protocol development and publication<br>Preparation of principal publications (primary and secondary outcomes)
Steering committee	All investigators listed above (contributors)	Final protocol approval<br>Study oversight<br>Principal publication approval
Trial management committee	IAH, SEG, VS, SA, RdS, Project Coordinator (DA), AOANJRR Registry Manager, AOANJRR project manager, SAHMRI rep (LG), , SAHMRI IT, SAHMRI Data Management, SAHMRI Statistician	Integration with AOANJRR PROMs program<br>Ethics approval<br>Site liaison (recruitment and maintenance)
Data Quality Committee	IAH, Project Manager, ML, RdS	Data management<br>Data quality audits
Outcome Verification Committee	ACORN Manager, JN, IAH, BHC	Validating verification of DVT and PE reported during patient follow-up

Coordinating Centre
The day to day management of the trial will be the responsibility of the Australian Orthopaedic Association National Joint Replacement Registry (AOANJRR) and South Australian Health & Medical Research Institute (SAHMRI).

Other expert subgroups may be established throughout the project to advise on specific elements and make recommendations should the need arise.

1.6. Abbreviations

AOANJRR	Australian Orthopaedic Association National Joint Replacement Registry
AAOS	American Academy of Orthopaedic Surgeons
ACORN	Arthroplasty Clinical Outcomes Registry
DVT	Deep Venous Thrombosis
ICJME	International Committee of Medical Journal Editors
LMWH	Low Molecular Weight Heparin
NICE	National Institute of Health and Care Excellence
NOAC	Novel Oral Anticoagulant
OA	Osteoarthritis
PE	Pulmonary Embolus
PROMs	Patient Reported Outcome Measures
THA	Total Hip Arthroplasty
TKA	Total Knee Arthroplasty
HA	Hip Arthroplasty
KA	Knee Arthroplasty
VTE	Venous Thromboembolism

2. Introduction

2.1. Background

Over 100,000 total hip and total knee arthroplasty (THA, TKA) procedures are performed each year in Australia.[1] Venous thromboembolism (VTE) comprises deep venous thrombosis (DVT) and pulmonary embolus (PE) and is a recognised serious complication of hip and knee arthroplasty surgery. Patients undergoing THA and TKA receive chemoprophylaxis for VTE prevention, with most patients in Australia receiving either low molecular weight heparin (LMWH) or aspirin (manuscript in preparation).

Guideline recommendations and surgeon preference for VTE prophylaxis vary due to a lack of evidence regarding the comparative safety and effectiveness of these two common chemoprophylaxis agents. Aspirin is a low cost, over-the-counter, safe medication that is easy to take (one oral tablet daily). LMWH requires daily injection (often requiring professional or family support), is more expensive and requires prescription, but has a larger body of evidence of effectiveness. Previous studies comparing LMWH and aspirin have been underpowered for effectiveness and for safety.

Currently, practice guidelines provide conflicting recommendations for VTE prophylaxis. The National Institute of Health and Care Excellence (NICE) guidelines (United Kingdom) now (2018) recommend using LMWH, aspirin or Novel Oral Anticoagulants (NOACs) for VTE prophylaxis in TKA (aspirin is not recommended for THA) whereas aspirin was not recommended in the previous verision.[2] In the US, two main guidelines are used: those recommended by the American College of Chest Physicians (ACCP)[3] and those produced by the American Association of Orthopaedic Surgeons (AAOS).[4] Both recommend the use of LMWH, NOACs or aspirin. Previously, the ACCP guidelines recommended against aspirin whereas the AAOS guidelines recommended its use. As of 2012, both guidelines now allow the use of aspirin for VTE prophylaxis, and as a result the prevalence of aspirin prescription has increased.[5] The Australian National Health and Medical Research Council guidelines (2009) did not recommend aspirin, however these guidelines were rescinded in 2016 as they were considered outdated.[6]

A number of systematic reviews (including data from up to 22 trials) have summarised the evidence for VTE prophylaxis in joint arthroplasty, but most do not assess aspirin, despite being commonly used and recommended by some practice guidelines.[3,4,7-13]

Two small systematic reviews were found, including data from six pharmacological trials that had aspirin as a comparator.[14,15] In both reviews, the evidence was dominated by one trial of 778 patients comparing aspirin to LMWH in THA.[16] This trial was stopped early due to poor recruitment. Furthermore, all patients in the trial received LMWH for the first 10 days before random allocation to aspirin or continued LMWH. This does not reflect the way that aspirin is commonly used in Australia as aspirin is commenced during the acute care period. Another five trials were also described, including a total of 936 patients, but these trials were small, measured different outcomes, and were subject to bias.[15] Both reviews concluded that there is insufficient evidence to support recommendations on the use of

aspirin, and suggest larger trials are needed.[14,15] A recent large trial compared aspirin to rivaroxaban (a NOAC) for VTE prophylaxis in THA and TKA. A total of 3424 patients were recruited in this cluster-randomised trial, however both groups were treated with rivaroxaban for the first 5 days before being randomised to aspirin or rivaroxaban for the following 2-4 weeks.[17]

While studies using administrative datasets should be interpreted with caution due to risk of coding errors, incomplete data and difficulty fully adjusting for possible confounding, two studies of aspirin using large administrative datasets have been reported. The first, from the US, used data from 93,804 patients undergoing elective total knee replacement surgery.[18] The study compared early (30 day) mortality and VTE between patients given warfarin, LMWH and aspirin, adjusted for patient factors (age, sex, race, VTE risk, comorbidities), institution factors (size, urban/rural) and a separate propensity score. No difference was found in the mortality rates or rates of post-operative bleeding complications between the three groups, and there was no difference in the rate of VTE comparing LMWH to aspirin. A study using data from the National Joint Registry for England, Wales, Northern Ireland and the Isle of Man analysed data from 108,584 patients undergoing THA comparing LMWH to aspirin for VTE prophylaxis using multivariable modelling and propensity score matching.[19] The adjusted analysis showed no significant difference in mortality up to 90 days post-operatively but this difference became significant (favouring LMWH) on propensity score matching. There was no difference in VTE complications or re-operations (up to 90 days) between groups. The reported rates of VTE were very low, possibly due to under-detection.

The existing uncertainty regarding the relative safety and effectiveness of these different medications to prevent VTE following arthroplasty and inconsistencies in available clinical practice guidelines likely contribute to widespread clinical practice variation in Australia. A national survey[20] and recent large cohort study involving 1,900 patients from 19 institutions across Australia (manuscript under preparation) show that nearly all surgeons use some form of chemoprophylaxis, with approximately 80% using LMWH and nearly half using aspirin (approximately 40% of patients had more than one drug). The survey indicated that those using LMWH were more likely to do so for fear of litigation.[20] Aspirin does not require a prescription, is easier for patients to take (tablet rather than injection), is safe and is inexpensive. Therefore, establishing non-inferiority would provide patients with a preferred, effective, safe, cheaper and simpler method of VTE prophylaxis compared to LMWH.

The Australian Orthopaedic Association National Joint Replacement Registry (AOANJRR) was established in 1999 and reports on revision surgery and mortality after joint arthroplasty in Australia, with close to complete national coverage. The AOANJRR has established a system to directly capture data entered by patients pre- and post-operatively; this system is a platform for the conduct of clinical trials and is incorporated as part of the AOANJRR (not a standalone project). The proposed CRISTAL trial will be embedded within the Clinical Trials Platform of the AOANJRR.

2.2. Choice of comparators

Wide practice variation is evident for VTE chemoprophylaxis in Australia due to both surgeon uncertainty in the evidence for effect, and differences in surgeon preference. Aspirin and LMWH are the two most frequently used drugs for VTE prophylaxis in THA and

TKA in Australia. Alternatives, such as warfarin and unfractionated heparin are not commonly used in Australia and are not reflected in practice guidelines in this country. NOACs are an alternative to LMWH and aspirin but are not commonly used in Australia. Given aspirin's popularity, ease of use, safety profile and low cost but lack of evidence of comparative effectiveness against the most commonly recommended drug (LMWH), we considered aspirin to be the most suitable comparator to LMWH for this trial.

2.3. Study hypothesis

Aspirin is non-inferior to LMWH for prevention of VTE after THA and TKA surgery.

2.4. Primary aim

To compare the effectiveness and safety of aspirin to LMWH in preventing symptomatic VTE after primary elective THA and TKA for osteoarthritis (OA).

2.5. Secondary aims

- To compare the effectiveness and safety of aspirin to LMWH in preventing symptomatic VTE after all hip arthroplasty (HA) and knee arthroplasty (KA) including primary, revision and partial arthroplasty, performed for any indication including fracture surgery
- To compare the effectiveness and safety of aspirin to LMWH in preventing symptomatic VTE after revision HA and KA
- Compare the safety (proportion of non-VTE complications) between groups
- An extension of the primary analysis for: HA and KA separately; unilateral and bilateral separately; below-knee DVT, above-knee DVT and PE separately
- To compare the cost effectiveness of aspirin to LMWH for VTE prophylaxis after primary elective THA and TKA for osteoarthritis if aspirin is found to be inferior to LMWH

2.6. Trial design

A pragmatic multicentre, cluster-randomised, crossover, non-inferiority study with a primary endpoint of patient-reported symptomatic VTE at 90 days.

3. Methods

3.1. Setting

Eligible hospitals (public and private) performing HA and KA in Australia. The study will be nested within the AOANJRR Clinical Trials Platform, an electronic platform for recruitment and data collection for patients undergoing joint replacement surgery.

3.2. Eligibility

Hospital level

- Departmental (or surgeon group) agreement to participate in the study and adhere to study protocols
- Use of intermittent calf compression device intra-operatively and post-operatively until mobile
- Offer mobilisation day 1 post-operatively or earlier
- No change in other practices or protocols relevant to VTE over the course of the study (e.g. tourniquet use, anaesthetic type).

Patient level

- Adults (age 18 and older)
- Receiving primary or revision HA or KA for any indication (including for fracture)

Exclusion criteria

As a pragmatic study, all patients undergoing revision and primary arthroplasty for any indication during the study period will be included. The only exclusion for a department is if the volume of primary elective THA and TKA for osteoarthritis is less than 250 per year (as this may render the site unable to recruit the required sample size required for the primary analysis within a reasonable timeframe).

At an individual level, patients unsuitable to receive routine prophylaxis will be treated according to local advice and recommendations, as per normal practice. Routine prophylaxis for the purpose of the CRISTAL study includes the LMWH and the aspirin protocols used in CRISTAL. Reasons for not receiving routine prophylaxis include the long-term use of warfarin, NOAC or dual antiplatelet therapy pre-operatively, allergy to the study drug and an underlying medical condition that precludes the use of either drug or the treating doctors consider the patient to be high risk for routine prophylaxis.

3.3. Intervention

Each site will be allocated to two consecutive periods of a standard protocol of aspirin and a standard protocol of LMWH for VTE prophylaxis with the order of the two periods determined by randomisation at a 1:1 ratio, on an open label basis.

Each site will adhere to the initially randomised protocol for a time period based on surgical volume aiming for 250 patients eligible for the primary analysis per group. The target

recruitment time period for each group will be 12 months, but may extend beyond this if required. Total recruitment (all arthroplasties) to each group is expected to be 300 patients. Patients will be informed of the trial during initial data entry. Patients will be specifically asked at the time of study entry for consent for follow-up, for use of their data in research, and use of linked data to measure and verify surgical outcomes (Appendix 1). Patients will not be individually consenting to be randomised to either aspirin or LMWH, as both drugs represent standard practice and randomisation is not at the patient level. Further details on the consent process is listed in protocol (section 4.3).

Patients will be followed up at 90 (90-120) days and 6 (5-7) months, electronically, with telephone back up. To ensure minimal inconvenience a maximum of three successful reminders will be sent to the patient to complete their follow-up CRISTAL questions. Patients will be contacted beyond 100 days and 6.5 months via telephone, if surveys remain incomplete and if initial telephone contact has not been successful.

- 1st reminder
    - Pre-operative - 2 days after registration
    - 90-day post-operative – 90 days post operation
    - 6-months post-operative - 5 months + 2 weeks post operation
- 2nd reminder
    - Pre-operative - 3 days after the first reminder
    - 90-day post-operative – 95 days post-operation
    - 6-months post-operative – 2 weeks after first reminder
- 3rd reminder
    - Pre-operative - 4 days after the second reminder,
    - 90-day post-operative – 100 days after operation
    - 6-months post-operative – 2 weeks after second reminder

The electronic system used for data collection is also equipped with a Save and Complete feature which will allow an incomplete set of questions to be completed by the patient at a later date.

This was deemed necessary because if a patient is completing a set of questions and they need to stop for whatever reason it is helpful for them to be able to recommence from where they left off. For example, an issue may occur with the internet connection or, if the pre-operative data is being collected in a pre-admission clinic setting, a patient may be called away for different appointments mid-way through completing the questions.

If there is an incomplete set of questions recorded, the patient can log back in at any time within a 2-week period to complete the questions. If the questions are not completed within 48 hours of being started the system will send an additional reminder to the patient prompting them to complete the questions. After 2 weeks the session will be locked and the incomplete set of questions will be utilised.

There will be no change to usual medical follow-up (clinic attendance, investigations etc.) except that routine venous imaging is not to be used (currently not recommended and not commonly used).

Aspirin will be administered orally at 100mg (85-150 mg permitted if previously prescribed) daily for 35 (+/-7) days (hips) or 14 (+/-4) days (knees) commencing the day of or day after surgery.

LMWH will be administered as enoxaparin (Clexane) 40mg subcutaneously daily, for 35 days (hips) or 14 days (knees) commencing within 24 hours of surgery. Patients with renal impairment (creatinine clearance of <30ml/min) will be administered enoxaparin (Clexane) in a reduced dosage of 20mg subcutaneously daily and patients undergoing haemodialysis will be treated according to standard local protocol. The reduced dosage of 20 mg will also apply to patients who weigh less than 50 kilograms. Patients who have a contradiction to either study drug will be treated as per local protocols. If this requires the study drug to be withheld the study site will notify the Registry. Patients will be taught to self-administer while in hospital. For those unable to self-administer, the injections will be given by family members, a community nursing service or their local doctor, depending on local arrangements.

3.4. Adherence

Patients may discontinue the drug if they have an allergy or adverse event related to the drug.

The study drug may be withheld if post-operative wound ooze continues beyond 72 hours post-operatively, with recommencement 48 hours later if settled.

Inpatient adherence during the acute care period will be monitored by an audit of all sites over the first 2 weeks after commencing patient recruitment. A repeat audit will be performed after one month for sties that do not reach at least 80% compliance on the initial audit.

Post-discharge adherence will be determined by patient report during follow-up at 90 days.

3.5. Concomitant care

All patients will have intermittent compression devices intra-operatively and post-operatively until mobile. All patients will be offered mobilisation on day one post-surgery unless surgically or medically contraindicated.

Patients taking (non-aspirin) oral anti-platelet therapy pre-operatively may have their medication withheld for one week pre-operatively if advised by their treating doctor and will recommence their usual medication (in addition to any study medication) at day 7 post-operatively or when safe to do so.

Routine doppler screening for DVT (in asymptomatic patients) will not be permitted by participating sites.

Patients taking aspirin (85-150mg daily) pre-operatively will take this drug in the usual dose post-operatively in place of the study drug for those in the aspirin group, and in addition to

LMWH for those in the LMWH group. The aspirin may be stopped pre-operatively if advised by their treating doctors.

3.6. Primary outcome

The primary outcome is verified, symptomatic VTE (DVT or PE) at 90-day follow-up.
All reports of VTE will be verified by contact with treating doctors and institutions. VTE will be deemed verified by the independent Outcome Verification Committee. The absence of VTE will be verified in a random sample of 200 patients reporting an absence of VTE, by auditing treating doctors and institutions. Patients will be asked if they are still taking anticoagulant medication at 90-day follow-up. VTEs will be subclassified into all DVT, below knee DVT, above knee DVT and PE.

3.7. Secondary outcomes

1. Non-VTE complications (see below)
2. PROMs. Health-related quality of life (EQ5D-5L), Oxford hip and knee scores and patient-rated satisfaction and improvement.
3. Costs. If aspirin is found to be inferior to LMWH, the cost of anticoagulation, cost of hospital stay, need for further health resource utilisation (e.g. nurse or GP visits) and complications will be analysed (see list below)
4. Adherence. Proportion of patients taking the drug continuously (no more than 2 consecutive days missed) for the recommended minimum period.

Non-VTE Complications will be classified into the following groups by the Outcome Verification Committee:

- Readmission related to the original surgery or associated treatment (including bleeding and VTE related) within 90 days
- Reason for readmission (infection, dislocation, stiffness, fracture, wound dehiscence, implant loosening, migration or failure, wound bleeding, other bleeding) within 90 days
- Major bleeding events within 90 days (‘major’ defined as those resulting in readmission , reoperation or death)
- Reoperation on the same joint within 90 days and 6 months
- Type of reoperation (treatment of infection, reduction of dislocation, manipulation under anaesthesia, fracture treatment, wound repair, implant loosening, migration or failure, non-joint related surgery) within 90 days and 6 months
- Death within 90 days and 6 months

All reports of non-VTE complications will be verified by contact with treating doctors and institutions, except for death, which will be verified through the National Death Index (NDI).

3.8. Participant timeline

Time Point	Data Collection Questions and Instruments
Pre-operative	Current anticoagulation use (yes/no and drug)<br>History of previous VTE<br>Age<br>Sex<br>Joint (hip or knee)<br>Side<br>Unilateral vs bilateral<br>Primary or revision<br>ASA grade<br>BMI<br>Oxford Hip or Knee Score<br>EQ-5D-5L<br>EQVAS<br>Low back pain<br>Joint pain (numeric rating scale 0-10)<br>Expectations (pain and improvement)
90 days	VTE (DVT or PE)<br>Adherence (did you use pills or injections to prevent a blood clot post-operatively, for how long?)<br>Current use of anticoagulants (yes/no, which one)<br>Complications (asked individually, as per complication list)
6 months	Complications (asked individually, as per complication list)<br>Oxford Hip or Knee Score<br>EQ-5D-5L<br>Joint pain (scale 0-10)<br>Satisfaction with outcome of surgery<br>Patient-rated improvement

<u>EQ-5D-5L (Appendix 2)</u>
The EQ-5D is a standardised measure of health status developed by the EuroQol Group in order to provide a simple, generic measure of health for clinical and economic appraisal.[21] The survey includes 5 health outcome domains that can be summarised into a utility score. These include:

- Mobility
- Self-care
- Usual Activities
- Pain/Discomfort
- Anxiety/Depression

There are five descriptive sentences under each heading and patients are directed to tick one box that best describes their health on that day. There is also a visual analogue scale (VAS) that addresses health state.[21]

Oxford Hip or Knee Scores (Appendix 3)
The Oxford hip (OHS) and knee (OKS) scores were developed in the mid-1990s. The scores were developed to assess the outcome of hip and knee replacements as well as shoulder surgery (including shoulder replacement) and were designed to be completed by patients in order to minimise potential bias.[22] Both two instruments include 12 questions to assess a patient's capacity to undertake general activities of daily living, about their affected hip or knee.

Pre-operative anticoagulation use (Appendix 4)
Questions listed below will be presented pre-operatively to determine any history of VTE and current use of Anti-coagulant medications. The questions were reviewed and approved by consumer representatives and will be specific to the medications and the site of DVT.

1. Do you normally take (or are you currently taking) blood thinning medication routinely?
2. Do you know what blood thinning medication you are taking?
3. Have you ever been diagnosed with a clot in your legs (Deep Vein Thrombosis - DVT) or lungs (Pulmonary Embolism - PE)?
4. When did your most recent (or only) clot in your legs (Deep Vein Thrombosis - DVT) or lungs (Pulmonary Embolism - PE) occur?

Post-operative VTE symptoms and occurrence (Appendix 4)
The following questions will be presented post-operatively at both 90 days and 6 months to gauge patient's surgery outcome. Questions 1 to 5 are only specific for the 90 days data collection point and will not be asked on the 6 months data collection point.

1. Did you take your post-surgery blood thinning medication (to avoid blood clots) after leaving hospital following your joint replacement operation?
2. Do you know what blood thinning medication you were taking?
3. How many days did you take the blood thinning medication after your (HIP/KNEE) replacement operation?
4. Since your joint replacement surgery, have you been diagnosed with a blood clot in your legs (Deep Vein Thrombosis - DVT) or lungs (Pulmonary Embolism - PE)?
5. Since your joint replacement surgery, have you had any serious bleeding from anywhere in your body not related to your joint replacement?
6. Have you had any further surgery on your replaced joint (apart from when it was put in)?
7. Please select the reason(s) for the additional surgery/surgeries (select all that apply)

3.9. Sample size

A recent large cohort study of 1900 THA and TKA patients from 19 institutions across Australia showed an incidence of symptomatic VTE within 90 days of THA and TKA of 2.6% (manuscript under preparation). A recent randomised trial of aspirin versus rivaroxaban used a minimum clinically important difference of 1%, based on a survey of thromboembolism experts and orthopaedic surgeons.[17]

For the sample size calculation in the CRISTAL study, we used an estimated overall event rate of 2% (a conservative estimate based on the recent Australian cohort study and the current available literature) [15-17,19,23-26], the same non-inferiority margin of 1% from the recent randomised controlled trial (for aspirin compared to LMWH, 2.5% for aspirin and 1.5% for LMWH)[17], a power of 90% and a one-sided significance level of 0.025. For an individual randomised trial, this yields a sample size of 4,117 per treatment group or a total of 8,234 patients. For a cluster randomised crossover trial, the sample size must account for correlations within clusters during the same time period (intracluster correlation) and between study periods in the same cluster (interperiod correlation).[27,28] Assuming an intracluster correlation of 0.01, an interperiod correlation of 0.008 and 31 clusters, the sample size required increases to 11,160 patients. From each cluster, we will aim to recruit minimum of 251 registered patients from each group (a total of 15,562 patients), which will allow a 27% loss to follow-up.

Due to uncertainty around the exact event rate[15-17,19,23,24] and to allow for a smaller non-inferiority margin, we have constructed a sample-size table (Table 1) to demonstrate that the trial will be adequately powered using a non-inferiority margin of 1%, for an event rate up to 3% at 80% power and for an event rate up to 2% at 90% power, provided that loss to follow-up is less than 17%. As a secondary measure, after 1,000 patients have completed the 90-day follow-up, we will obtain a preliminary symptomatic VTE rate for the whole sample and a loss to follow-up rate (without performing any comparative statistical analyses and maintaining blinding) to determine whether the estimates for the primary event rate (2%) and loss to follow-up rate (27%) are accurate and adjust the sample size accordingly if the primary event rate is greater than 3%, whilst accounting for loss to follow-up.

Table 1 – Sample Size Table for the CRISTAL Trial † ‡

Event rate in experimental	Event rate in control	Overall event rate	Non inferiority margin	N in each group (individual)	Cluster size (for 31 clusters)	N total (cluster randomised)
**Power = 0.8**						
0.015	0.005	0.01	0.01	1553	56	3472
0.02	0.01	0.015	0.01	2319	88	5456
0.025	0.015	0.02	0.01	3076	123	7626
0.03	0.02	0.025	0.01	3826	163	10106
0.035	0.025	0.03	0.01	4567	207	12834
0.04	0.03	0.035	0.01	5301	258	15996
0.0125	0.005	0.00875	0.0075	2420	92	5704
0.015	0.0075	0.01125	0.0075	3104	124	7688
0.0175	0.01	0.01375	0.0075	3784	160	9920
0.02	0.0125	0.01625	0.0075	4461	201	12462
0.0225	0.015	0.01875	0.0075	5134	246	15252
**Power = 0.9**						
0.015	0.005	0.01	0.01	2079	77	4774
0.02	0.01	0.015	0.01	3103	124	7688
0.025	0.015	0.02	0.01	4117	180	11160
0.03	0.02	0.025	0.01	5121	245	15190
0.015	0.0075	0.01125	0.0075	4154	182	11284
0.0175	0.01	0.01375	0.0075	5065	241	14942

† A one sided $\alpha = 0.025$ is required for a 95% CI. The number of clusters is assumed to 31, the ICC = 0.01 and the IPC=0.008.
‡ Table does not account for an estimation of loss to follow-up

3.10. Recruitment

Hospitals will be approached individually by the lead CI and the study team, as appropriate. A site will be considered eligible if they can recruit 251 eligible patients (for the primary analysis for each group) with an aim to recruit this number within 12 months. Departmental (or surgeon group) agreement with the study protocol and the individual treatment protocols (for each group) will be required. Sites where a subgroup of attending surgeons agree to participate will be included if the number of eligible patients for that group of surgeons per year it at least 250. A site investigator will be nominated for each site

3.11. Randomisation

Each site will be randomised with a 1:1 allocation with a computer generated random sequence. Simple randomisation will be used (no use of blocks, no stratification). The allocation will refer to the first intervention.

3.12. Blinding

Sites will not be blinded to group allocation. Patients will be aware of a study comparing different treatments for VTE prevention but will not know the specific details of the study and will therefore be blind to the specific interventions and outcomes of the trial such as whether they are in the intervention or control group and the secondary outcomes of the trial. Outcomes will be self-reported with verification of the primary outcome by the Outcome Verification Committee. Where outcome reporting is by phone (back up for failure to capture patients reported outcomes electronically and where verification is performed), those outcome assessors will be blinded to group allocation.

The Outcome Verification Committee will be presented with deidentified cases for assessment. The statistical analysis will be blinded. The Writing Committee will be blinded and will prepare separate manuscripts based on the possible group allocations.

3.13. Data collection

Data collection for baseline data and follow-up at 90 days and 6 months will be patient-reported electronically (via tablet, phone or computer) using direct data entry. For patients not responding to email and SMS follow-up, telephone contact will be used to administer the surveys verbally.

Pre-Operative

When collected electronically by patients, data will be directly entered into the AOANJRR Clinical Trials Platform.

Some patients may not have direct access to the internet. This is especially a factor for older patients and patients from lower socioeconomic groups.[29-31] Another group who could potentially be excluded from implementing this approach to data collection are non-English speaking patients. To overcome these barriers patients will be given the option to nominate a 'proxy' e.g. family member or friend to assist them with completing the instruments and receive reminders electronically on their behalf. Information will be collected on whether the patient had assistance to complete the CRISTAL questions and these data will be reviewed during the analysis.

There will be various methods and procedures implemented at the different hospitals to register patients and request them to complete the CRISTAL data online. Ideally the patients will complete pre-operative CRISTAL data immediately when first approached, however, this may not always be possible. There is functionality built into the system to email a patient the link to the website to complete the CRISTAL requirements at a time that is convenient for them.

Some hospitals participating in CRISTAL already routinely collect PROMs and wish to continue to do so using their own systems. In these cases, the AOANJRR will work with the hospital to simplify the data collection process and avoid duplication of collection. A data sharing agreement will be entered into between the hospital and the AOANJRR whereby data can be exported from the current system and imported into the AOANJRR Clinical Trials Platform. There will be a secure file sharing facility established within the web application to

ensure secure transfer of confidential information. The data provided will be reviewed by the data manager prior to upload into the database to confirm quality and completeness.

Post-Operative
Follow-up will be by telephone until the electronic data capture system is built and telephone follow-up will be used as back up for the electronic follow-up once in place. Patients will be able to login and complete their 90-day follow-up from 75 days and 6-month follow-up from 5 months.

The Arthroplasty Clinical Outcomes Registry (ACORN) has been contracted to complete the follow-up phone calls. ACORN was selected because this Registry already collects PROMs centrally for hospitals, predominately in NSW, and the staff have expertise in this area.

3.14. Data management

Data quality will be checked monthly under the supervision of the Data Quality Committee.

3.15. Statistical analysis

The analysis for the primary objective will be limited to patients undergoing elective primary THA or TKA for a diagnosis of OA, excluding patients for whom the study drugs were contraindicated (e.g., allergy or need for alternative anticoagulant – warfarin, NOAC, dual antiplatelet, for a pre-existing condition). This analysis will test between-group difference in the proportion of cases developing VTE within 90 days for non-inferiority of aspirin at a margin of 1%, on an intention to treat basis.

The primary analysis will use cluster summary methods.[32] These methods estimate the treatment effect using cluster level differences and have been shown to be appropriate for cluster randomised crossover trials with rare outcomes and the intracluster and interperiod correlation coefficients expected in this trial.[33]

Multiple imputation to account for missing outcome data will be investigated, using auxiliary variables gathered from routine AOANJRR data (including age, sex, baseline health, pain and function, diagnosis and surgical factors). Since VTE is rare, if prediction in the imputation models using these auxiliary variables is a problem, no imputation will be performed due to the possibility of bias.[34] Since the most likely reason for loss to follow-up is difficulty in contacting patients postoperatively (rather than any association with treatment assignment or outcome), missing outcome data is expected to be missing completely at random, which will not cause bias in the estimates.

Secondary analyses will be performed for the primary outcome, to test for differences in treatment effect between subgroups of patients: THA only, TKA only and bilateral joint replacement. The analysis method will be the same as the primary outcome and will include an interaction term between subgroup and treatment group.

Further secondary analyses will include an extension of the primary analysis for patients undergoing all forms of HA and KA (total, revision, partial) for any indication (non-elective surgery, non-OA diagnoses) and will include patients for whom the study drug was

contraindicated. This will assess the effect of implementing the protocol at a departmental (hospital) level. Other secondary analyses will include an analysis of the subcategories of VTE as the outcome; PE only, all DVT, above knee DVT only and below knee DVT only and non-VTE related complications (death, re-operation, major bleeding and readmission rates). Cluster summary methods will be used for all secondary analyses.

If aspirin is found to be inferior to LMWH, a cost effectiveness analysis of aspirin compared to LMWH will be performed from a health system perspective. Data for resource use associated with treatments and complications will be taken from trial data within the AOANJRR. Survival at one year and quality of life measured using EQ5D at baseline and six months will allow calculation of differences in quality adjusted life years (QALYs) between groups. We will calculate the cost per QALY for each treatment comparison as the difference in mean costs divided by the difference in mean outcomes (quality adjusted survival as QALYs) over the duration of the trial, using regression analysis to adjust for differences at baseline and clustering by site.

3.16. Data monitoring and cleaning

A separate Data Quality Committee will be established to monitor data management and quality.

A separate safety monitoring committee will not be established and no stopping rules will be used as both interventions are commonly used and recommended treatments. No interim analysis will be performed; this will reduce the chance of early stopping due to spurious findings. Adverse events (separate to complications listed under secondary outcomes) will be monitored by the Trial Management Committee).

3.17. Auditing and Data validation

Positive outcomes (reported VTE) reported by patients will be confirmed by contacting hospitals or treating doctors. These will be adjudicated by the Outcome Verification Committee.

A sample of negative outcomes (patients reporting the absence of VTE) will be verified in a similar manner in a random sample of 200 patients with negative outcomes.
Complications will be adjudicated by the Outcome Verification Committee.
ANZMUSC will audit the study.

The AOANJRR Data Linkage project will be used to test the accuracy of outcome reporting (readmission, re-operation, drug prescriptions). The AOANJRR also links to the National Death Index (NDI) twice a year (February and September). If a patient, who has participated in the CRISTAL project, is flagged as deceased in the AOANJRR database this can also be transferred to the CRISTAL system and no further contact with be made, reducing distress for families.

4. Ethics and dissemination

4.1. Ethics approval

The study will be submitted to Sydney Local Health District (RPAH Zone) human research ethics committee for approval. Following approval, the study will be submitted to local ethics committees and Research Governance Offices as required for each site. Refer to the Table 2 for sites.

Table 2: List of Sites for Ethics approval by Sydney Local Health District (RPAH Zone)

State	Hospital	Comment
NSW	Canterbury Hospital	
NSW	Coffs Harbour	
NSW	Fairfield Hospital	
NSW	Gosford Public Hospital	
NSW	Hornsby Ku-ring-gai Hospital	
NSW	Kareena Private Hospital	Private Hospital (notified that an EEA is in place)
NSW	Nepean Hospital	
NSW	North Shore Private Hospital	Private Hospital (notified that an EEA is in place)
NSW	Prince of Wales Hospital	
NSW	Royal Prince Alfred Hospital (Institute of Rheumatology and Orthopaedic Surgery)	
NSW	Royal North Shore Hospital	
NSW	Ryde Hospital	
NSW	St George Private Hospital & Medical Centre	Private Hospital (notified that an EEA is in place)
NSW	Sutherland Hospital	
NSW	Westmead Private Hospital	Private Hospital (notified that an EEA is in place)
QLD	Greenslopes Private Hospital	Private Hospital (notified that an EEA is in place)
QLD	Mater Adults Hospital	
QLD	Prince Charles Hospital	
SA	Flinders Medical Centre	
VIC	Bendigo Hospital	
VIC	Epworth Private Hospital	
VIC	Frankston Hospital	

VIC	Hollywood Private Hospital	Private Hospital (notified that an EEA is in place)
VIC	University Hospital Geelong Barwon Health	
VIC	Warringal Hospital	Private Hospital (notified that an EEA is in place)
VIC	Western Hospital Footscray	
VIC	Western Hospital Williamstown	
WA	Fremantle Hospital	
WA	Osborne Park Hospital	
WA	Royal Perth Hospital	
WA	Sir Charles Gairdner Hospital	

4.2. Amendments

Any modifications to the protocol which may impact on the conduct of the study, potential benefit of the patient or may affect patient safety, including changes of study objectives, study design, patient population, sample sizes, study procedures, or significant administrative aspects will require a formal amendment to the protocol. Such amendments will be agreed upon by the Steering Committee and approved by the Ethics Committee prior to implementation and site notification.

Administrative changes of the protocol are minor corrections and/or clarifications that have no effect on the way the study is to be conducted. These administrative changes will be agreed upon by Trial Management Committee and will be documented in a memorandum. The Ethics Committee/IRB may be notified of administrative changes at the discretion of Trial Management Committee.

4.3. Consent to project participation

Individual consent is not being sought for randomisation or use of the study drugs. This is because randomisation is not occurring at the patient level and because both study drugs represent current standard practice. Consent is being sought for the collection and use of patient data, as per standard protocol for the AOANJRR Clinical Trials Platform. The Clinical Trials Platform uses the same consent process (and near identical data collection) as the AOANJRR PROMS Pilot Project which has received ethics approval (Reference: X18-0057 & HREC/18/RPAH/90).

<u>Consent to AOANJRR Clinical Trials Platform</u>
Consent will be obtained electronically. All data collection for this project is electronic. This provides efficiency and effectiveness (less error) and allows a better way to impart information relevant to the consent process. The participant information and consent form will be displayed on the screen. It contains all elements required for a consent form (see Appendix 1). The information under each statement will be expandable. Patients will be provided the option to ‘agree to the statement’ or ‘learn more’. If the patient agrees they will be navigated to the next statement. If the patient chooses to learn more the additional information will be displayed. Once all statements have been agreed to the patient will be

able to choose whether they give consent or no longer wish to participate in the study. If the patient consents to participate they will be directed to the next page where they can complete the required pre-operative CRISTAL questions relevant to their procedure. If the patient chooses not to consent after the initial registration, then all personal information collected at registration will be deleted from the database. The only data that will be retained is:

- Hospital Name (if available)
- Surgeon Name (if available)
- Date of registration

If the patient elects to withdraw at the time of their post-operative assessment, then no further follow-up will be undertaken.

<u>Electronic Consent Process Flowchart</u>

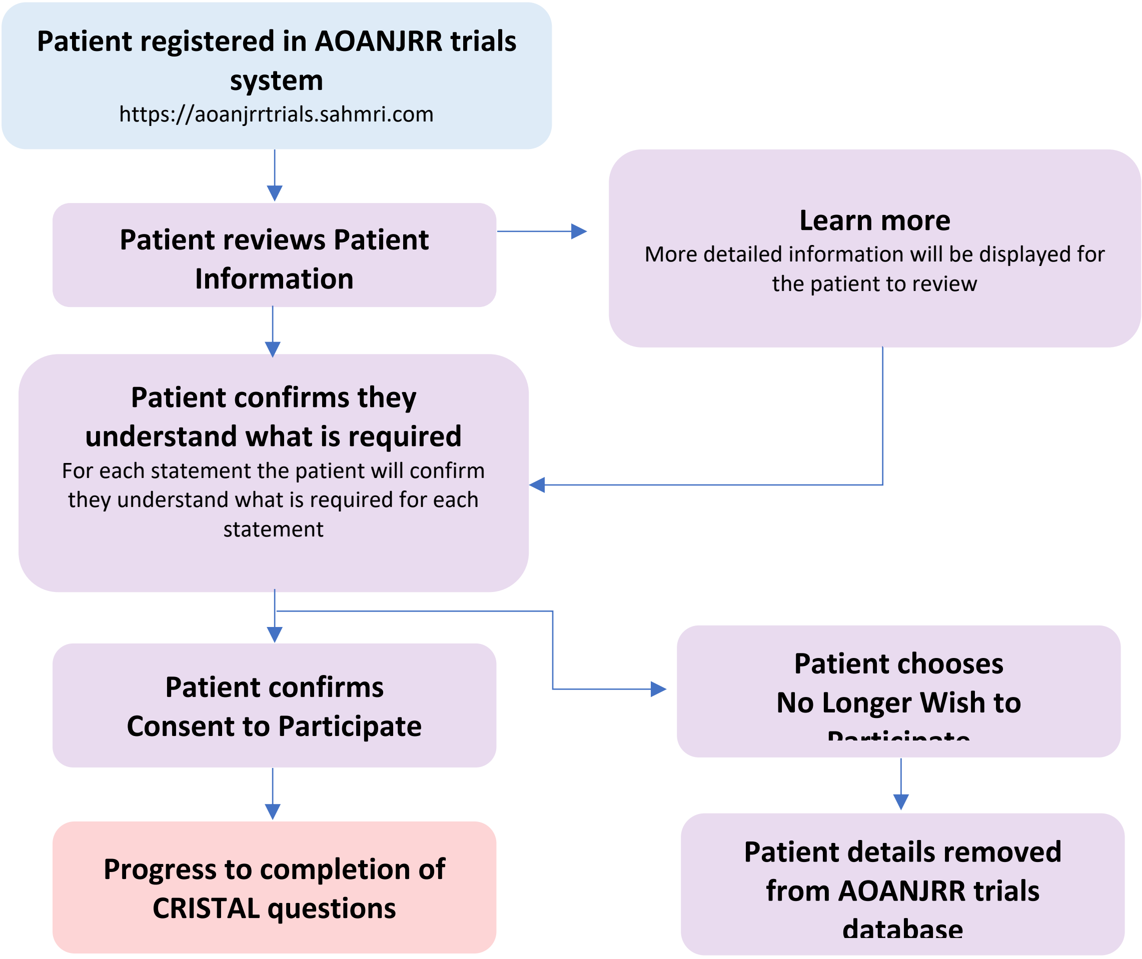

<u>Consent to share data</u>
After completing the pre- and post-operative CRISTAL questions, patients will be given the option (at each time point) to share their data with their treating surgeon. If the patient consents for their results to be shared the treating surgeon will be able to download patient level data from the CRISTAL system. If patients do not consent to share these data, their information will not be provided to the surgeon.

Waiver of Consent

We are requesting waiver of consent for two different aspects of this project. Firstly, we require waiver of consent for the ‘registration’ process which involves getting basic contact information from potential participants so that they can be contacted and formally invited to participate and consent. An identical process of registration has been previously approved for the AOANJRR PROMs pilot. Secondly, we request waiver of consent for randomisation and treatment with the study drugs (the intervention) that make up the CRISTAL project.

Each request for waiver of consent are listed below: firstly, for the ‘registration’ process (clinical trials platform), and secondly for specific involvement (randomisation and treatment) in the CRISTAL study.

Waiver of consent Part 1: patient registration prior to initial patient contact.

The request for waiver of consent only applies in some instances. Specifically, some patients will be registered in the system by hospital administrative staff or their treating surgeon. Once this registration occurs the patient will subsequently be sent an email by the AOANJRR to obtain consent electronically prior to completing the CRISTAL questions. The data that will be stored within the AOANJRR between registration and consent includes:

- Patient First Name
- Patient Middle Name
- Patient Surname
- Date of Birth
- Postcode
- Hospital
- Surgeon name
- The joint that will be operated on (Hip, Knee)
- The side that will be operated on (Left, Right, Both)
- Patient contact details such as phone number and email address

It is important to emphasise that the AOANJRR will collect almost all the registration information when it is provided at the time of surgery except for phone number and email address. We believe this request satisfies the criteria as detailed in the National Statement on Ethical Conduct in Human Research (2013, chapter 2.3) for providing a waiver of consent. This waiver of consent is only for collecting information through the registration process and not to the completion of the CRISTAL instruments.

a. Involvement in the research carries no more than low risk

    - This is a low risk project particularly as the waiver is specifically required to defer involvement in the project after the registration as a mechanism to ensure that patients that take this option can complete their involvement in the project at a time that is most suitable to them.

b. the benefits from the research justify any risks of harm associated with not seeking consent

   - There is no risk of harm associated with storing the patient's details prior to collecting consent. The Registry will receive almost all of this data at the time of the procedure. It is being requested to enhance participant convenience. The AOANJRR is a declared Federal Quality Assurance Activity and all data is managed in accordance with that declaration which includes the use of high level security systems.

c. it is impracticable to obtain consent (for example, due to the quantity, age or accessibility of records)

   - It is not feasible to collect patient consent prior to the registration as it is necessary to link the electronic consent to the individual patient identified by the registration process. If the consent is completed prior to registration then the consent will be unidentified.

d. there is no known or likely reason for thinking that participants would not have consented if they had been asked

   - Patients will verbally consent to have their details recorded at registration and this will be subsequently confirmed prior to completion of the CRISTAL Questions instruments. It is the AOANJRR experience that very few patients are reluctant to have their data included in the Registry.

e. there is sufficient protection of their privacy

   - The AOANJRR is a declared Federal Quality Assurance Activity
   - Systems are in place to ensure individual patient data remains confidential
   - A third-party security review and penetration testing was undertaken prior to commencement of data collection in the clinical trials system.

f. there is an adequate plan to protect the confidentiality of data

   - SAHMRI, which is the organisation responsible for managing AOANJRR data, has existing security systems, policies and procedures in place as well as software barriers to protect personal information and ensure confidentiality. These systems are already in place for data contained within the AOANJRR and the CRISTAL data will be treated identically (see Appendix 5).

g. in case the results have significance for the participants' welfare there is, where practicable, a plan for making information arising from the research available to them (for example, via a disease-specific website or regional news media)

   - Patients will be able to review their own results and how they compare to the national average via online dashboards.

h. the possibility of commercial exploitation of derivatives of the data or tissue will not deprive the participants of any financial benefits to which they would be entitled

    - The AOANJRR is a not for profit organisation which does not use the data it collects for commercial gain.

i. the waiver is not prohibited by State, federal, or international law

    - There are no applicable laws prohibiting this waiver.

<u>Waiver of consent Part 2: participant randomisation and treatment under the CRISTAL study.</u>

The CRISTAL study is seeking a waiver of individual consent for the intervention proposed by the study (the administration of either aspirin or LMWH). It is recommended that all patients who undergo THA or TKA require chemoprophylaxis to prevent VTE, with-holding or not giving chemoprophylaxis is considered against the current standard of care. We believe this request satisfies the criteria as detailed in the National Statement on Ethical Conduct in Human Research (2013, chapter 2.3) for providing a waiver of consent.

a. involvement in the research carries no more than low risk

    - This is a low risk project as it involves interventions that are currently used in standard practice (aspirin and LMWH for VTE prophylaxis). The additional questions asked of patients is not considered of sufficient risk or burden to justify specific consent as they are also part of routine practice (follow-up health and complication questionnaires) for most sites. Furthermore, randomisation is not at the patient level – it occurs at the site level (cluster randomisation).

b. the benefits from the research justify any risks of harm associated with not seeking consent

    - There is no additional harm from this study as both intervention arms are standard practice. There may be an imbalance of harms between groups, but this study is necessary to determine this, and this highlights the benefits that will arise from the research as there is currently insufficient evidence to guide practice which has resulted in widespread practice variation.

c. it is impracticable to obtain consent (for example, due to the quantity, age or accessibility of records)

    - Specific consent for CRISTAL would require an additional consent process (in addition to the consent for the use of data). This would make entry into the research cumbersome and confusing and would likely lead to a higher proportion of patients abandoning data entry, reducing the scientific validity of the study.

d. there is no known or likely reason for thinking that participants would not have consented if they had been asked

    - Patients currently receive VTE prophylaxis without consent as part of standard practice and we consider the process of this trial to be similar to standard practice.

e. there is sufficient protection of their privacy

    - The AOANJRR is a declared Federal Quality Assurance Activity
    - Systems are in place to ensure individual patient data remains confidential
    - A third-party security review and penetration testing has been undertaken prior to commencement of data collection in the clinical trials system.

f. there is an adequate plan to protect the confidentiality of data

    - SAHMRI which is the organisation responsible for managing AOANJRR data has existing security systems, policies and procedures in place as well as software barriers to protect personal information and ensure confidentiality. These systems are already in place for data contained within the AOANJRR Clinical Trials platform and the CRISTAL data will be treated identically (see Appendix 5)

g. in case the results have significance for the participants' welfare there is, where practicable, a plan for making information arising from the research available to them (for example, via a disease-specific website or regional news media)

    - It is the intent of the researchers that the results of the CRISTAL study will be synthesised and published as a clinical trial in a peer reviewed journal. The only trial data of relevance to the patients will be the development of adverse events or VTE, which will be known to them at the time. Other data collected as part of the Registry will be made available to patients as per usual practice.

h. the possibility of commercial exploitation of derivatives of the data or tissue will not deprive the participants of any financial benefits to which they would be entitled

    - The CRISTAL study is receiving no industry or pharmaceutical corporation support and does not aim to make any financial profit or gain during the trial or after publication of the results. It will not deprive participants of any financial benefits.

i. the waiver is not prohibited by State, federal, or international law

    - There are no applicable laws prohibiting this waiver.

4.4. Confidentiality

AOANJRR is required to have highly secure data protection systems to secure the identified information which it currently holds as this is an absolute requirement under its Federal Quality Assurance Activity.
SAHMRI has been contracted to build the AOANJRR Trials which will be utilised for CRISTAL. SAHMRI has existing security systems, policies and procedures in place as well as software barriers to protect personal information and ensure confidentiality. (Appendix 5)

As this will be a fully electronic system, accessible online, which will store patient's personal and contact details, additional security activities have been included in the AOANJRR Trials system development:

- A third-party security review of the infrastructure and application design was undertaken, prior to development starting
- A penetration test of the application was performed prior to commencement of data collection in the clinical trials system.

Patient Confidentiality
All patient data will be managed in accordance with the Guidelines for the Protection of Privacy in the Conduct of Medical Research. Patient contact details will only be used for the purpose for which they were collected and will be stored securely and confidentially. Patients will not be identified in any reports, manuscripts or presentations derived from the CRISTAL project.

Surgeon Confidentiality
No individual surgeons will be identified in any reports or manuscripts.

4.5. Risk to Patients

As patients will be treated with the standard protocol for both LWMH and Aspirin, this study poses no foreseeable risk, harm or discomfort to patients beyond the inconvenience associated with completing the study questionnaires at three-time points. We recognise the burden of survey completion but also recognise that patient outcome collection is now becoming a standard part of patient care and will be standard practice in most sites recruiting for CRISTAL.

As patient follow-up is a requirement of this project all efforts will be made not to contact the relatives of a deceased participant. The AOANJRR links to the National Death Index (NDI) twice a year (February and September). If a patient, who has participated in the CRISTAL project, is flagged as deceased this will be transferred to the AOANJRR trials when the procedure date is linked. This will stop any automated and manual reminders being triggered. Notification of all deaths to the respective HRECs will occur biannually following the linking of the AOANJRR core date to the NDI. NDI matching provides 'fact of death' data only and no causality is determined.

4.6. Safety Monitoring and Management of Serious Adverse Events

The principal investigators will be responsible of notifying the AOANJRR of any known serious adverse event that occurred at their respective site. The event will then be reviewed by the Trial Management Committee to determine if it warrants a review by the Data Safety Monitoring Board (DSMB).

The DSMB will also be notified as soon as practicable by the relevant principle investigators of any VTE related participant deaths as they become aware of the events. This includes post-surgical inpatient deaths or deaths after discharge of which the Principle Investigator or researcher becomes aware.

A DSMB was established after the commencement of the study. The DSMB consists of one orthopaedic surgeon, one haematologist and one statistician. All members are independent to the study and will review serious adverse events when deemed necessary by the Trial Management Committee. DSMB recommendations will be reviewed by the Trial Management Committee for their approval.

4.7. Declaration of interests

Ian Harris (IH), Stephen Graves (SG), Richard de Steiger (Rds) and Michelle Lorimer are employed by the AOANJRR. Nicole Pratt (NP)'s salary is partly supported by the MRFF grant received for CRISTAL.

4.8. Data access

All principal investigators involved in data analysis will have access to deidentified datasets. All principal investigators involved in subcommittees will have access to relevant deidentified data necessary for undertaking their specific role (e.g. outcome validation).

4.9. Additional care

As both interventions are standard, recommended practice, no additional treatment will be provided for participants.

4.10. Dissemination

A writing committee will be established to write the principal papers (primary and secondary outcomes). Dissemination will be by peer reviewed journal publication, conference presentation and through media. All study findings will be reported, regardless of statistical significance or the size or direction of effect.

Study findings will be released to participating sites and investigators.

Input will be sought into guideline development by state and national bodies (e.g. ACSQHC). The results of the study are expected to be published in a journal with high impact and to be of interest to a wide audience (beyond orthopaedics and haematology, including hospitalists

and public health). They are expected to have clinical importance and statistical power that will enable the results to influence practice, which currently lacks studies on this size and quality.

4.11. Implementation

Surgeons will be surveyed prior to commencement (separate study) to assess their willingness to change practice based on the results of the trial, allowing for current practice, study findings, experience, gender.

Surgeons will be asked to sign a commitment to change. Following the study, practice change at departmental and surgeon level will be measured for each surgeon at each site by assessing departmental and individual surgeon prophylaxis methods.

Practice change more broadly will be assessed through data linkage, assessing the increase or decrease in post-operative LMWH prescriptions.

4.12. Authorship

Authorship for principal papers will be by the members of the writing committee and the CRISTAL Study Group (consisting of all investigators according to the authorship guidelines of the ICMJE).

5. Statement for compliance with NHMRC National Statement on Ethical Conduct of Research Involving Humans

This study will be conducted in accordance with the ethical principles that have their origin from the Declaration of Helsinki and are consistent with ICH/GCP. This study will comply with National Health and Medical Research Council (NHMRC) National Statement on Ethical Conduct in Research Involving Humans.

6. References

1. AOA. Australian Orthopaedic Association National Joint Replacement Registry Annual Report. 2017.
2. NICE. Venous thromboembolism in over 16s: reducing the risk of hospital-acquired deep vein thrombosis or pulmonary embolism. 2018.
3. Falck-Ytter Y, Francis CW, Johanson NA, et al. Prevention of VTE in orthopedic surgery patients: Antithrombotic Therapy and Prevention of Thrombosis, 9th ed: American College of Chest Physicians Evidence-Based Clinical Practice Guidelines. *Chest* 2012; **141**(2 Suppl): e278S-e325S.
4. Mont MA, Jacobs JJ, Boggio LN, et al. Preventing venous thromboembolic disease in patients undergoing elective hip and knee arthroplasty. *J Am Acad Orthop Surg* 2011; **19**(12): 768-76.
5. Shah SS, Satin AM, Mullen JR, Merwin S, Goldin M, Sgaglione NA. Impact of recent guideline changes on aspirin prescribing after knee arthroplasty. *J Orthop Surg Res* 2016; **11**(1): 123.
6. NHMRC. National Health and Medical Research Council. Clinical Practice Guideline for the Prevention of Venous Thromboembolism - Rescinded. 2009.
7. Adam SS, McDuffie JR, Lachiewicz PF, Ortel TL, Williams JW, Jr. Comparative effectiveness of new oral anticoagulants and standard thromboprophylaxis in patients having total hip or knee replacement: a systematic review. *Ann Intern Med* 2013; **159**(4): 275-84.
8. Cao YB, Zhang JD, Shen H, Jiang YY. Rivaroxaban versus enoxaparin for thromboprophylaxis after total hip or knee arthroplasty: a meta-analysis of randomized controlled trials. *Eur J Clin Pharmacol* 2010; **66**(11): 1099-108.
9. Cohen A, Drost P, Marchant N, et al. The efficacy and safety of pharmacological prophylaxis of venous thromboembolism following elective knee or hip replacement: systematic review and network meta-analysis. *Clin Appl Thromb Hemost* 2012; **18**(6): 611-27.
10. Gomez-Outes A, Terleira-Fernandez AI, Suarez-Gea ML, Vargas-Castrillon E. Dabigatran, rivaroxaban, or apixaban versus enoxaparin for thromboprophylaxis after total hip or knee replacement: systematic review, meta-analysis, and indirect treatment comparisons. *BMJ* 2012; **344**: e3675.
11. Harenberg J, Marx S, Dahl OE, et al. Interpretation of endpoints in a network meta-analysis of new oral anticoagulants following total hip or total knee replacement surgery. *Thromb Haemost* 2012; **108**(5): 903-12.
12. Neumann I, Rada G, Claro JC, et al. Oral direct Factor Xa inhibitors versus low-molecular-weight heparin to prevent venous thromboembolism in patients undergoing total hip or knee replacement: a systematic review and meta-analysis. *Ann Intern Med* 2012; **156**(10): 710-9.
13. Turun S, Banghua L, Yuan Y, Zhenhui L, Ying N, Jin C. A systematic review of rivaroxaban versus enoxaparin in the prevention of venous thromboembolism after hip or knee replacement. *Thromb Res* 2011; **127**(6): 525-34.
14. Drescher FS, Sirovich BE, Lee A, Morrison DH, Chiang WH, Larson RJ. Aspirin versus anticoagulation for prevention of venous thromboembolism major lower extremity orthopedic surgery: a systematic review and meta-analysis. *J Hosp Med* 2014; **9**(9): 579-85.

15. Wilson DG, Poole WE, Chauhan SK, Rogers BA. Systematic review of aspirin for thromboprophylaxis in modern elective total hip and knee arthroplasty. *Bone Joint J* 2016; **98-B**(8): 1056-61.
16. Anderson DR, Dunbar MJ, Kahn SR. Aspirin versus low-molecular-weight heparin after total hip arthroplasty. *Ann Intern Med* 2013; **159**(7): 502-3.
17. Anderson DR, Dunbar M, Murnaghan J, et al. Aspirin or Rivaroxaban for VTE Prophylaxis after Hip or Knee Arthroplasty. *N Engl J Med* 2018; **378**(8): 699-707.
18. Bozic KJ, Vail TP, Pekow PS, Maselli JH, Lindenauer PK, Auerbach AD. Does aspirin have a role in venous thromboembolism prophylaxis in total knee arthroplasty patients? *J Arthroplasty* 2010; **25**(7): 1053-60.
19. Jameson SS, Charman SC, Gregg PJ, Reed MR, van der Meulen JH. The effect of aspirin and low-molecular-weight heparin on venous thromboembolism after hip replacement: a non-randomised comparison from information in the National Joint Registry. *J Bone Joint Surg Br* 2011; **93**(11): 1465-70.
20. Molnar RB, Jenkin DE, Millar MJ, Campbell D, Harris IA. The Australian arthroplasty thromboprophylaxis survey. *J Arthroplasty* 2012; **27**(2): 173-9.
21. EuroQol G. EuroQol--a new facility for the measurement of health-related quality of life. *Health Policy* 1990; **16**(3): 199-208.
22. Murray DW, Fitzpatrick R, Rogers K, et al. The use of the Oxford hip and knee scores. *J Bone Joint Surg Br* 2007; **89**(8): 1010-4.
23. Cimminiello C, Prandoni P, Agnelli G, et al. Thromboprophylaxis with enoxaparin and direct oral anticoagulants in major orthopedic surgery and acutely ill medical patients: a meta-analysis. *Intern Emerg Med* 2017; **12**(8): 1291-305.
24. Jameson SS, Baker PN, Charman SC, et al. The effect of aspirin and low-molecular-weight heparin on venous thromboembolism after knee replacement: a non-randomised comparison using National Joint Registry Data. *J Bone Joint Surg Br* 2012; **94**(7): 914-8.
25. Kulshrestha V, Kumar S. DVT prophylaxis after TKA: routine anticoagulation vs risk screening approach - a randomized study. *J Arthroplasty* 2013; **28**(10): 1868-73.
26. Bawa H, Weick JW, Dirschl DR, Luu HH. Trends in Deep Vein Thrombosis Prophylaxis and Deep Vein Thrombosis Rates After Total Hip and Knee Arthroplasty. *J Am Acad Orthop Surg* 2018; **26**(19): 698-705.
27. Giraudeau B, Ravaud P, Donner A. Sample size calculation for cluster randomized cross-over trials. *Stat Med* 2008; **27**(27): 5578-85.
28. Kelly T-L, Pratt N. A note on sample size calculations for cluster randomised crossover trials with a fixed number of clusters. *Stat Med* 2019; **38**(18): 3342-5.
29. Lyman S, Lee YY, Franklin PD, Li W, Cross MB, Padgett DE. Validation of the KOOS, JR: A Short-form Knee Arthroplasty Outcomes Survey. *Clin Orthop Relat Res* 2016; **474**(6): 1461-71.
30. Lyman S, Lee YY, Franklin PD, Li W, Mayman DJ, Padgett DE. Validation of the HOOS, JR: A Short-form Hip Replacement Survey. *Clin Orthop Relat Res* 2016; **474**(6): 1472-82.
31. Jenkins PJ, Sng S, Brooksbank K, Brooksbank AJ. Socioeconomic deprivation and age are barriers to the online collection of patient reported outcome measures in orthopaedic patients. *Ann R Coll Surg Engl* 2016; **98**(1): 40-4.
32. Turner RM, White IR, Croudace T, Group PIPS. Analysis of cluster randomized cross-over trial data: a comparison of methods. *Stat Med* 2007; **26**(2): 274-89.

33. Forbes AB, Akram M, Pilcher D, Cooper J, Bellomo R. Cluster randomised crossover trials with binary data and unbalanced cluster sizes: application to studies of near-universal interventions in intensive care. *Clin Trials* 2015; **12**(1): 34-44.
34. White IR, Daniel R, Royston P. Avoiding bias due to perfect prediction in multiple imputation of incomplete categorical variables. *Comput Stat Data Anal* 2010; **54**(10): 2267-7

CRISTAL: Aspirin or LMWH for VTE Prophylaxis After Hip or Knee Arthroplasty

Historical Summary of Amendments for Trial Protocol

Previous Protocol (Initial): 28 September 2018

Updated Protocol: 11 January 2019

AMENDMENT 1

1. **ITEM:** Section 3.3. Intervention: Reminder Schedule for 90-day follow-up
   CHANGE: Previously patients were to be reminded at 90 days only for follow-up and this was changed to allow three reminders, at 90 days, 95 days and 100 days
   RATIONALE: This allowed two further attempts of follow-up and aimed to reduce loss to follow-up if initial contact was unsuccessful
2. **ITEM:** Section 3.6. Primary outcome: Question at 90-day follow-up about ongoing anticoagulation
   CHANGE: This question was removed from the 90-day follow-up questionnaire
   RATIONALE: This could not distinguish between patients who were on long-term anticoagulants preoperatively or who had started anticoagulants postoperatively for an alternative reason and was therefore removed
3. **ITEM:** Section 3.8. Participant timeline – Table of Pre-operative/90 day/6 month Questions: Question at 6 months regarding venous thromboembolism (VTE) and deep venous thrombosis (DVT) or pulmonary embolism (PE)
   CHANGE: This question was removed
   RATIONALE: VTE was to be assessed at 90 days only and not 6 months
4. **ITEM:** Section 3.8. Participant timeline – Pre-operative anticoagulation use (Appendix 4): Question 4, timing of previous DVT or PE
   CHANGE: Question updated to remove abbreviations, DVT changed to "deep venous thrombosis" and PE to "pulmonary embolism"
   RATIONALE: Avoid confusion in patients about meaning of abbreviations
5. **ITEM:** Section 3.8. Participant timeline – Post-operative VTE symptoms and occurrence (Appendix 4): Question 7, serious bleeding postoperatively and preceding text "Questions 1 to 3 are only specific for the 90 days data collection point…" implied that VTE and serious bleeding were to be measured at 6 months
   CHANGE: Question 7 moved to Question 5, and preceding text changed to "Questions 1 to 5 are only specific for the 90 days data collection point…"
   RATIONALE: Serious bleeding and postoperative VTE occurrence were only measured at 90 days and not at 6 months
6. **ITEM:** Section 4.3. Consent to project participation – Waiver of Consent – Section e: Protection of patient privacy

CHANGE: Addition of text stating that a third-party security review and penetration test was undertaken of the Australian Orthopaedic Association National Joint Replacement Registry (AOANJRR) clinical trials system prior to data collection
RATIONALE: Provide patients and ethics committees with security that patient privacy was protected prior to trial commencement

7. **ITEM:** Section 4.4. Confidentiality: Protection of patient privacy
   CHANGE: Addition of text stating that a third-party security review and penetration test was undertaken of the AOANJRR clinical trials system prior to data collection
   RATIONALE: Provide patients and ethics committees with security that patient privacy was protected prior to trial commencement

Previous Protocol: 11 January 2019

Updated Protocol: 6 February 2019

AMENDMENT 2

1. **ITEM:** Section 1.3. Contributors
   CHANGE: Addition of two medical students as contributors, Qazi Sarem Shahab and Emma Tsz Lou Cheng
   RATIONALE: Allowed these contributors to assist with the hospital inpatient audit process
2. **ITEM:** Section 3.3. Intervention: Save and complete function added
   CHANGE: Save and complete function added to the electronic data system used for data collection
   RATIONALE: Allowed for patients to save progress on postoperative questionnaires and to log-in again to complete questionnaire for up to 2 weeks after commencement (in case of unexpected stopping so that entered data was not lost)
3. **ITEM:** Section 3.3. Intervention: Dose reduction for low-molecular weight heparin (enoxaparin) based on weight
   CHANGE: Dose for enoxaparin reduced to 20mg for patients weighing less than 50kg
   RATIONALE: After consultation with haematologist and participating surgeon groups/institutions, decision made to reduce dose to 20mg for patients weighing less than 50kg prior to trial commencement
4. **ITEM:** Section 3.4. Adherence: Definition of wound ooze
   CHANGE: Wound ooze defined as ooze occurring beyond 72 hours instead of 36-48 hours postoperatively
   RATIONALE: Aimed to deter surgeons and clinicians from with-holding prophylaxis for postoperative wound ooze occurring within 72 hours of surgery, which occurs frequently

Previous Protocol: 6 February 2019

Updated Protocol: 9 July 2019

AMENDMENT 3

1. **ITEM:** Section 3.2. Eligibility – Patient Level: Update to include patients with fracture
   CHANGE: Addition of patients with diagnosis of fracture to be included
   RATIONALE: Allowed for protocol to be applied to all patients undergoing any hip or knee arthroplasty procedure at participating hospitals to reduce confusion about which patients would be included or not for hospital staff members (nursing staff, junior doctors, residents, registrars and consultants/attending doctors)
2. **ITEM:** Section 3.4. Adherence: Audit of inpatient compliance extended to all sites
   CHANGE: Inpatient compliance changed from "a sample of hospitals" to "all sites"
   RATIONALE: Extended audit process to all sites (clusters) to allow measurement of inpatient compliance across all participating hospitals
3. **ITEM:** Section 3.9. Sample size: Change in number of clusters used for sample size
   CHANGE: Based on number of expected hospitals, cluster number was reduced to 22 hospitals and a new sample size was calculated, giving 212 patients per arm for each hospital for the primary outcome
   RATIONALE: The number of hospitals recruited was less than expected at this time and the sample size was re-calculated
4. **ITEM:** Section 3.12. Blinding: Clarification on how patients will be blinded to outcomes and interventions
   CHANGE: Insertion of phrase "whether they (patients) are in the intervention or control group and the secondary outcomes of the trial"
   RATIONALE: Clarification on how patients would not be aware that they were receiving control or intervention medication for the trial and that this would not bias them when reporting outcomes at 90 days or 6 months
5. **ITEM:** Section 3.15. Statistical Analysis: Update on methods used
   CHANGE: Insertion of use of cluster summary methods
   RATIONALE: Use of cluster summary methods in final statistical analyses of the outcomes

Previous Protocol: 9 July 2019

Updated Protocol: 1 October 2019

AMENDMENT 4 – Protocol published after this amendment (BMJ Open. 2019 Nov 6;9(11):e031657)

1. **ITEM:** Section 1. Administrative Information – 1.1. Registration
   CHANGE: Trial registration number from Australian and New Zealand Clinical Trials Registry (ANZCTR) included
   RATIONALE: Linked protocol to published online protocol through inclusion of ANZCTR number
2. **ITEM:** Section 1.3. Contributors
   CHANGE: Addition of Dr Thu-Lan Kelly as contributor (statistician)
   RATIONALE: Allowed addition of lead statistician prior to development of statistical analysis plan
3. **ITEM:** Section 1.6. Abbreviations
   CHANGE: Included "OA" as abbreviation for osteoarthritis
   RATIONALE: Avoided any misunderstandings of abbreviation of "OA" throughout protocol
4. **ITEM:** Section 3.3 Intervention: Clarification on number of times patients could be contacted for 90 day and 6 month follow-up
   CHANGE: Allowed up to 3 successful attempts to contact patients to complete 90 day and 6 month follow-up surveys and allowed for attempts to contact patients beyond 100 days and 6.5 months if their surveys remained incomplete
   RATIONALE: Allowed for further contact attempts to reduce loss to follow-up
5. **ITEM:** Section 3.6. Primary Outcome: False negative audit
   CHANGE: Included an audit of 200 patients who did not report a VTE to allow estimation of the false negative rate through contact with their general practitioners and treating surgeons
   RATIONALE: False negative audit allowed estimation of whether any VTE's had been missed
6. **ITEM:** Section 3.7. Secondary Outcomes
   CHANGE: Classification of non-VTE complications (death, serious bleeding, joint related re-operation and re-admission within 90 days and death, re-operation within 6 months) and specification of time points at which these would be collected
   RATIONALE: Allowed specification of time points prior to development of statistical analysis plan
7. **ITEM:** Section 3.9. Sample size

CHANGE: Sample size calculation updated to reflect that 31 hospitals had been recruited, which was increased from 22 from the previous protocol. The new sample size used an event rate of 2.5% in the aspirin group, 1.5% in the enoxaparin group, with a non-inferiority margin of 1%, a power of 90% and a one-sided significance of 0.025. Using an intracluster correlation of 0.01, an interperiod correlation of 0.008, the sample size increased to 251 patients per arm per hospital, increasing the overall sample size to 15,562 allowing for a loss to follow-up of up to 27%. A sample size table was also included (Table 1) to allow for a range of parameters for power, non-inferiority margin and event rate.

RATIONALE: The number of hospitals recruited had increased from the last version of the protocol and a new sample size was calculated.

8. **ITEM:** <u>Section 3.9. Sample size:</u> determination of preliminary VTE rate after 1000 recruited patients to help guide sample size

   CHANGE: Calculation of VTE rate was performed after first 1000 patients recruited (without any between-group analyses) to help ensure overall event rate was correct in order to guide sample size for remainder of trial

   RATIONALE: This would allow adjustment of sample size if estimated pre-trial event rate (2%) was incorrect

9. **ITEM:** <u>Section 3.15. Statistical analysis:</u> accounting for missing data and specification of secondary/subgroup analyses

   CHANGE: Section on accounting for missing data using multiple imputation was included in protocol and secondary/subgroup analyses were clarified

   RATIONALE: Clarification of methods for handling missing data and methods used for secondary/subgroup analyses prior to statistical analysis plan

Previous Protocol: 1 October 2019

Updated Protocol: 24 September 2020

AMENDMENT 5 – After protocol publication, amendment made due to meeting of Data Safety Monitoring Board (DSMB), first interim analysis (11 September 2020) and recommendations from lead Human Research Ethics Committee (HREC)

1. **ITEM:** Section 3.2. Eligibility – Exclusion criteria: clarification of exclusion criteria given recommendations from DSMB that the decision to include or exclude patients should be as explicit as possible
   CHANGE: The three dot points at the end of this section were removed and were changed to "At an individual level, patients unsuitable to receive routine prophylaxis will be treated according to local advice and recommendations, as per normal practice. Routine prophylaxis for the purpose of the CRISTAL study includes the low-molecular weight heparin (LMWH) and the aspirin protocols used in CRISTAL. Reasons for not receiving routine prophylaxis include the long-term use of warfarin, novel oral anticoagulants (NOAC) or dual antiplatelet therapy pre-operatively, allergy to the study drug and an underlying medical condition that precludes the use of either drug. Obesity, bilateral surgery or past history of VTE (not currently being treated) alone are not considered sufficient criteria to exempt patients from routine prophylaxis."
   RATIONALE: Change of wording for eligibility criteria as per the recommendations of the DSMB. This was adopted by the Trial Management Committee (TMC) into the trial protocol
2. **ITEM:** Section 4.5. Risk to patients
   CHANGE: Requirement to notify HRECs of the death of any patient participating in CRISTAL
   RATIONALE: Protocol updated to inform HRECs of any death participating in CRISTAL
3. **ITEM:** Section 4.6. Safety Monitoring and Management of Serious Adverse Events:
   CHANGE: Insertion of statement of how serious adverse events would be managed by the TMC, including the establishment of a DSMB and notification of the AOANJRR
   RATIONALE: Updated protocol to include information on how serious adverse events would be managed following first interim analysis

Previous Protocol: 24 September 2020

Updated Protocol (Final): 29 October 2020

AMENDMENT 6 – Final amendments due to further recommendations from lead HREC

1. **ITEM:** Section 3.2. Eligibility – Exclusion criteria: the information from the previous protocol was further edited upon recommendation from the lead human ethics research committee
   CHANGE: The text was changed to "…Reasons for not receiving routine prophylaxis include the long-term use of warfarin, NOAC or dual antiplatelet therapy pre-operatively, allergy to the study drug and an underlying medical condition that precludes the use of either drug or the treating doctors consider the patient to be high risk for routine prophylaxis."
   RATIONALE: Change of wording as per the advice of the lead HREC after review of the recommendations of the DSMB
2. **ITEM:** Section 4.6. Safety Monitoring and Management of Serious Adverse Events:
   CHANGE: Additional wording on how VTE related deaths would be managed, addition of "The DSMB will also be notified as soon as practicable by the relevant principle investigators of any VTE related participant deaths as they become aware of the events. This includes post-surgical inpatient deaths or deaths after discharge of which the Principle Investigator or researcher becomes aware."
   RATIONALE: The TMC agreed that the DSMB should be made aware of any VTE related death as soon as practicable, in addition to notifying the HREC as listed above

STATISTICAL ANALYSIS PLAN (INITIAL): 14 May 2021

TITLE PAGE

Study Title

CRISTAL (A cluster-randomised, crossover, non-inferiority trial of aspirin compared to low molecular weight heparin for venous thromboembolism prophylaxis in hip or knee arthroplasty, a registry nested study): statistical analysis plan

Corresponding Author:

Dr Verinder Singh Sidhu (author)

Postal Address: Whitlam Orthopaedic Research Centre, Level 1, The Ingham Institute for Applied Medical Research, 1 Campbell Street, Liverpool, NSW, 2170

Email address: Verinder.s.sidhu@gmail.com

Phone: +61 422 167 435

Fax Number: +61 2 8738 3850

Co-Authors

2. Dr Thu-Lan Kelly (senior statistician)
   Affiliation: Clinical and Health Sciences, Quality Use of Medicines Pharmacy Research Centre, University of South Australia, Adelaide, South Australia, Australia
3. Dr Nicole Pratt (senior statistician)
   Affiliation: Clinical and Health Sciences, Quality Use of Medicines Pharmacy Research Centre, University of South Australia, Adelaide, South Australia, Australia
4. Professor Steven Graves
   Affiliation: Australian Orthopaedic Association National Joint Replacement Registry, Adelaide, South Australia, Australia
5. Professor Rachelle Buchbinder
   Affiliations:
   i. Department of Epidemiology and Preventive Medicine, Monash University School of Public Health, Melbourne, Victoria, Australia
   ii. Monash Department of Clinical Epidemiology, Cabrini Institute, Melbourne, Victoria, Australia
6. Associate Professor Justine Naylor
   Affiliation: Whitlam Orthopaedic Research Centre, Ingham Institute for Applied Medical Research, South West Sydney Clinical School, The University of New South Wales Sydney, Sydney, NSW, Australia
7. Professor Richard de Steiger
   Affiliation: Department of Surgery, Epworth Healthcare, University of Melbourne, Melbourne, Victoria, Australia
8. Associate Professor Ilana Ackerman

Affiliation: Department of Epidemiology and Preventive Medicine, Monash University School of Public Health and Preventive Medicine, Melbourne, Victoria, Australia

9. Associate Professor Sam Adie
   Affiliation: St. George and Sutherland Clinical School, The University of New South Wales Sydney, Sydney, NSW, Australia
10. Ms Michelle Lorimer
    Affiliation: South Australian Health and Medical Research Institute, Adelaide, South Australia, Australia
11. Mrs Durga Bastiras
    Affiliation: Australian Orthopaedic Association National Joint Replacement Registry, Adelaide, South Australia, Australia
12. Ms Kara Cashman
    Affiliation: South Australian Health and Medical Research Institute, Adelaide, South Australia, Australia
13. Professor Ian Harris (chief investigator)
    Affiliations:
    i. Whitlam Orthopaedic Research Centre, Ingham Institute for Applied Medical Research, South West Sydney Clinical School, The University of New South Wales Sydney, Sydney, NSW, Australia
    ii. Institute of Musculoskeletal Health, School of Public Health, The University of Sydney, Sydney, NSW, Australia

TABLE OF CONTENTS

This supplement contains the following items:

iv. Initial Statistical Analysis Plan Pages 3 – 26

v. Final Statistical Analysis Plan Pages 27 – 51

vi. Summary of Changes Pages 52 – 54

ABSTRACT

Background:
This *a priori* statistical analysis plan describes the analysis for CRISTAL .

Methods:
CRISTAL (cluster-randomised, crossover, non-inferiority trial of aspirin compared to low molecular weight heparin for venous thromboembolism prophylaxis in hip or knee arthroplasty, a registry nested study) aims to determine whether aspirin is non-inferior to low molecular weight heparin (LMWH) in preventing symptomatic venous thromboembolism (VTE) following hip arthroplasty (HA) or knee arthroplasty (KA). The study is nested within the Australian Orthopaedic Association National Joint Replacement Registry. The trial was commenced in April 2019 and after an unplanned interim analysis, recruitment was stopped (December 2020), as the stopping rule was met for the primary outcome.

The clusters comprised hospitals performing > 250 HA and/or KA procedures per annum, whereby all adults (> 18 years) undergoing HA or KA were recruited. Each hospital was randomised to commence with aspirin, orally, 85-150mg daily or LMWH (enoxaparin), 40mg, subcutaneously, daily within 24 hours postoperatively, for 35 days after HA and 14 days after KA. Crossover was planned once the registration target was met for the first arm.

The primary end point is symptomatic VTE within 90 days. Secondary outcomes include readmission, reoperation, major bleeding and death within 90 days, and reoperation and patient-reported pain, function and health status at 6 months.

The main analyses will focus on the primary and secondary outcomes for patients undergoing elective primary total HA and KA for osteoarthritis. The analysis will use an intention-to-treat approach with cluster summary methods to compare treatment arms. As the trial stopped early, analyses will account for incomplete cluster crossover and unequal cluster sizes.

Conclusions:
This paper provides a detailed statistical analysis plan for CRISTAL.

Trial registration: Australian and New Zealand Clinical Trials Registry, ID: ACTRN12618001879257. Registered on 19/11/2018.

Key Words
Venous Thromboembolism, Hip Arthroplasty, Knee Arthroplasty, Aspirin, Low Molecular Weight Heparin, Statistical Analysis Plan

MANUSCRIPT

Background
Despite the increasing use of aspirin as a sole chemotherapeutic agent for symptomatic venous thromboembolic event (VTE) prophylaxis following hip arthroplasty (HA) and knee arthroplasty (KA) [1], there remains limited high quality comparative evidence for its safety and efficacy. The majority of studies supporting the safety and efficacy of aspirin compared to other agents, including low molecular weight heparin (LMWH), have been retrospective or non-randomised [2-11]. The only randomised trials have been underpowered or have used an alternative form of prophylaxis (e.g., LMWH or a novel oral anticoagulant (NOAC)) for the immediate postoperative period following HA or KA prior to changing to aspirin for extended prophylaxis, which does not reflect the way aspirin is used in Australia [12, 13]. CRISTAL is a pragmatic, multicentre cluster-randomised, crossover trial that aims to determine if aspirin is non-inferior to LMWH in the prevention of symptomatic VTE following HA and KA. It is nested within the Australian Orthopaedic Association National Joint Replacement Registry (AOANJRR).

The trial commenced in April 2019 and the estimated timeline for completion of patient registration was 24 months. However, after an unplanned interim analysis in which the trial stopping rule was met, patient registration was ceased in December 2020, resulting in incomplete crossover. This statistical analysis plan details the planned analyses for CRISTAL to facilitate transparency of data analysis. The trial protocol has previously been published [14].

STUDY OVERVIEW

Ethics
Ethics approval was granted from all relevant central, lead ethics committees involved and all participating hospitals, as outlined in the published trial protocol [14]. The trial is registered with the Australian New Zealand Clinical Trials Registry (ACTRN12618001879257p) and is endorsed by the Australia and New Zealand Musculoskeletal (ANZMUSC) Clinical Trials Network.

Participating Hospitals and Patient Registration
The clusters in CRISTAL comprise 31 consenting hospitals that perform greater than 250 HA and/or KA procedures per annum.

Each recruited hospital was responsible for registering patients and complying with the trial protocol. The AOANJRR routinely collects data pertaining to the procedure, patient age, sex, American Society of Anaesthesiologists (ASA) class and body mass index (BMI) and death on all patients undergoing HA and KA procedures. Patient-reported outcomes are collected through the electronic Clinical Trials Platform, which requires pre-operative registration of the patient onto the electronic system. All adult (age 18 and older) patients undergoing HA or KA were eligible for registration into the study and eligible to receive the allocated study drug, except for those who were already on long-term anticoagulation (specifically a NOAC, warfarin or dual antiplatelet therapy (DAPT)) and those with a medical contraindication to

either drug, e.g., an allergy or a medical comorbidity such as thrombophilia that precluded treatment with the study drug.

Patients who were not registered in the electronic Clinical Trials Platform will be included in secondary analyses, as procedure information, demographics and mortality were still recorded even though the primary outcome and other patient-reported outcomes were not recorded.

Intervention

Each hospital (cluster) was allocated to consecutive periods of a standard protocol of LMWH and a standard protocol of aspirin as VTE prophylaxis, with the order being randomised. Patients in the aspirin group received aspirin at 85-150mg once daily, orally for 35 days post HA and for 14 days post KA, commencing within 24 hours of surgery. Patients in the LMWH group received enoxaparin at 40mg once daily, subcutaneously for the same time periods, with this dose reduced to 20mg for patients who weigh less than 50kg and for patients with an estimated glomerular filtration rate (eGFR) of less than 30mL/min who are not on dialysis. Other interventions that were standard across all sites were the intra- and post-operative use of intermittent pneumatic compression (IPC) calf devices until patients are mobile, the use of compression stockings, and mobilisation offered on day 0 or day 1 postoperatively.

Randomisation and allocation

Study investigators have remained blinded to group allocation. Hospitals were randomised to commence with either LMWH or aspirin, in randomly permuted blocks of size four by statisticians from the South Australian Health and Medical Research Institute (SAHMRI), independent of study investigators. The randomisation sequence was generated using an online application [15] and this was provided to an unblinded data manager from SAHMRI. The hospital was then allocated to a treatment sequence by SAHMRI staff and this information was provided to the AOANJRR (independent of study investigators), with the site being informed of their allocated treatment arm the week prior to commencing initial patient registration. Hospitals were advised to crossover to the alternate treatment once the sample size for the first treatment arm was met.

For clusters who did not reach the sample size for the first arm within 18 months of commencement, crossover occurred prior to reaching the sample size so that an equal number of patients could be registered in each arm within the study timeframe.

Evaluation of adherence to the study protocol and protocol deviations

At a hospital level, during the course of the trial each hospital was audited within the first month of each treatment arm to ensure they were complying with the trial protocol. The audit consisted of the first 20 patients of each treatment arm. If a site had a compliance of less than 80%, the site was educated on methods of improving protocol compliance and subsequently re-audited until compliance to the protocol was above 80%.

Hospitals were also advised to inform trial co-ordinators of patients not receiving the correct study drug or those patients who had the study drug withheld for greater than 48

hours due to side effects (e.g. allergy, excessive wound drainage or bleeding events). These protocol deviations were recorded using the Clinical Trials Platform.

Outcome variables
The primary outcome of the study is symptomatic VTE within 90 days of surgery. Secondary outcomes are:

- Deep vein thrombosis (DVT) only (total, below-knee and above-knee) within 90 days
- Pulmonary embolism (PE) only within 90 days
- Readmission related to the original surgery or associated treatment (including bleeding and VTE-related) within 90 days
- Reoperation on the same joint within 90 days and within 6 months of surgery
- Major bleeding events within 90 days defined as bleeding events resulting in readmission, reoperation or death
- Death within 90 days
- Change in patient-reported pain, function and health status measures as measured by the Oxford Hip Score (OHS), Oxford Knee Score (OKS), EQ-5D score and the EQ-VAS from baseline to 6 months postoperatively

Outcome and demographic data were collected preoperatively (demographics, patient reported pain, function and health status) and at 90 days and 6 months postoperatively. Data for all primary and secondary outcomes are patient-reported (except for death). All patients who responded ‘yes’ to having experienced a VTE or a secondary operation within 6 months had this result verified by AOANJRR staff through contact with treating doctors and hospitals. A random audit of 200 patients who did not report a VTE event was undertaken to detect the false negative reporting rate. All data collected for registered patients specific to CRISTAL have been outlined in the published protocol [14]. Mortality data were collected through linkage between the AOANJRR and the National Death Index.

In the published protocol [14], mortality was to be measured at 90 days and 6 months. Due to the lack of sensitivity in measuring VTE-related mortality at 6 months, and due to the lag in data availability for mortality, we will only analyse mortality at 90 days [16].

Power and sample size
For the sample size calculation in CRISTAL, we used an estimated overall event rate of 2% (based on the current available literature) [17, 18], a non-inferiority margin of 1% (based on clinician opinion and a recent randomised controlled trial) [12], i.e., an event rate of 2.5% for aspirin and 1.5% for LMWH, a power of 90% and a one-sided significance level of 0.025. For an individual randomised trial, this yields a sample size of 4,117 per treatment group or a total of 8,234 patients. For a cluster-randomised crossover trial with an intracluster correlation of 0.01, an interperiod correlation of 0.008 and 31 clusters, the required sample size is 11,160 patients [19, 20]. From each cluster and from each arm, we aimed to register 251 patients eligible for the primary objective of the study [14]. This provided a total of 15,562 patients and allows for a 27% loss to follow-up.

STATISTICAL ANALYSIS PLAN

Patient Populations and Subgroups for Analyses

The total patient population for CRISTAL comprises all patients undergoing HA or KA at participating institutions over the duration of the study, regardless of whether these patients were registered or eligible to receive the study drug (defined as population 5, see Figure 1).

Within this total population, the following populations will be used to form the basis of the analyses:

- Registered patients undergoing any form of HA or KA (including partial or revision surgery, for any indication) regardless of eligibility to receive the study drug (population 4)
- Registered patients undergoing any form of HA or KA (including partial or revision surgery, for any indication) who were eligible to receive the study drug (population 3)
- Registered patients undergoing elective primary THA or TKA (for any indication) who were eligible to receive the study drug (population 2)
- All registered patients undergoing elective primary THA or TKA for a recorded diagnosis of osteoarthritis (OA) who were eligible to receive the study drug (population 1)

These populations are represented diagrammatically in Figure 1.

Figure 1. Patient populations within CRISTAL

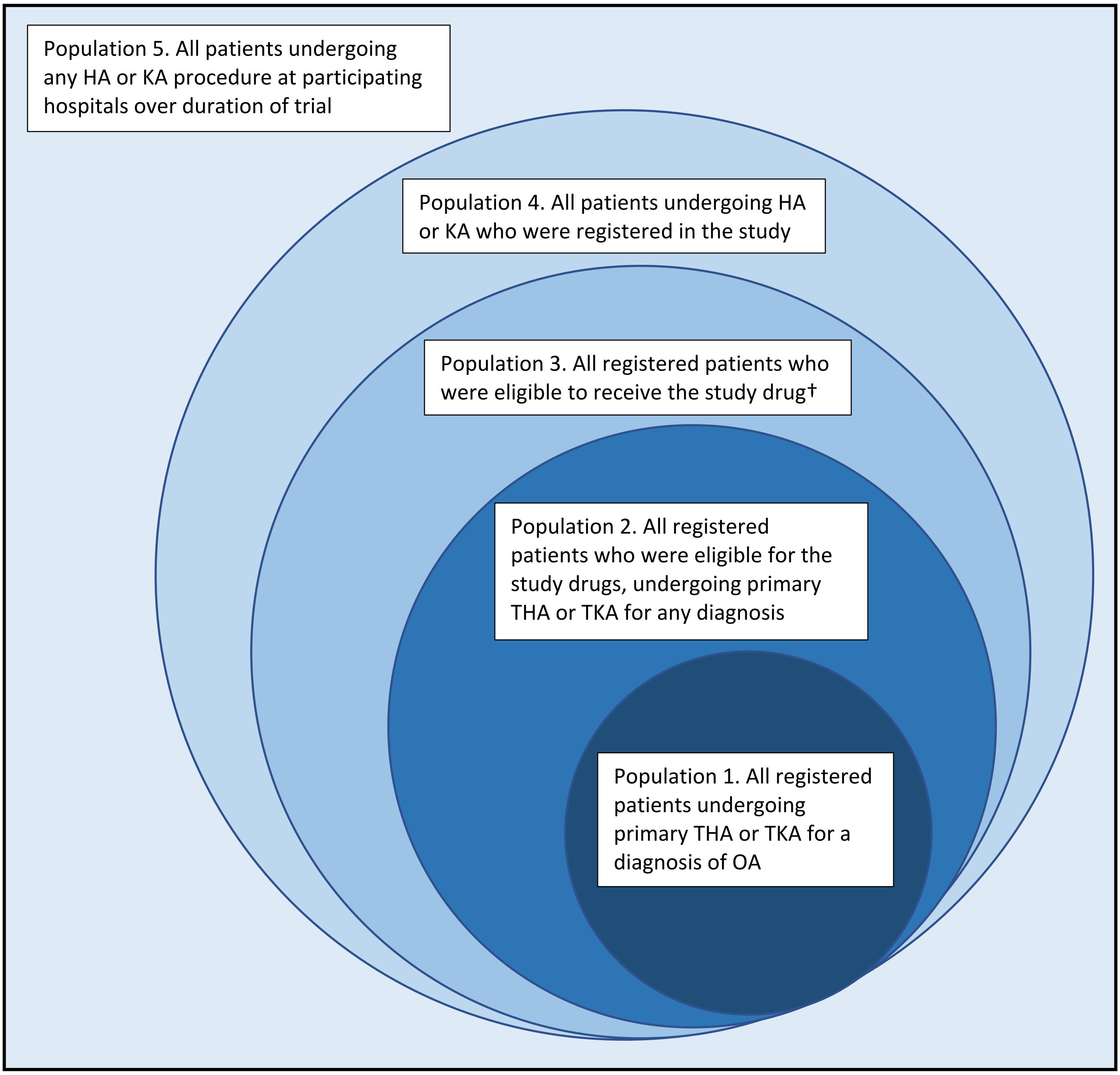

Legend:

Abbreviations: *HA* hip arthroplasty, *KA* knee arthroplasty, *THA* total hip arthroplasty, *TKA* total knee arthroplasty, *OA* osteoarthritis

† Study drug excluded for patients who already on long-term anticoagulation (specifically a novel oral anticoagulant – NOAC, warfarin or dual antiplatelet therapy – DAPT) and those who have a medical contraindication

Each outcome (primary and secondary) will be assessed for populations 1, 2, 3 and 4 listed in Figure 1. Mortality will be assessed for all populations (including population 5). The primary objective of the study as outlined in the published protocol [14], was the analysis of population 1 only (registered patients undergoing primary THA or TKA for a diagnosis of OA who are eligible to receive the study drug), as this was the focus of the sample size calculation. This population will remain the focus of the main analyses.

Population 1 was chosen as the focus of the main analysis as these patients represent the majority of patients undergoing HA or KA procedures and there are known differences in outcomes and co-morbidities with other diagnoses (e.g., fracture, tumour), which could confound the primary outcome [21].

For the primary end point of VTE, the following subgroup analyses will be conducted within the corresponding populations listed:

- Type of joint replacement: primary THA compared to primary TKA – population 1
- Bilateral arthroplasty: patients undergoing simultaneous bilateral arthroplasty compared to those who are not – population 1
- Revision arthroplasty: patients undergoing revision hip or knee arthroplasty compared to those undergoing primary arthroplasty – population 3
- Prior history of VTE: patients with a prior history of VTE compared to those without – population 1

Analysis principles

Data will be analysed according to the intention-to-treat principle with clusters analysed according to assigned group allocation. Although hospital and patient protocol deviations will be recorded, no as-treated analyses will be performed, as there are no verified data available to determine whether individual patients received the assigned study drug for the full period, given the pragmatic nature of the trial. The timing of analyses will be stratified by follow-up time of the outcomes measured (90 days and 6 months). The difference in absolute risk for symptomatic VTE between each group and 95% confidence intervals (upper and lower) will be examined to determine if the non-inferiority margin is met.

Continuous variables will be summarised using standard measures of central tendency and dispersion, using either mean and standard deviation or median and interquartile range. Categorical variables will be summarised by frequencies and percentages.

Analyses will be performed using SAS version 9.4 (SAS Institute, Cary USA) and R (R Foundation for Statistical Computing Platform) version 4.0.2 or higher.

Interim analysis

An interim analysis was not initially planned, as both treatments are considered standard practice for VTE prophylaxis in Australia and the trial is investigating an adverse event as the primary outcome. However, due to concerns of an increased adverse event rate (symptomatic VTE and death) in one of the prophylaxis groups, a Data Safety Monitoring

Board (DSMB) was convened one year into patient recruitment. The DSMB consisted of an orthopaedic surgeon, a haematologist and a statistician, all independent of the trial.

The DSMB were advised by the Trial Management Committee (TMC) to conduct an interim analysis. In conjunction with the DSMB (prior to the interim analysis), the TMC applied the Haybittle-Peto stopping rule of a two-sided significance of 0.001 for the primary outcome in the population 1 [22, 23]. This stopping rule was chosen as it does not require adjustment of the significance threshold for the final analysis and allows further interim analyses using the same threshold (if required).

After the first interim analysis (in September 2020), the DSMB recommended continuing the trial and performing a second interim analysis in November 2020. After reviewing the second interim analysis, the DSMB recommended ceasing patient recruitment as the stopping rule had been met. The study ceased recruiting patients in December 2020 and sites reverted to their usual VTE prophylaxis pathways.

Methods used for Interim Analyses

Interim analyses were conducted for VTE and mortality within 90 days for population 1. To account for unequal cluster sizes, incomplete crossover or clusters which had not yet crossed over, a composite analysis was designed. For clusters which had crossed over, including with partial completion of the second period, the cluster weighted estimator intended for the primary outcome was used. Clusters which had not crossed over were analysed using the cluster period summaries, weighted by cluster size, in a parallel design approach. Estimates for the two approaches were combined using inverse variance weights to provide a final estimate. Confidence intervals were constructed using the Haybittle-Peto boundary of 0.001.

Data integrity

Integrity of data will be checked prior to conducting the final analysis. The data set will be checked for errors, omissions and double data entry. These will be resolved prior to commencing the analysis in consultation with the data management plan [14].

Blinding

The DSMB were blinded to treatment allocation (groups in the interim analyses were labelled A and B). All researchers involved in the preparation of this analysis plan will have no access to trial data broken down by treatment allocation for the final statistical analysis. Once data integrity checks have been conducted, a blind review to quantify missing data of the entire dataset will be conducted and any final amendments to the statistical analysis plan will be made before the database is locked. During analysis and interpretation, group allocation will be masked by dummy group names and the true allocation will be unmasked only after the final statistical report has been completed and interpretation has been agreed to by the writing group and minuted.

Methods for handling missing data

Multiple imputation using chained equations will be used to account for missing data, using auxiliary variables gathered from routine AOANJRR data (including age, sex, baseline health, pain and function, diagnosis and surgical factors). If there is any possibility of bias due to

perfect prediction of rare outcomes such as VTE [24] or imputing values out of range for bounded variables such as pain scores or EQ5D [25], imputation will not be performed. Since the most likely reason for loss to follow-up is difficulty in contacting patients postoperatively (rather than association with treatment assignment or outcome), missing data will be assumed to be missing at random.

Trial profile and baseline characteristics

The flow of participating hospitals through the study and participating patients will be reported in line with the Consolidated Standards of Reporting Trials (CONSORT) statement (Figures 2 and 3).

The number of patients registered for population 1 (the population used for the sample size calculation) by each participating hospital and the overall registration rate of each hospital will be presented as outlined in Table 1. The overall registration rate describes the number of registered patients undergoing any HA or KA procedure (population 4) divided by the number of patients who underwent any HA or KA procedure over the duration of the trial at participating hospitals (regardless of whether they were registered – population 5). Hospital names will remain anonymous.

Descriptive statistics of baseline patient characteristics for all registered patients eligible to receive the study drug (population 3) will be presented by prophylaxis group (Table 2).

Figure 2. Flowsheet of participating hospitals

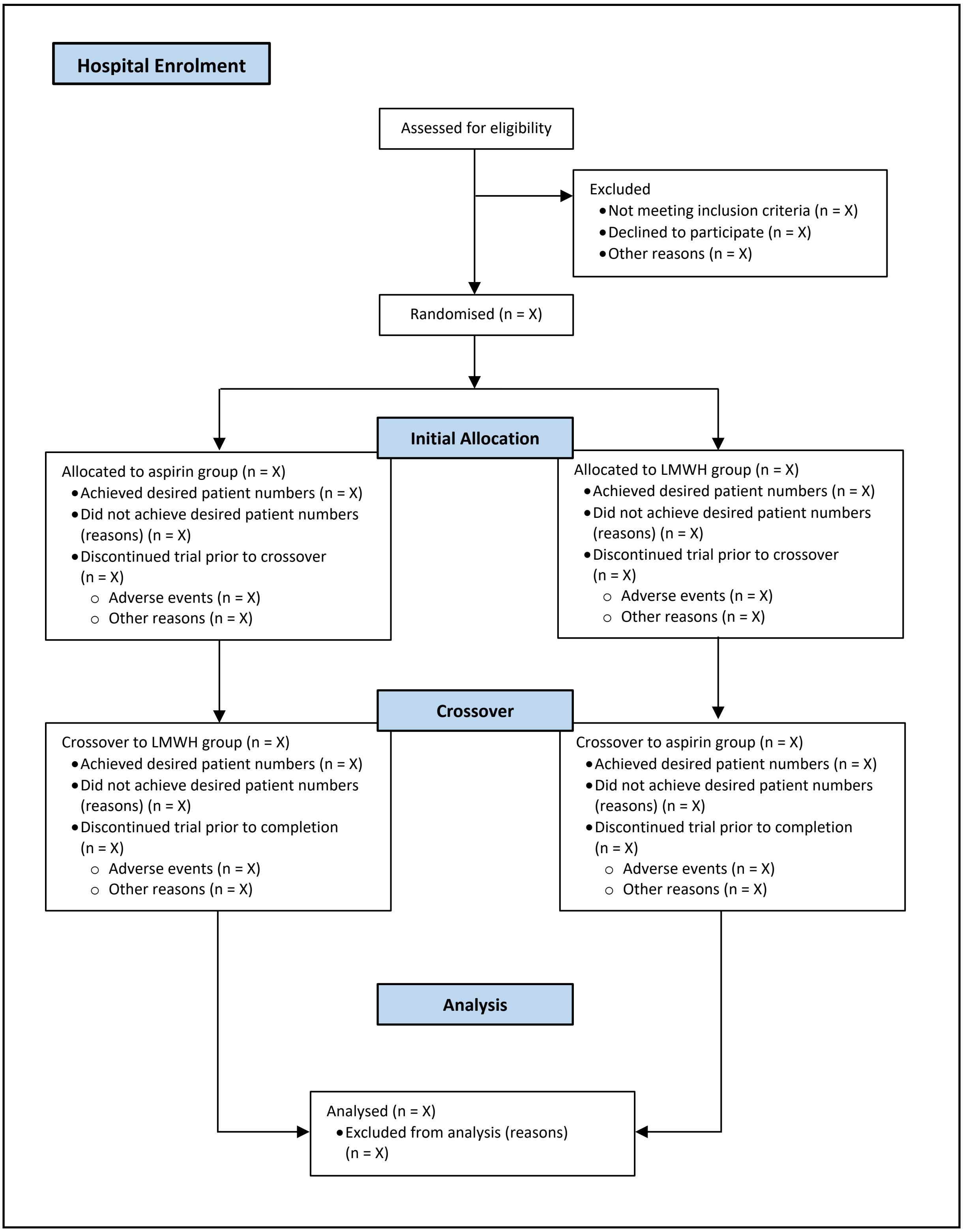

Figure 3. Flowsheet of patients within population 1[†]

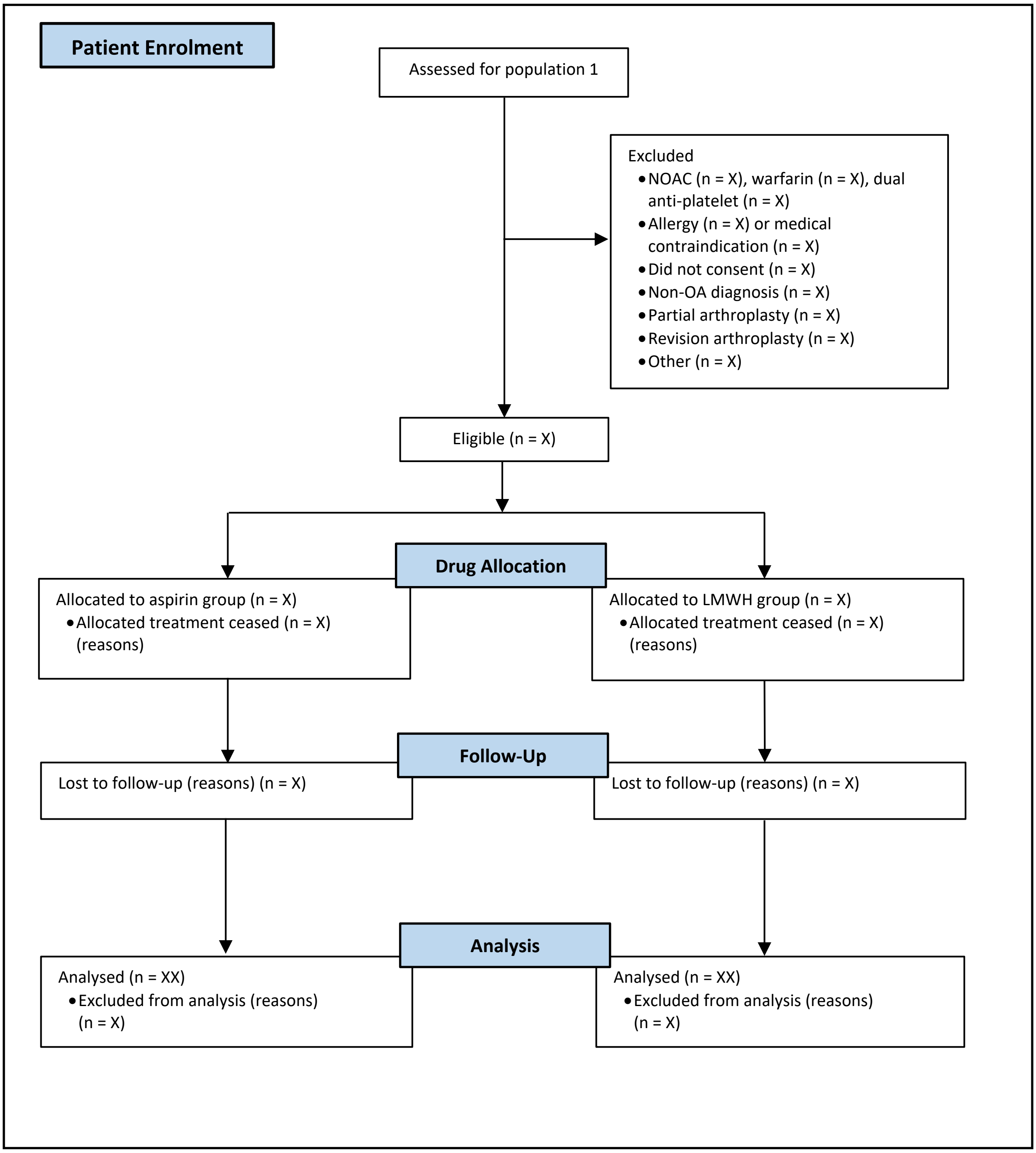

[†] Population 1 refers to registered patients undergoing primary THA or TKA for a diagnosis of OA, who are eligible to receive the study drug

Table 1. Number of registered patients for population 1† by treatment group and overall registration rate for each participating hospital

Hospital	Sequence Allocation?	LMWH Group (Population 1)	Aspirin Group (Population 1)	Overall Registration Rate (combined for both groups)
1		n	n	%
2		n	n	%
3		n	n	%
4		n	n	%
5		n	n	%
6		n	n	%
7		n	n	%
8		n	n	%
9		n	n	%
10		n	n	%
11		n	n	%
12		n	n	%
13		n	n	%
14		n	n	%
15		n	n	%
16		n	n	%
17		n	n	%
18		n	n	%
19		n	n	%
20		n	n	%
21		n	n	%
22		n	n	%
23		n	n	%
24		n	n	%
25		n	n	%
26		n	n	%
27		n	n	%
28		n	n	%
29		n	n	%
30		n	n	%
31		n	n	%
**Total**		**n**	**n**	**%**

† Population 1 refers to registered patients undergoing primary THA or TKA for a diagnosis of OA, who are eligible to receive the study drug

Table 2. Baseline patient characteristics for all registered patients eligible to receive study drug (population 3), according to treatment allocation

	**LMWH**<br>(n = X)	**Aspirin**<br>(n = X)
Age (years)	xx.x (xx.x – xx.x), n	xx.x (xx.x – xx.x), n
BMI (kg/m^2)	xx.x (xx.x – xx.x), n	xx.x (xx.x – xx.x), n
Male sex	n (%)	n (%)
ASA Grading		
1	n (%)	n (%)
2	n (%)	n (%)
3	n (%)	n (%)
4	n (%)	n (%)
5	n (%)	n (%)
Previous venous thromboembolism	n (%)	n (%)
Long term anticoagulant use		
Aspirin	n (%)	n (%)
Other single antiplatelet	n (%)	n (%)
Joint replacement		
THA	n (%)	n (%)
TKA	n (%)	n (%)
Other HA	n (%)	n (%)
Other KA	n (%)	n (%)
Bilateral	n (%)	n (%)
Type of surgery		
Primary total	n (%)	n (%)
Primary partial	n (%)	n (%)
Primary resurfacing	n (%)	n (%)
Revision	n (%)	n (%)
Other	n (%)	n (%)
Indication		
Osteoarthritis	n (%)	n (%)
Inflammatory	n (%)	n (%)
Avascular Necrosis	n (%)	n (%)
Fracture	n (%)	n (%)
Other	n (%)	n (%)
Prosthesis		
Cemented	n (%)	n (%)
Hybrid	n (%)	n (%)
Uncemented	n (%)	n (%)
Pain and Function		
Oxford Hip Score	xx.x (xx.x – xx.x), n	xx.x (xx.x – xx.x), n
Oxford Knee Score	xx.x (xx.x – xx.x), n	xx.x (xx.x – xx.x), n
EQ-5D	xx.x (xx.x – xx.x), n	xx.x (xx.x – xx.x), n
EQ-VAS	xx.x (xx.x – xx.x), n	xx.x (xx.x – xx.x), n

Abbreviations: *BMI* body mass index, *ASA* American society of anaesthesiologists

MAIN ANALYSES

The main analyses will include the primary and secondary outcomes for registered patients eligible to receive the study drug undergoing THA or TKA for a diagnosis of OA (population 1). In addition, the primary and secondary outcomes will be analysed for populations 2, 3 and 4. Mortality will also be analysed for population 5 (see “Additional analyses” below).

For the primary outcome, the analysis will test the between-group difference of cases developing a symptomatic VTE within 90 days for non-inferiority of aspirin at a margin of 1%. Cluster summary methods will be used to estimate the treatment effect using cluster level differences. These have been shown to be appropriate for cluster-randomised crossover trials with rare outcomes, and the intracluster and interperiod correlation coefficients expected in this trial. The crossover difference per cluster is the mean outcome for the intervention period minus the mean outcome for the control period. In a linear regression of cluster differences on treatment sequence, the treatment effect estimate is the intercept. To account for potential unequal cluster sizes, a cluster size weighted estimator will be used with harmonic mean weights of the number of patients in the two periods [26, 27]. Treatment effects will be presented as absolute risk differences and 95% confidence intervals will be examined to determine whether the non-inferiority margin has been met and whether superiority of one drug can be concluded. The primary outcome will be presented in Table 3 and Figure 4 will be used to demonstrate whether the non-inferiority margin has been met for the population 1 [28].

The secondary outcomes will investigate non-VTE complications (death, re-operation, readmission and major bleeding events) within 90 days, and reoperation and patient-related pain and function at 6 months (OHS, OKS, EQ-5D and EQ-VAS). Cluster summary methods will be used within an intention-to treat approach. For binary outcomes, the cluster mean per period will be the proportion of patients who had the outcome, while for continuous outcomes such as pain score, the cluster mean will be the mean outcome. Treatment effects will be presented as absolute risk differences and 95% confidence intervals to determine if one treatment is superior to the alternative. Results for the secondary outcomes in population 1 will be presented in tabular form (Table 3) and results for the primary and secondary outcomes in populations 2, 3 and 4 in Table 4.

Due to the early stopping of the trial, final analyses of the primary and secondary outcomes will use the composite method for the interim analyses with appropriate confidence intervals.

Table 3. Outcomes for population 1

Outcome	LMWH Allocation (n = X)	Aspirin Allocation (n = X)	Absolute Risk Difference	95% Confidence Interval	p-value
**Any Venous thromboembolism**	n (%)	n (%)	X	X – X	
**Type of Venous thromboembolism**					
Pulmonary embolism	n (%)	n (%)	X	X – X	
Deep venous thrombosis	n (%)	n (%)	X	X – X	
Both Pulmonary embolism and deep venous thrombosis	n (%)	n (%)	X	X – X	
Above knee deep venous thrombosis	n (%)	n (%)	X	X – X	
Below knee deep venous thrombosis	n (%)	n (%)	X	X – X	
Death	n (%)	n (%)	X	X – X	
Re-operation (90d)	n (%)	n (%)	X	X – X	
Reoperation (6 months)	n (%)	n (%)	X	X – X	
Re-admission	n (%)	n (%)	X	X – X	
Major Bleeding	n (%)	n (%)	X	X – X	
Pain and Function (median and IQR)†			X	X – X	
Oxford Hip Score	X (X – X)	X (X – X)	X	X – X	
Oxford Knee Score	X (X – X)	X (X – X)	X	X – X	
EQ-5D	X (X – X)	X (X – X)	X	X – X	
EQ-VAS	X (X – X)	X (X – X)			

† at 6 months

Figure 4. Between group change in overall 90-day symptomatic VTE rate and non-inferiority margin. The dotted line represents the non-inferiority margin

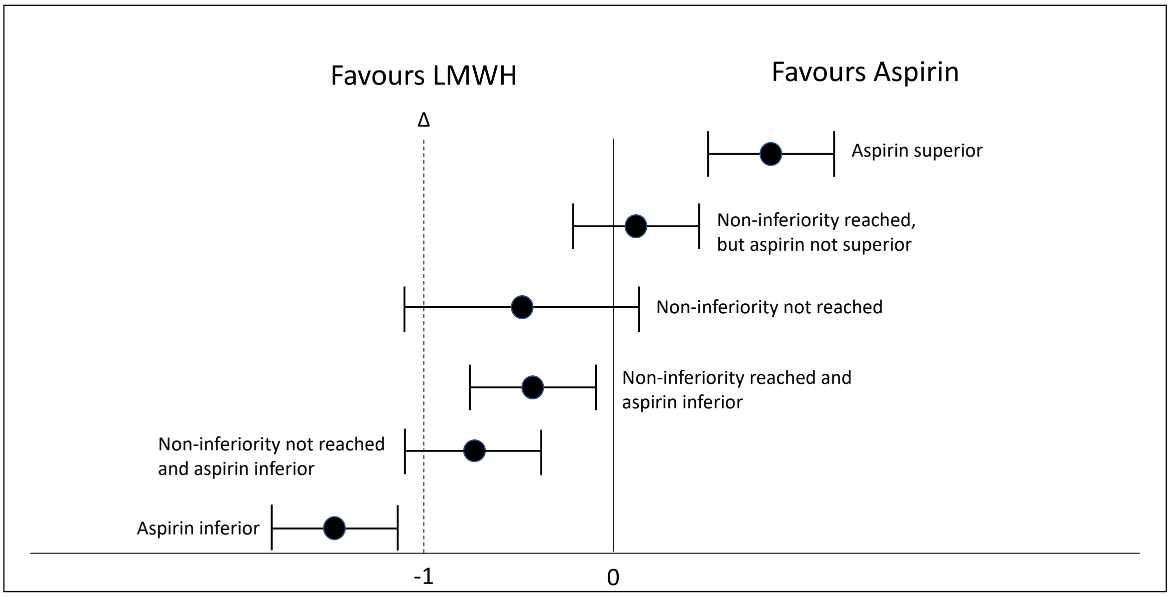

Table 4. Outcomes for populations 2, 3 and 4

Population	Outcome	LMWH Allocation (n = X)	Aspirin Allocation (n = X)	Absolute Risk Difference	95% Confidence Interval	p-value
All primary THA/TKA for any diagnosis eligible to receive study drug (population 2)	**Any Venous thromboembolism**	n (%)	n (%)	X	X – X	
	**Type of Venous thromboembolism**					
	Pulmonary embolism	n (%)	n (%)	X	X – X	
	Deep venous thrombosis	n (%)	n (%)	X	X – X	
	Both Pulmonary embolism and deep venous thrombosis	n (%)	n (%)	X	X – X	
	Above knee deep venous thrombosis	n (%)	n (%)	X	X – X	
	Below knee deep venous thrombosis	n (%)	n (%)	X	X – X	
	Death	n (%)	n (%)	X	X – X	
	Re-operation (90d)	n (%)	n (%)	X	X – X	
	Reoperation (6 months)	n (%)	n (%)	X	X – X	
	Re-admission	n (%)	n (%)	X	X – X	
	Major Bleeding	n (%)	n (%)	X	X – X	
	Pain and Function (median and IQR)†					
	Oxford Hip Score			X	X – X	
	Oxford Knee Score	X (X – X)	X (X – X)	X	X – X	
	EQ-5D	X (X – X)	X (X – X)	X	X – X	
	EQ-VAS	X (X – X)	X (X – X)	X	X – X	
		X (X – X)	X (X – X)			
All HA/KA eligible to receive study drug (population 3)	**Any Venous thromboembolism**	n (%)	n (%)	X	X – X	
	**Type of Venous thromboembolism**					
	Pulmonary embolism	n (%)	n (%)	X	X – X	
	Deep venous thrombosis	n (%)	n (%)	X	X – X	
	Both Pulmonary embolism and deep venous thrombosis	n (%)	n (%)	X	X – X	
	Above knee deep venous thrombosis	n (%)	n (%)	X	X – X	
	Below knee deep venous thrombosis	n (%)	n (%)	X	X – X	
	Death	n (%)	n (%)	X	X – X	
	Re-operation (90d)	n (%)	n (%)	X	X – X	
	Reoperation (6 months)	n (%)	n (%)	X	X – X	
	Re-admission	n (%)	n (%)	X	X – X	
	Major Bleeding	n (%)	n (%)	X	X – X	
	Pain and Function (median and IQR)†					
	Oxford Hip Score			X	X – X	
	Oxford Knee Score	X (X – X)	X (X – X)	X	X – X	
	EQ-5D	X (X – X)	X (X – X)	X	X – X	
	EQ-VAS	X (X – X)	X (X – X)	X	X – X	
		X (X – X)	X (X – X)			
All HA/KA including study drug exclusion (population 4)	**Any Venous thromboembolism**	n (%)	n (%)	X	X – X	
	**Type of Venous thromboembolism**					
	Pulmonary embolism	n (%)	n (%)	X	X – X	
	Deep venous thrombosis	n (%)	n (%)	X	X – X	
	Both Pulmonary embolism and deep venous thrombosis	n (%)	n (%)	X	X – X	
	Above knee deep venous thrombosis	n (%)	n (%)	X	X – X	
	Below knee deep venous thrombosis	n (%)	n (%)	X	X – X	
	Death	n (%)	n (%)	X	X – X	
	Re-operation (90d)	n (%)	n (%)	X	X – X	
	Reoperation (6 months)	n (%)	n (%)	X	X – X	
	Re-admission	n (%)	n (%)	X	X – X	
	Major Bleeding	n (%)	n (%)	X	X – X	
	Pain and Function (median and IQR)†					
	Oxford Hip Score			X	X – X	
	Oxford Knee Score	X (X – X)	X (X – X)	X	X – X	
	EQ-5D	X (X – X)	X (X – X)	X	X – X	
	EQ-VAS	X (X – X)	X (X – X)	X	X – X	
		X (X – X)	X (X – X)			

† at 6 months

Subgroup Analyses
Subgroup analyses for the primary outcome will include THA or TKA, bilateral or unilateral procedures and a prior history of VTE or not for population 1, and primary arthroplasty or revision arthroplasty for population 3.

To assess treatment effects for each subgroup separately, cluster summaries will be produced for each subgroup. An interaction term between treatment group and subgroup (e.g., THA/TKA, bilateral/unilateral) will be added to the model for the primary outcome. The treatment differences for each subgroup will be assessed for non-inferiority. Since the trial was stopped early, the same composite method for the primary outcome will be used.

Sensitivity analyses
Sensitivity analyses will be performed to determine: (a) the effect of high-volume arthroplasty sites; (b) sites with high and low overall registration rates; (c) sites that required multiple compliance audits; and (d) the effect of patients who take long-term aspirin therapy on the results of the analyses for the primary outcome in population 1.

Order of planned analyses
Analyses will be performed in the following order:

- Interim analyses of population 1
- Primary and secondary outcomes for population 1
- Subgroup analyses for population 1
- Primary and secondary outcomes for populations 2, 3 and 4
- Subgroup analyses of population 3
- Sensitivity analyses in population 1

ADDITIONAL ANALYSES

Mortality Analysis

In addition to analysing between-group mortality for populations 1, 2, 3 and 4, the between-group 90-day mortality will be analysed for two further populations:

1. All patients undergoing HA or KA over the duration of the study at participating hospitals, regardless of whether they were registered (total population described above, population 5)
2. All patients undergoing elective THA or TKA over the duration of the study at participating hospitals regardless of whether they were registered (a subset of population 5)

Analysing these additional populations will assess the effect of implementing the VTE prophylaxis protocol on mortality at an institutional/departmental level (the unit of randomisation), on an intention-to-treat basis.

Sub-Studies

Data from this trial will be used to form the basis of sub-studies. These will include a sub-study comparing rates of persistent wound drainage between LMWH and aspirin groups at two participating sites and a sub-study investigating rates of post-hospital discharge compliance to either study drug.

Conclusions

CRISTAL aims to provide much needed definitive evidence about the effectiveness and safety of aspirin compared to LMWH in preventing symptomatic VTE following HA or KA. This statistical analysis plan details the study’s planned analyses, including modifications to intended analyses to account for early stopping of the trial.

DECLARATIONS

Ethics Approval

Ethics approval was granted by the Sydney Local Health District (Royal Prince Alfred Zone) Human Research and Ethics Committee, which is a lead ethics committee in Australia (approval number X18-0424) prior to study commencement. Site-specific approvals for each participating hospital were granted from the following ethics committees prior to study commencement: Calvary John James Memorial Hospital Australian Capital Territory (3-2019 CRISTAL), Mid-North Coast Local Health District New South Wales (NSW – SSA/19/NCC/41), South Western Sydney Local Health District (NSW, SSA/10/LPOOL/22), Sydney Local Health District (NSW, SSA/19/RPAH/12, SSA/18/RPAH/762), Ramsay Hospital Research Foundation (NSW, HREC/18/RPAH/603), South Eastern Sydney Local Health District (NSW, 19/G/028, 18/G/338), Nepean Blue Mountains Local Health District (NSW, SSA/19/NEPAN/11), Northern Sydney Local Health District (NSW, RESP/19/027, RESP/19/028), Metro North Hospital and Health Service (Queensland, HREC/18/RPAH/603), University of South Australia (SA – 201215), Calvary Health Care Adelaide (SA, 19-CHREC-F001), Southern Adelaide Local Health Network (SA, HREC/18/RPAH/603), Bendigo Health Victoria (SSA/48255/BHCG-2019), Barwon Health (18/246), Peninsula Health Victoria (SSA/48255/PH-2019), Western Health Victoria (48255), St John of God Health Care Victoria (1540), South Metropolitan Health Western Australia (WA – Western Australia, RGS0000001358), North Metropolitan Health (WA – RGS0000001358), Sir Charles Gardiner Hospital (WA – RGS0000001358) and Launceston General Hospital Tasmania (H0017903).

Consent for publication

Not applicable.

Availability of data and materials

The datasets during and/or analysed during the current study will be made available from the corresponding author on reasonable request

Competing interests

The authors declare that they have no competing interests

Funding

The study is being funded by a four-year Medical Research Futures Fund (MRFF – Australian Federal Government) grant. The funding was not provided with any conditions and the MRFF has no authority over the design of the study or collection, management, analysis or interpretation of data or the writing of manuscripts for submission. No industry funding or other sources of funding are being used for this trial.

Authors' contributions

All authors listed have contributed to this protocol and the ICMJE guidelines were consulted for determining authorship. VS, IAH, RB, SG, SA, JMN, RdS, NP, INA, ML, DB, KC and TLK were responsible for the planning of the trial, protocol development and writing, TLK and

NP were responsible for the description of the statistical analyses used. All authors reviewed the final version of this manuscript prior to submission.

Acknowledgements

Not applicable.

REFERENCES

1. Mirkazemi C, Bereznicki LR, Peterson GM. Comparing Australian orthopaedic surgeons' reported use of thromboprophylaxis following arthroplasty in 2012 and 2017. BMC Musculoskelet Disord. 2019; 20:57.
2. Adam SS, McDuffie JR, Lachiewicz PF, Ortel TL, Williams JW, Jr. Comparative effectiveness of new oral anticoagulants and standard thromboprophylaxis in patients having total hip or knee replacement: a systematic review. Ann Intern Med. 2013; 159:275-284.
3. Gomez-Outes A, Terleira-Fernandez AI, Suarez-Gea ML, Vargas-Castrillon E. Dabigatran, rivaroxaban, or apixaban versus enoxaparin for thromboprophylaxis after total hip or knee replacement: systematic review, meta-analysis, and indirect treatment comparisons. BMJ. 2012; 344:e3675.
4. Cao YB, Zhang JD, Shen H, Jiang YY. Rivaroxaban versus enoxaparin for thromboprophylaxis after total hip or knee arthroplasty: a meta-analysis of randomized controlled trials. Eur J Clin Pharmacol. 2010; 66:1099-1108.
5. Cohen A, Drost P, Marchant N, Mitchell S, Orme M, Rublee D, et al. The efficacy and safety of pharmacological prophylaxis of venous thromboembolism following elective knee or hip replacement: systematic review and network meta-analysis. Clin Appl Thromb Hemost. 2012; 18:611-627.
6. Harenberg J, Marx S, Dahl OE, Marder VJ, Schulze A, Wehling M, et al. Interpretation of endpoints in a network meta-analysis of new oral anticoagulants following total hip or total knee replacement surgery. Thromb Haemost. 2012; 108:903-912.
7. Neumann I, Rada G, Claro JC, Carrasco-Labra A, Thorlund K, Akl EA, et al. Oral direct Factor Xa inhibitors versus low-molecular-weight heparin to prevent venous thromboembolism in patients undergoing total hip or knee replacement: a systematic review and meta-analysis. Ann Intern Med. 2012; 156:710-719.
8. Turun S, Banghua L, Yuan Y, Zhenhui L, Ying N, Jin C. A systematic review of rivaroxaban versus enoxaparin in the prevention of venous thromboembolism after hip or knee replacement. Thromb Res. 2011; 127:525-534.
9. Bozic KJ, Vail TP, Pekow PS, Maselli JH, Lindenauer PK, Auerbach AD. Does aspirin have a role in venous thromboembolism prophylaxis in total knee arthroplasty patients? J Arthroplasty. 2010; 25:1053-1060.
10. Jameson SS, Baker PN, Charman SC, Deehan DJ, Reed MR, Gregg PJ, et al. The effect of aspirin and low-molecular-weight heparin on venous thromboembolism after knee replacement: a non-randomised comparison using National Joint Registry Data. J Bone Joint Surg Br. 2012; 94:914-918.
11. Jameson SS, Charman SC, Gregg PJ, Reed MR, van der Meulen JH. The effect of aspirin and low-molecular-weight heparin on venous thromboembolism after hip replacement: a non-randomised comparison from information in the National Joint Registry. J Bone Joint Surg Br. 2011; 93:1465-1470.
12. Anderson DR, Dunbar M, Murnaghan J, Kahn SR, Gross P, Forsythe M, et al. Aspirin or Rivaroxaban for VTE Prophylaxis after Hip or Knee Arthroplasty. N Engl J Med. 2018; 378:699-707.
13. Anderson DR, Dunbar MJ, Kahn SR. Aspirin versus low-molecular-weight heparin after total hip arthroplasty. Ann Intern Med. 2013; 159:502-503.

14. Sidhu VS, Graves SE, Buchbinder R, Naylor JM, Pratt NL, de Steiger RS, et al. CRISTAL: protocol for a cluster randomised, crossover, non-inferiority trial of aspirin compared to low molecular weight heparin for venous thromboembolism prophylaxis in hip or knee arthroplasty, a registry nested study. BMJ Open. 2019; 9:e031657.
15. [https://www.sealedenvelope.com]
16. Bjornara BT, Gudmundsen TE, Dahl OE. Frequency and timing of clinical venous thromboembolism after major joint surgery. J Bone Joint Surg Br. 2006; 88:386-391.
17. Kulshrestha V, Kumar S. DVT prophylaxis after TKA: routine anticoagulation vs risk screening approach - a randomized study. J Arthroplasty. 2013; 28:1868-1873.
18. Bawa H, Weick JW, Dirschl DR, Luu HH. Trends in Deep Vein Thrombosis Prophylaxis and Deep Vein Thrombosis Rates After Total Hip and Knee Arthroplasty. J Am Acad Orthop Surg. 2018; 26:698-705.
19. Giraudeau B, Ravaud P, Donner A. Sample size calculation for cluster randomized cross-over trials. Stat Med. 2008; 27:5578-5585.
20. Kelly T-L, Pratt N. A note on sample size calculations for cluster randomised crossover trials with a fixed number of clusters. Stat Med. 2019; 38:3342-3345.
21. AOA. Australian Orthopaedic Association National Joint Replacement Registry Annual Report. 2019.
22. Peto R, Pike MC, Armitage P, Breslow NE, Cox DR, Howard SV, et al. Design and analysis of randomized clinical trials requiring prolonged observation of each patient. I. Introduction and design. Br J Cancer. 1976; 34:585-612.
23. Haybittle JL. Repeated assessment of results in clinical trials of cancer treatment. Br J Radiol. 1971; 44:793-797.
24. White IR, Daniel R, Royston P. Avoiding bias due to perfect prediction in multiple imputation of incomplete categorical variables. Comput Stat Data Anal. 2010; 54:2267-2275.
25. Rodwell L, Lee KJ, Romaniuk H, Carlin JB. Comparison of methods for imputing limited-range variables: a simulation study. BMC Med Res Methodol. 2014; 14:57.
26. Forbes AB, Akram M, Pilcher D, Cooper J, Bellomo R. Cluster randomised crossover trials with binary data and unbalanced cluster sizes: application to studies of near-universal interventions in intensive care. Clin Trials. 2015; 12:34-44.
27. Turner RM, White IR, Croudace T, Group PIPS. Analysis of cluster randomized cross-over trial data: a comparison of methods. Stat Med. 2007; 26:274-289.
28. Schumi J, Wittes JT. Through the looking glass: understanding non-inferiority. Trials. 2011; 12:106.

STATISTICAL ANALYSIS PLAN (FINAL): 20 July 2021

TITLE PAGE

Study Title

CRISTAL (A cluster-randomised, crossover, non-inferiority trial of aspirin compared to low molecular weight heparin for venous thromboembolism prophylaxis in hip or knee arthroplasty, a registry nested study): statistical analysis plan

Corresponding Author:
Dr Verinder Singh Sidhu (author)
Postal Address: Whitlam Orthopaedic Research Centre, Level 1, The Ingham Institute for Applied Medical Research, 1 Campbell Street, Liverpool, NSW, 2170
Email address: Verinder.s.sidhu@gmail.com
Phone: +61 422 167 435
Fax Number: +61 2 8738 3850

Co-Authors

14. Dr Thu-Lan Kelly (senior statistician)
    Affiliation: Clinical and Health Sciences, Quality Use of Medicines Pharmacy Research Centre, University of South Australia, Adelaide, South Australia, Australia
15. Dr Nicole Pratt (senior statistician)
    Affiliation: Clinical and Health Sciences, Quality Use of Medicines Pharmacy Research Centre, University of South Australia, Adelaide, South Australia, Australia
16. Professor Steven Graves
    Affiliation: Australian Orthopaedic Association National Joint Replacement Registry, Adelaide, South Australia, Australia
17. Professor Rachelle Buchbinder
    Affiliations:
    iii. Department of Epidemiology and Preventive Medicine, Monash University School of Public Health, Melbourne, Victoria, Australia
    iv. Monash Department of Clinical Epidemiology, Cabrini Institute, Melbourne, Victoria, Australia
18. Associate Professor Justine Naylor
    Affiliation: Whitlam Orthopaedic Research Centre, Ingham Institute for Applied Medical Research, South West Sydney Clinical School, The University of New South Wales Sydney, Sydney, NSW, Australia
19. Professor Richard de Steiger
    Affiliation: Department of Surgery, Epworth Healthcare, University of Melbourne, Melbourne, Victoria, Australia
20. Associate Professor Ilana Ackerman
    Affiliation: Department of Epidemiology and Preventive Medicine, Monash University School of Public Health and Preventive Medicine, Melbourne, Victoria, Australia
21. Associate Professor Sam Adie
    Affiliation: St. George and Sutherland Clinical School, The University of New South Wales Sydney, Sydney, NSW, Australia

22. Ms Michelle Lorimer
Affiliation: South Australian Health and Medical Research Institute, Adelaide, South Australia, Australia
23. Mrs Durga Bastiras
Affiliation: Australian Orthopaedic Association National Joint Replacement Registry, Adelaide, South Australia, Australia
24. Ms Kara Cashman
Affiliation: South Australian Health and Medical Research Institute, Adelaide, South Australia, Australia
25. Professor Ian Harris (chief investigator)
Affiliations:
iii. Whitlam Orthopaedic Research Centre, Ingham Institute for Applied Medical Research, South West Sydney Clinical School, The University of New South Wales Sydney, Sydney, NSW, Australia
iv. Institute of Musculoskeletal Health, School of Public Health, The University of Sydney, Sydney, NSW, Australia

ABSTRACT

Background:
This *a priori* statistical analysis plan describes the analysis for CRISTAL.

Methods:
CRISTAL (cluster-randomised, crossover, non-inferiority trial of aspirin compared to low molecular weight heparin for venous thromboembolism prophylaxis in hip or knee arthroplasty, a registry nested study) aims to determine whether aspirin is non-inferior to low molecular weight heparin (LMWH) in preventing symptomatic venous thromboembolism (VTE) following hip arthroplasty (HA) or knee arthroplasty (KA). The study is nested within the Australian Orthopaedic Association National Joint Replacement Registry. The trial was commenced in April 2019 and after an unplanned interim analysis, recruitment was stopped (December 2020), as the stopping rule was met for the primary outcome.

The clusters comprised hospitals performing > 250 HA and/or KA procedures per annum, whereby all adults (> 18 years) undergoing HA or KA were recruited. Each hospital was randomised to commence with aspirin, orally, 85-150mg daily or LMWH (enoxaparin), 40mg, subcutaneously, daily within 24 hours postoperatively, for 35 days after HA and 14 days after KA. Crossover was planned once the registration target was met for the first arm.

The primary end point is symptomatic VTE within 90 days. Secondary outcomes include readmission, reoperation, major bleeding and death within 90 days, and reoperation and patient-reported pain, function and health status at 6 months.

The main analyses will focus on the primary and secondary outcomes for patients undergoing elective primary total HA and KA for osteoarthritis. The analysis will use an intention-to-treat approach with cluster summary methods to compare treatment arms. As the trial stopped early, analyses will account for incomplete cluster crossover and unequal cluster sizes.

Conclusions:
This paper provides a detailed statistical analysis plan for CRISTAL.

Trial registration: Australian and New Zealand Clinical Trials Registry, ID: ACTRN12618001879257. Registered on 19/11/2018.

Key Words
Venous Thromboembolism, Hip Arthroplasty, Knee Arthroplasty, Aspirin, Low Molecular Weight Heparin, Statistical Analysis Plan

MANUSCRIPT

Background

Despite the increasing use of aspirin as a sole chemotherapeutic agent for symptomatic venous thromboembolic event (VTE) prophylaxis following hip arthroplasty (HA) and knee arthroplasty (KA) [1], there remains limited high quality comparative evidence for its safety and efficacy. The majority of studies supporting the safety and efficacy of aspirin compared to other agents, including low molecular weight heparin (LMWH), have been retrospective or non-randomised [2-11]. The only randomised trials have been underpowered or have used an alternative form of prophylaxis (e.g., LMWH or a novel oral anticoagulant (NOAC)) for the immediate postoperative period following HA or KA prior to changing to aspirin for extended prophylaxis, which does not reflect the way aspirin is used in Australia [12, 13]. CRISTAL is a pragmatic, multicentre cluster-randomised, two period cross-sectional crossover trial that aims to determine if aspirin is non-inferior to LMWH in the prevention of symptomatic VTE following HA and KA. It is nested within the Australian Orthopaedic Association National Joint Replacement Registry (AOANJRR).

The trial commenced in April 2019 and the estimated timeline for completion of patient registration was 24 months. However, after an unplanned interim analysis in which the trial stopping rule was met, patient registration was ceased in December 2020, resulting in incomplete crossover. This statistical analysis plan details the planned analyses for CRISTAL to facilitate transparency of data analysis. The CONSORT statement for cluster randomised trials was referred to in preparation of this document [14]. The trial protocol has previously been published [15].

STUDY OVERVIEW

Ethics

Ethics approval was granted from all relevant central, lead ethics committees involved and all participating hospitals, as outlined in the published trial protocol [15]. The trial is registered with the Australian New Zealand Clinical Trials Registry (ACTRN12618001879257p) and is endorsed by the Australia and New Zealand Musculoskeletal (ANZMUSC) Clinical Trials Network.

Participating Hospitals and Patient Registration

The clusters in CRISTAL were defined as hospitals where hip and knee arthroplasty procedures were performed. Hospitals were eligible for recruitment provided they agreed to follow the trial protocol and if they performed greater than 250 HA and/or KA procedures per annum. There were 31 hospitals (clusters) that were recruited.

Each recruited hospital was responsible for registering patients and complying with the trial protocol. The AOANJRR routinely collects data pertaining to the procedure, patient age, sex, American Society of Anaesthesiologists (ASA) class and body mass index (BMI) and death on all patients undergoing HA and KA procedures. Patient-reported outcomes are collected through the electronic Clinical Trials Platform, which requires pre-operative registration of the patient onto the electronic system. All adult (age 18 and older) patients undergoing HA or KA were eligible for registration into the study and eligible to receive the allocated study drug, except for those who were already on long-term anticoagulation (specifically a NOAC,

warfarin or dual antiplatelet therapy (DAPT)) and those with a medical contraindication to either drug, e.g., an allergy or a medical comorbidity such as thrombophilia that precluded treatment with the study drug.

Patients who were not registered in the electronic Clinical Trials Platform will be included in secondary analyses, as procedure information, demographics and mortality were still recorded even though the primary outcome and other patient-reported outcomes were not recorded.

Intervention

Each hospital (cluster) was allocated to consecutive periods of a standard protocol of LMWH and a standard protocol of aspirin as VTE prophylaxis, with the order being randomised. Patients in the aspirin group received aspirin at 85-150mg once daily, orally for 35 days post HA and for 14 days post KA, commencing within 24 hours of surgery. Patients in the LMWH group received enoxaparin at 40mg once daily, subcutaneously for the same time periods, with this dose reduced to 20mg for patients who weigh less than 50kg and for patients with an estimated glomerular filtration rate (eGFR) of less than 30mL/min who are not on dialysis. Other interventions that were standard across all sites were the intra- and post-operative use of intermittent pneumatic compression (IPC) calf devices until patients are mobile, the use of compression stockings, and mobilisation offered on day 0 or day 1 postoperatively.

Randomisation and allocation

Study investigators have remained blinded to group allocation. All 31 participating hospitals were randomised to commence with either LMWH or aspirin, in randomly permuted blocks of size four by statisticians from the South Australian Health and Medical Research Institute (SAHMRI), independent of study investigators. The randomisation sequence was generated using an online application [16] and this was provided to an unblinded data manager from SAHMRI. The hospital was then allocated to a treatment sequence by SAHMRI staff and this information was provided to the AOANJRR (independent of study investigators), with the site being informed of their allocated treatment arm the week prior to commencing initial patient registration. Hospitals followed the designed protocol for patients for their allocated treatment arm and were advised to crossover to the alternate treatment once the sample size for the first treatment arm was met.

For clusters who did not reach the sample size for the first arm within 18 months of commencement, crossover occurred prior to reaching the sample size so that an equal number of patients could be registered in each arm within the study timeframe.

Evaluation of adherence to the study protocol and protocol deviations

At a hospital level, during the course of the trial each hospital was audited within the first month of each treatment arm to ensure they were complying with the trial protocol and to ensure each cluster received the intended allocated treatment. The audit consisted of the first 20 patients of each treatment arm. If a site had a compliance of less than 80%, the site was educated on methods of improving protocol compliance and subsequently re-audited until compliance to the protocol was above 80%.

Hospitals were also advised to inform trial co-ordinators of patients not receiving the correct study drug or those patients who had the study drug withheld for greater than 48 hours due to side effects (e.g. allergy, excessive wound drainage or bleeding events). These protocol deviations were recorded using the Clinical Trials Platform.

Outcome variables

The primary outcome of the study is symptomatic VTE within 90 days of surgery. Secondary outcomes are:

- Deep vein thrombosis (DVT) only (total, below-knee and above-knee) within 90 days
- Pulmonary embolism (PE) only within 90 days
- Readmission related to the original surgery or associated treatment (including bleeding and VTE-related) within 90 days
- Reoperation on the same joint within 90 days and within 6 months of surgery
- Major bleeding events within 90 days defined as bleeding events resulting in readmission, reoperation or death
- Death within 90 days
- Change in patient-reported pain, function and health status measures as measured by the Oxford Hip Score (OHS), Oxford Knee Score (OKS), EQ-5D score and the EQ-VAS from baseline to 6 months postoperatively

Outcome and demographic data were collected preoperatively (demographics, patient reported pain, function and health status) and at 90 days and 6 months postoperatively. Data for all primary and secondary outcomes are patient-reported (except for death). All patients who responded 'yes' to having experienced a VTE or a secondary operation within 6 months had this result verified by AOANJRR staff through contact with treating doctors and hospitals. A random audit of 200 patients who did not report a VTE event was undertaken to detect the false negative reporting rate. All data collected for registered patients specific to CRISTAL have been outlined in the published protocol [15]. Mortality data were collected through linkage between the AOANJRR and the National Death Index.

In the published protocol [15], mortality was to be measured at 90 days and 6 months. Due to the lack of sensitivity in measuring VTE-related mortality at 6 months, and due to the lag in data availability for mortality, we will only analyse mortality at 90 days [17].

Power and sample size

For the sample size calculation in CRISTAL, we used an estimated overall event rate of 2% (based on the current available literature) [18, 19], a non-inferiority margin of 1% (based on clinician opinion and a recent randomised controlled trial) [12], i.e., an event rate of 2.5% for aspirin and 1.5% for LMWH, a power of 90% and a one-sided significance level of 0.025. For an individual randomised trial, this yields a sample size of 4,117 per treatment group or a total of 8,234 patients. For a cluster-randomised crossover trial with an intracluster correlation of 0.01, an interperiod correlation of 0.008 and 31 clusters, the required sample

size is 11,160 patients, or 180 patients per arm for each cluster [20, 21]. However, due to the uncertainty surrounding the event rate and intracluster and interperiod correlations, loss to follow-up, uneven recruitment rates leading to unequal cluster sizes or clusters dropping out of the study, we aimed to register 251 patients eligible for the primary objective of the study, providing a total of 15,562 patients. This figure allowed for a maximum 27% reduction in the above sample size calculation [15], however, actual loss to follow-up was expected to be less than this.

STATISTICAL ANALYSIS PLAN

Patient Populations and Subgroups for Analyses

The total patient population for CRISTAL comprises all patients undergoing HA or KA at participating institutions over the duration of the study, regardless of whether these patients were registered or eligible to receive the study drug (defined as population 5, see Figure 1).

Within this total population, the following populations will be used to form the basis of the analyses:

- Registered patients undergoing any form of HA or KA (including partial or revision surgery, for any indication) regardless of eligibility to receive the study drug (population 4)
- Registered patients undergoing any form of HA or KA (including partial or revision surgery, for any indication) who were eligible to receive the study drug (population 3)
- Registered patients undergoing elective primary THA or TKA (for any indication) who were eligible to receive the study drug (population 2)
- All registered patients undergoing elective primary THA or TKA for a recorded diagnosis of osteoarthritis (OA) who were eligible to receive the study drug (population 1)

These populations are represented diagrammatically in Figure 1.

Figure 1. Patient populations within CRISTAL

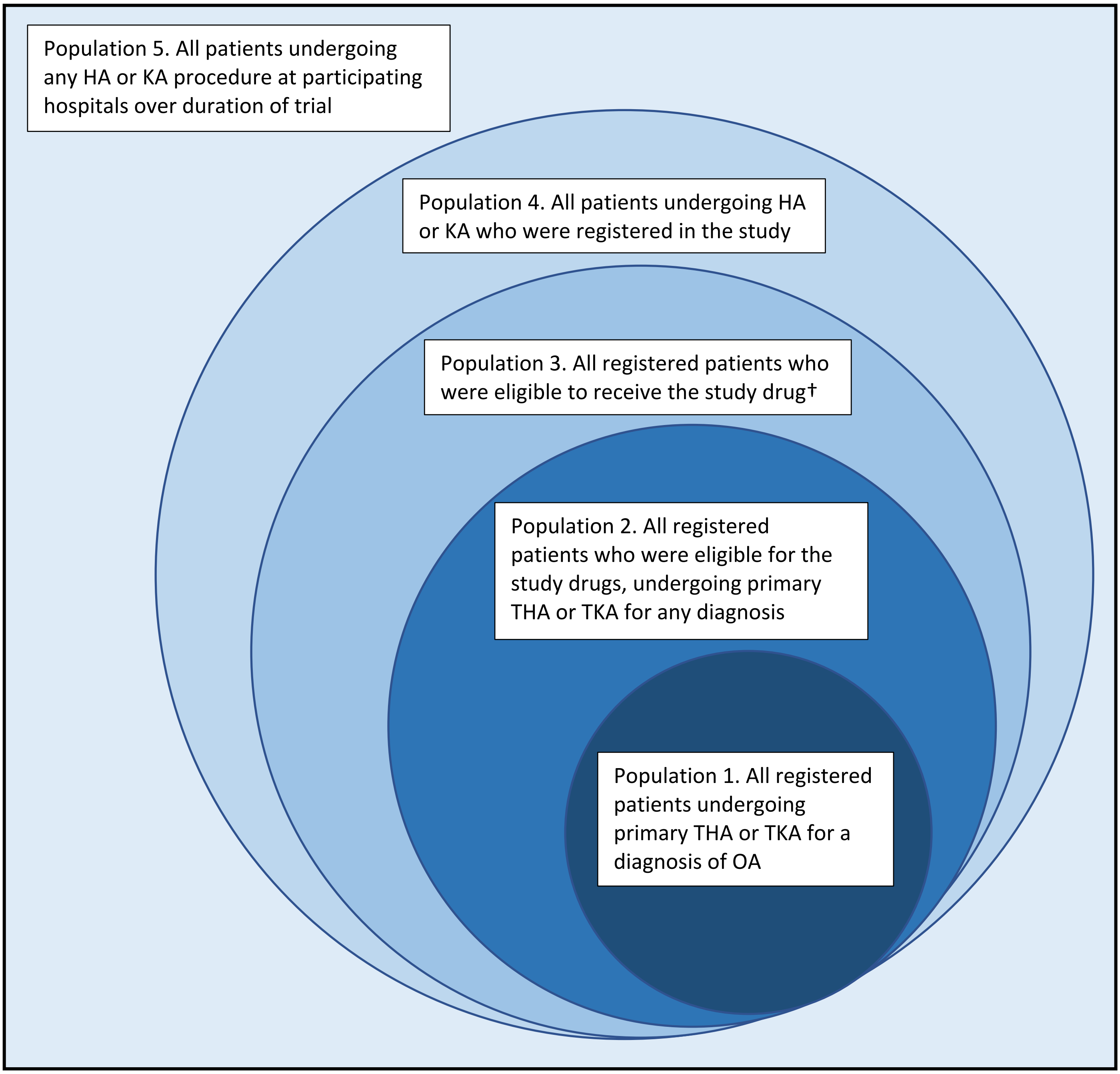

Legend:

Abbreviations: *HA* hip arthroplasty, *KA* knee arthroplasty, *THA* total hip arthroplasty, *TKA* total knee arthroplasty, *OA* osteoarthritis

† Study drug excluded for patients who already on long-term anticoagulation (specifically a novel oral anticoagulant – NOAC, warfarin or dual antiplatelet therapy – DAPT) and those who have a medical contraindication

Each outcome (primary and secondary) will be assessed for populations 1, 2, 3 and 4 listed in Figure 1. Mortality will be assessed for all populations (including population 5). The primary objective of the study as outlined in the published protocol [15], was the analysis of population 1 only (registered patients undergoing primary THA or TKA for a diagnosis of OA who are eligible to receive the study drug), as this was the focus of the sample size calculation. This population will remain the focus of the main analyses.

Population 1 was chosen as the focus of the main analysis as these patients represent the majority of patients undergoing HA or KA procedures and there are known differences in outcomes and co-morbidities with other diagnoses (e.g., fracture, tumour), which could confound the primary outcome [22].

For the primary end point of VTE, the following subgroup analyses will be conducted within the corresponding populations listed:

- Type of joint replacement: primary THA compared to primary TKA – population 1
- Bilateral arthroplasty: patients undergoing simultaneous bilateral arthroplasty compared to those who are not – population 1
- Revision arthroplasty: patients undergoing revision hip or knee arthroplasty compared to those undergoing primary arthroplasty – population 3
- Prior history of VTE: patients with a prior history of VTE compared to those without – population 1

Analysis principles

Data will be analysed according to the intention-to-treat principle with clusters analysed according to assigned group allocation. Although hospital and patient protocol deviations will be recorded, no as-treated analyses will be performed, as there are no verified data available to determine whether individual patients received the assigned study drug for the full period, given the pragmatic nature of the trial. The timing of analyses will be stratified by follow-up time of the outcomes measured (90 days and 6 months). The difference in absolute risk for symptomatic VTE between each group and 95% confidence intervals (upper and lower) will be examined to determine if the non-inferiority margin is met.

Continuous variables will be summarised using standard measures of central tendency and dispersion, using either mean and standard deviation or median and interquartile range. Categorical variables will be summarised by frequencies and percentages.

Analyses will be performed using SAS version 9.4 (SAS Institute, Cary USA) and R (R Foundation for Statistical Computing Platform) version 4.0.2 or higher.

Interim analysis

An interim analysis was not initially planned, as both treatments are considered standard practice for VTE prophylaxis in Australia and the trial is investigating an adverse event as the primary outcome. However, due to concerns of an increased adverse event rate (symptomatic VTE and death) in one of the prophylaxis groups, a Data Safety Monitoring

Board (DSMB) was convened one year into patient recruitment. The DSMB consisted of an orthopaedic surgeon, a haematologist and a statistician, all independent of the trial.

The DSMB were advised by the Trial Management Committee (TMC) to conduct an interim analysis. In conjunction with the DSMB (prior to the interim analysis), the TMC applied the Haybittle-Peto stopping rule of a two-sided significance of 0.001 for the primary outcome in the population 1 [23, 24]. This stopping rule was chosen as it does not require adjustment of the significance threshold for the final analysis and allows further interim analyses using the same threshold (if required).

After the first interim analysis (in September 2020), the DSMB recommended continuing the trial and performing a second interim analysis in November 2020. After reviewing the second interim analysis, the DSMB recommended ceasing patient recruitment as the stopping rule had been met. The study ceased recruiting patients in December 2020 and sites reverted to their usual VTE prophylaxis pathways.

Methods used for Interim Analyses
Interim analyses were conducted for VTE and mortality within 90 days for population 1. To account for unequal cluster sizes, incomplete crossover or clusters which had not yet crossed over, a composite analysis was designed. For clusters which had crossed over, including with partial completion of the second period, the cluster weighted estimator intended for the primary outcome was used. Harmonic mean weighting when there are unequal cluster sizes has been shown to improve precision and 95% confidence interval coverage compared with unweighted or inverse variance estimates [25, 26]. Clusters which had not crossed over were analysed using the cluster period summaries, weighted by cluster size, in a parallel design approach, i.e., as if it were a cluster randomised trial without crossover. Estimates for the two approaches were combined using inverse variance weights to provide a final estimate. Confidence intervals were constructed using the Haybittle-Peto boundary of 0.001.

Data integrity
Integrity of data will be checked prior to conducting the final analysis. The data set will be checked for errors, omissions and double data entry. These will be resolved prior to commencing the analysis in consultation with the data management plan [15].

Blinding
The DSMB were blinded to treatment allocation (groups in the interim analyses were labelled A and B). All researchers involved in the preparation of this analysis plan will have no access to trial data broken down by treatment allocation for the final statistical analysis. Once data integrity checks have been conducted, a blind review to quantify missing data of the entire dataset will be conducted and any final amendments to the statistical analysis plan will be made before the database is locked. During analysis and interpretation, group allocation will be masked by dummy group names and the true allocation will be unmasked only after the final statistical report has been completed and interpretation has been agreed to by the writing group and minuted.

Methods for handling missing data
Multiple imputation using chained equations will be used to account for missing data. The imputation model will use auxiliary variables gathered from routine AOANJRR data (including age, sex, baseline health, pain and function, diagnosis and surgical factors), as well as cluster and period effects. One hundred datasets will be imputed at the patient level, then each dataset will be analysed using the main analysis method with cluster summaries and combined using Rubin's rules. If there is any possibility of bias due to perfect prediction of rare outcomes such as VTE [27] or imputing values out of range for bounded variables such as pain scores or EQ5D [28], multiple imputation using chained equations will not be performed. Since the most likely reason for loss to follow-up is difficulty in contacting patients postoperatively (rather than association with treatment assignment or outcome), missing data will be assumed to be missing at random.

As a further sensitivity analysis for the primary outcome only, inverse probability weighting, where the complete cases are weighted by the inverse probability of being complete case will also be used to account for missing data. Inverse probability weighting has an advantage over multiple imputation when there are large blocks of missing data with either observed values for all variables or missing values for the majority of the variables, for example, pre-operative pain and function scores [29]. The inverse probability weights will be used to produce weighted cluster summaries, which will be analysed using the main analysis method, with cluster sizes calculated as the sum of the inverse probability weights.

Trial profile and baseline characteristics
The flow of participating hospitals (including losses and exclusions) through the study and participating patients will be reported in line with the Consolidated Standards of Reporting Trials (CONSORT) statement (Figures 2 and 3).

Baseline characteristics of participating clusters, including number of annual HA and KA procedures performed in the year prior to trial commencement, hospital type (public or private hospital), initial treatment allocation and whether the hospital achieved crossover are shown in Table 1. This table also shows the number of patients registered for population 1 (the population used for the sample size calculation) by each participating hospital and the overall registration rate of each hospital will be presented as outlined. The overall registration rate describes the number of registered patients undergoing any HA or KA procedure (population 4) divided by the number of patients who underwent any HA or KA procedure over the duration of the trial at participating hospitals (regardless of whether they were registered – population 5). Hospital names will remain anonymous.

Descriptive statistics of baseline patient characteristics for all registered patients eligible to receive the study drug (population 3) will be presented by prophylaxis group (Table 2).

Figure 2. Flowsheet of participating hospitals

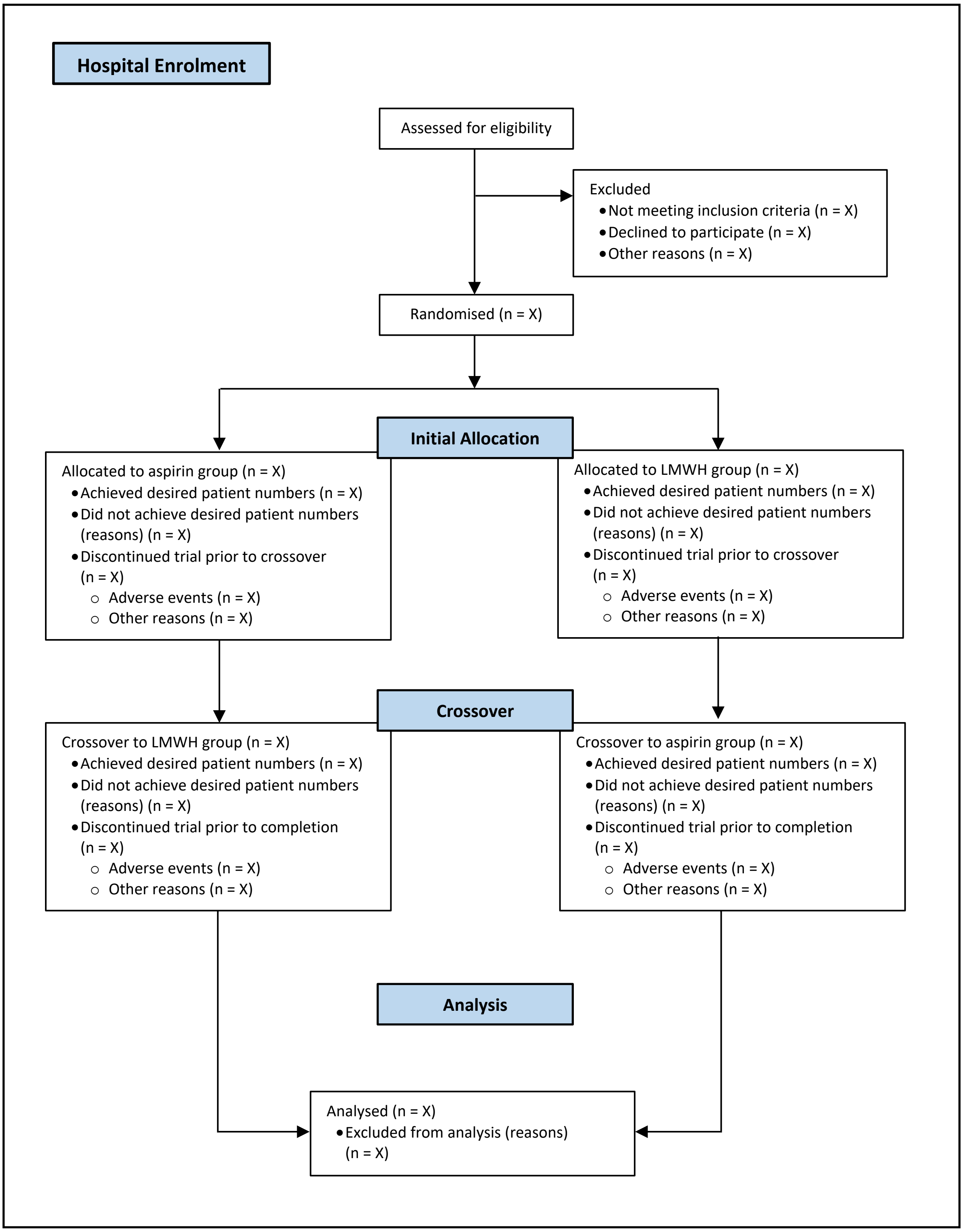

Figure 3. Flowsheet of patients within population 1[†]

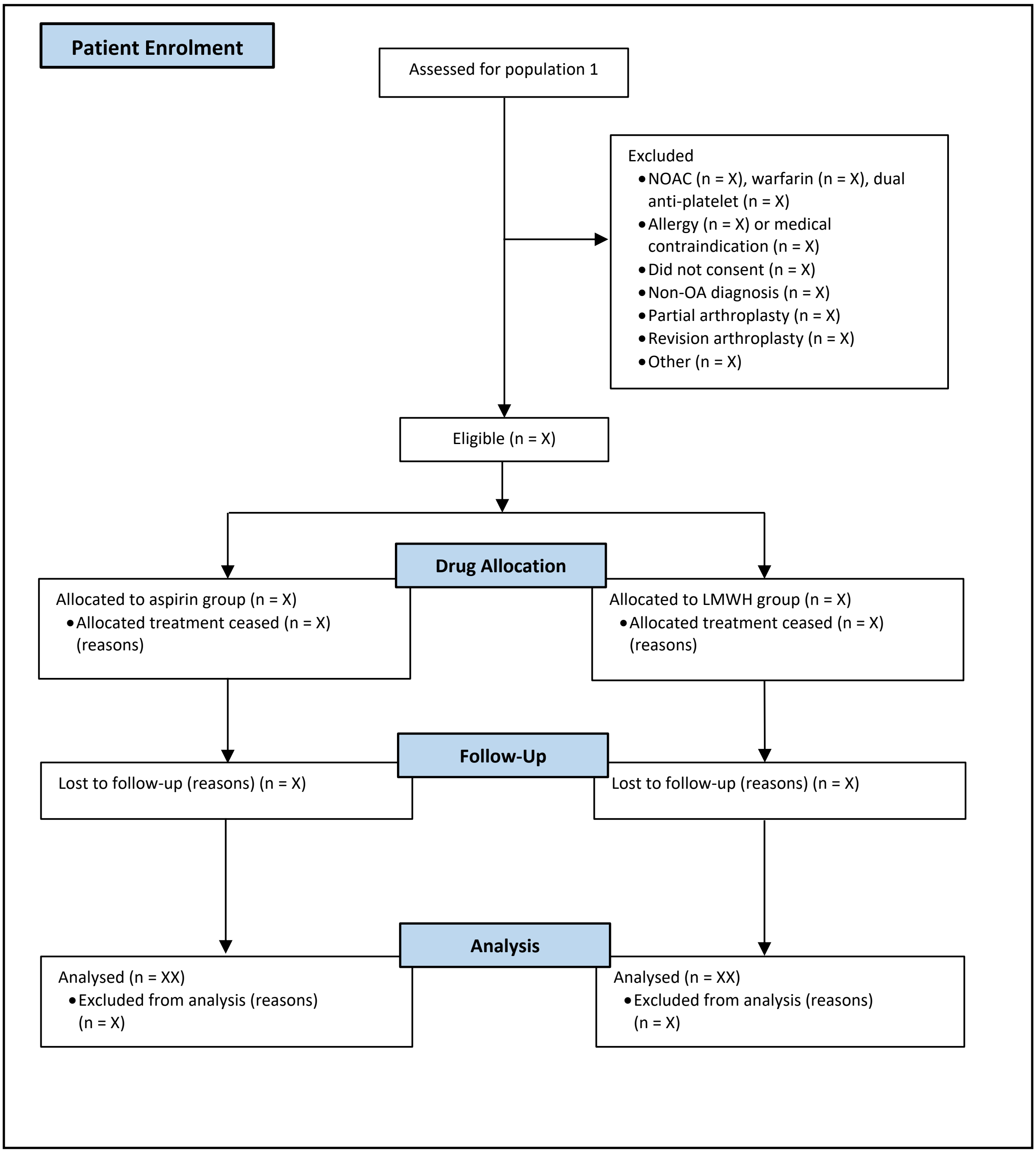

[†] Population 1 refers to registered patients undergoing primary THA or TKA for a diagnosis of OA, who are eligible to receive the study drug

Table 1. Number of registered patients for population 1† by treatment group and overall registration rate for each participating hospital

Hospital	Number of HA and KA Procedures performed (2018)	Insurance Status	Initial Treatment Allocation	Crossover Achieved	LMWH Group (Population 1)	Aspirin Group (Population 1)	Overall Registration Rate (combined for both groups)
1					n	n	%
2					n	n	%
3					n	n	%
4					n	n	%
5					n	n	%
6					n	n	%
7					n	n	%
8					n	n	%
9					n	n	%
10					n	n	%
11					n	n	%
12					n	n	%
13					n	n	%
14					n	n	%
15					n	n	%
16					n	n	%
17					n	n	%
18					n	n	%
19					n	n	%
20					n	n	%
21					n	n	%
22					n	n	%
23					n	n	%
24					n	n	%
25					n	n	%
26					n	n	%
27					n	n	%
28					n	n	%
29					n	n	%
30					n	n	%
31					n	n	%
**Total**					**n**	**n**	**%**

† Population 1 refers to registered patients undergoing primary THA or TKA for a diagnosis of OA, who are eligible to receive the study drug

Table 2. Baseline patient characteristics for all registered patients eligible to receive study drug (population 3), according to treatment allocation

	**LMWH** (n = X)	**Aspirin** (n = X)
Age (years)	xx.x (xx.x – xx.x), n	xx.x (xx.x – xx.x), n
BMI (kg/m^2)	xx.x (xx.x – xx.x), n	xx.x (xx.x – xx.x), n
Male sex	n (%)	n (%)
ASA Grading		
1	n (%)	n (%)
2	n (%)	n (%)
3	n (%)	n (%)
4	n (%)	n (%)
5	n (%)	n (%)
Previous venous thromboembolism	n (%)	n (%)
Long term anticoagulant use		
Aspirin	n (%)	n (%)
Other single antiplatelet	n (%)	n (%)
Joint replacement		
THA	n (%)	n (%)
TKA	n (%)	n (%)
Other HA	n (%)	n (%)
Other KA	n (%)	n (%)
Bilateral	n (%)	n (%)
Type of surgery		
Primary total	n (%)	n (%)
Primary partial	n (%)	n (%)
Primary resurfacing	n (%)	n (%)
Revision	n (%)	n (%)
Other	n (%)	n (%)
Indication		
Osteoarthritis	n (%)	n (%)
Inflammatory	n (%)	n (%)
Avascular Necrosis	n (%)	n (%)
Fracture	n (%)	n (%)
Other	n (%)	n (%)
Prosthesis		
Cemented	n (%)	n (%)
Hybrid	n (%)	n (%)
Uncemented	n (%)	n (%)
Pain and Function		
Oxford Hip Score	xx.x (xx.x – xx.x), n	xx.x (xx.x – xx.x), n
Oxford Knee Score	xx.x (xx.x – xx.x), n	xx.x (xx.x – xx.x), n
EQ-5D	xx.x (xx.x – xx.x), n	xx.x (xx.x – xx.x), n
EQ-VAS	xx.x (xx.x – xx.x), n	xx.x (xx.x – xx.x), n

Abbreviations: *BMI* body mass index, *ASA* American society of anaesthesiologists

MAIN ANALYSES

The main analyses will include the primary and secondary outcomes for registered patients eligible to receive the study drug undergoing THA or TKA for a diagnosis of OA (population 1). In addition, the primary and secondary outcomes will be analysed for populations 2, 3 and 4. Mortality will also be analysed for population 5 (see “Additional analyses” below).

For the primary outcome, the analysis will test the between-group difference of cases developing a symptomatic VTE within 90 days for non-inferiority of aspirin at a margin of 1%. Cluster summary methods will be used to estimate the treatment effect using cluster level differences. These have been shown to be appropriate for cluster-randomised crossover trials with rare outcomes, and the intracluster and interperiod correlation coefficients expected in this trial. The crossover difference per cluster is the mean outcome for the intervention period minus the mean outcome for the control period. In a linear regression of cluster differences on treatment sequence, the treatment effect estimate is the intercept. To account for potential unequal cluster sizes, a cluster size weighted estimator will be used with harmonic mean weights of the number of patients in the two periods, which was the same method used in the interim analyses for incomplete crossover [25, 26]. Treatment effects will be presented as absolute risk differences and 95% confidence intervals will be examined to determine whether the non-inferiority margin has been met and whether superiority of one drug can be concluded. The primary outcome will be presented in Table 3 and Figure 4 will be used to demonstrate whether the non-inferiority margin has been met for the population 1 [30].

The secondary outcomes will investigate non-VTE complications (death, re-operation, readmission and major bleeding events) within 90 days, and reoperation and patient-related pain and function at 6 months (OHS, OKS, EQ-5D and EQ-VAS). Cluster summary methods will be used within an intention-to treat approach. For binary outcomes, the cluster mean per period will be the proportion of patients who had the outcome, while for continuous outcomes such as pain score, the cluster mean will be the mean outcome. Treatment effects will be presented as absolute risk differences and 95% confidence intervals to determine if one treatment is superior to the alternative. Results for the secondary outcomes in population 1 will be presented in tabular form (Table 3) and results for the primary and secondary outcomes in populations 2, 3 and 4 in Table 4.

Due to the early stopping of the trial, final analyses of the primary and secondary outcomes will use the same composite method as the interim analyses which accounts for clusters with incomplete as well as no crossover, with 95% confidence intervals. No bias is expected from early stopping if the patients included in the trial are not systematically different from later patients who would have been included after the trial was stopped. Our composite analysis method accounts for clusters which either had incomplete crossover and or did not crossover. However, the lower sample size and unequal cluster sizes decreases the precision of the outcome estimates. Since we used cluster weighted estimates to account for unequal cluster sizes and increased the initial sample size by 27% above the minimum required, the loss of precision will be mitigated. The trial was stopped based on the Haybittle-Peto

boundary of 0.001, so we anticipate the final analysis using 95% confidence intervals will have sufficient power.

Table 3. Outcomes for population 1

Outcome	LMWH Allocation (n = X)	Aspirin Allocation (n = X)	Absolute Risk Difference	95% Confidence Interval	p-value
**Any Venous thromboembolism**	n (%)	n (%)	X	X – X	
**Type of Venous thromboembolism**					
Pulmonary embolism	n (%)	n (%)	X	X – X	
Deep venous thrombosis	n (%)	n (%)	X	X – X	
Both Pulmonary embolism and deep venous thrombosis	n (%)	n (%)	X	X – X	
Above knee deep venous thrombosis	n (%)	n (%)	X	X – X	
Below knee deep venous thrombosis	n (%)	n (%)	X	X – X	
Death	n (%)	n (%)	X	X – X	
Re-operation (90d)	n (%)	n (%)	X	X – X	
Reoperation (6 months)	n (%)	n (%)	X	X – X	
Re-admission	n (%)	n (%)	X	X – X	
Major Bleeding	n (%)	n (%)	X	X – X	
Pain and Function (median and IQR)†			X	X – X	
Oxford Hip Score	X (X – X)	X (X – X)	X	X – X	
Oxford Knee Score	X (X – X)	X (X – X)	X	X – X	
EQ-5D	X (X – X)	X (X – X)	X	X – X	
EQ-VAS	X (X – X)	X (X – X)			

† at 6 months

Figure 4. Between group change in overall 90-day symptomatic VTE rate and non-inferiority margin. The dotted line represents the non-inferiority margin

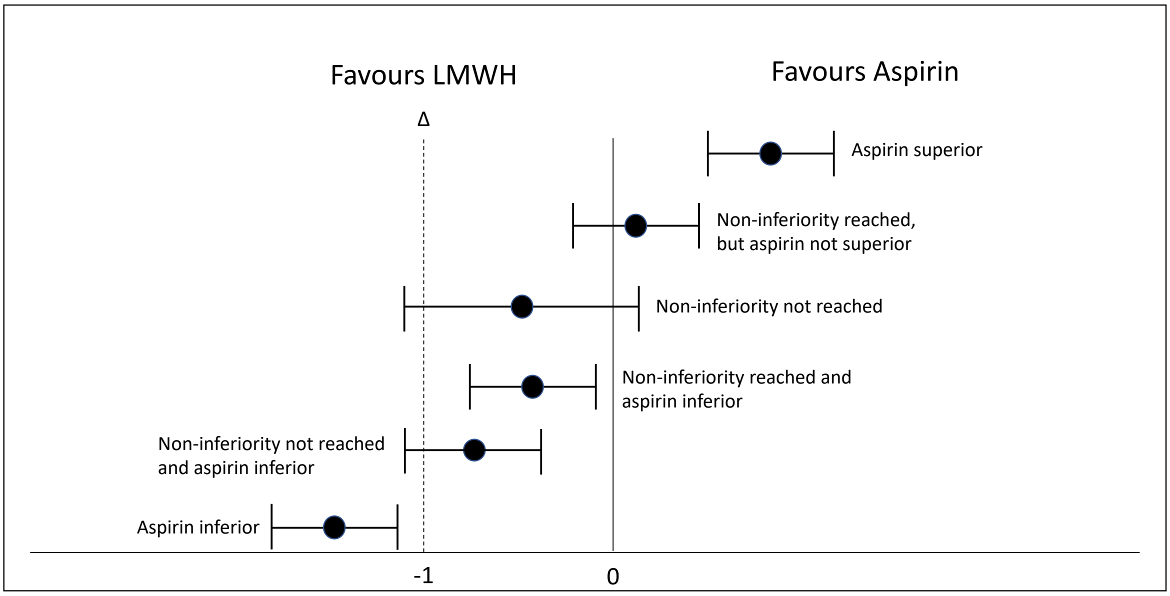

Table 4. Outcomes for populations 2, 3 and 4

Population	Outcome	LMWH Allocation (n = X)	Aspirin Allocation (n = X)	Absolute Risk Difference	95% Confidence Interval	p-value
All primary THA/TKA for any diagnosis eligible to receive study drug (population 2)	**Any Venous thromboembolism**	n (%)	n (%)	X	X – X	
	**Type of Venous thromboembolism**					
	Pulmonary embolism	n (%)	n (%)	X	X – X	
	Deep venous thrombosis	n (%)	n (%)	X	X – X	
	Both Pulmonary embolism and deep venous thrombosis	n (%)	n (%)	X	X – X	
	Above knee deep venous thrombosis	n (%)	n (%)	X	X – X	
	Below knee deep venous thrombosis	n (%)	n (%)	X	X – X	
	Death	n (%)	n (%)	X	X – X	
	Re-operation (90d)	n (%)	n (%)	X	X – X	
	Reoperation (6 months)	n (%)	n (%)	X	X – X	
	Re-admission	n (%)	n (%)	X	X – X	
	Major Bleeding	n (%)	n (%)	X	X – X	
	Pain and Function (median and IQR)†					
	Oxford Hip Score			X	X – X	
	Oxford Knee Score	X (X – X)	X (X – X)	X	X – X	
	EQ-5D	X (X – X)	X (X – X)	X	X – X	
	EQ-VAS	X (X – X)	X (X – X)	X	X – X	
		X (X – X)	X (X – X)			
All HA/KA eligible to receive study drug (population 3)	**Any Venous thromboembolism**	n (%)	n (%)	X	X – X	
	**Type of Venous thromboembolism**					
	Pulmonary embolism	n (%)	n (%)	X	X – X	
	Deep venous thrombosis	n (%)	n (%)	X	X – X	
	Both Pulmonary embolism and deep venous thrombosis	n (%)	n (%)	X	X – X	
	Above knee deep venous thrombosis	n (%)	n (%)	X	X – X	
	Below knee deep venous thrombosis	n (%)	n (%)	X	X – X	
	Death	n (%)	n (%)	X	X – X	
	Re-operation (90d)	n (%)	n (%)	X	X – X	
	Reoperation (6 months)	n (%)	n (%)	X	X – X	
	Re-admission	n (%)	n (%)	X	X – X	
	Major Bleeding	n (%)	n (%)	X	X – X	
	Pain and Function (median and IQR)†					
	Oxford Hip Score			X	X – X	
	Oxford Knee Score	X (X – X)	X (X – X)	X	X – X	
	EQ-5D	X (X – X)	X (X – X)	X	X – X	
	EQ-VAS	X (X – X)	X (X – X)	X	X – X	
		X (X – X)	X (X – X)			
All HA/KA including study drug exclusion (population 4)	**Any Venous thromboembolism**	n (%)	n (%)	X	X – X	
	**Type of Venous thromboembolism**					
	Pulmonary embolism	n (%)	n (%)	X	X – X	
	Deep venous thrombosis	n (%)	n (%)	X	X – X	
	Both Pulmonary embolism and deep venous thrombosis	n (%)	n (%)	X	X – X	
	Above knee deep venous thrombosis	n (%)	n (%)	X	X – X	
	Below knee deep venous thrombosis	n (%)	n (%)	X	X – X	
	Death	n (%)	n (%)	X	X – X	
	Re-operation (90d)	n (%)	n (%)	X	X – X	
	Reoperation (6 months)	n (%)	n (%)	X	X – X	
	Re-admission	n (%)	n (%)	X	X – X	
	Major Bleeding	n (%)	n (%)	X	X – X	
	Pain and Function (median and IQR)†					
	Oxford Hip Score			X	X – X	
	Oxford Knee Score	X (X – X)	X (X – X)	X	X – X	
	EQ-5D	X (X – X)	X (X – X)	X	X – X	
	EQ-VAS	X (X – X)	X (X – X)	X	X – X	
		X (X – X)	X (X – X)			

† at 6 months

Subgroup Analyses
Subgroup analyses for the primary outcome (treatment group differences by subgroup) will include THA or TKA, bilateral or unilateral procedures, a prior history of VTE or not for population 1, and primary arthroplasty or revision arthroplasty for population 3.

To assess treatment effects for each subgroup separately, cluster summaries will be produced for each subgroup. An interaction term between treatment group and subgroup (e.g., THA/TKA, bilateral/unilateral) will be added to the model for the primary outcome. The treatment differences for each subgroup will be assessed for non-inferiority. Since the trial was stopped early, the same composite method for the primary outcome and interim analyses will be used.

Sensitivity analyses
Sensitivity analyses will be performed to determine: (a) the effect of high-volume arthroplasty sites; (b) sites with high and low overall registration rates; (c) sites that required multiple compliance audits; and (d) the effect of patients who take long-term aspirin therapy on the results of the analyses for the primary outcome in population 1. The same methods for the main analyses will be used.

Order of planned analyses
Analyses will be performed in the following order:

- Interim analyses of population 1
- Primary and secondary outcomes for population 1
- Subgroup analyses for population 1
- Primary and secondary outcomes for populations 2, 3 and 4
- Subgroup analyses of population 3
- Sensitivity analyses in population 1

ADDITIONAL ANALYSES

Mortality Analysis

In addition to analysing between-group mortality for populations 1, 2, 3 and 4, the between-group 90-day mortality will be analysed for two further populations:

3. All patients undergoing HA or KA over the duration of the study at participating hospitals, regardless of whether they were registered (total population described above, population 5)
4. All patients undergoing elective THA or TKA over the duration of the study at participating hospitals regardless of whether they were registered (a subset of population 5)

Analysing these additional populations will assess the effect of implementing the VTE prophylaxis protocol on mortality at an institutional/departmental level (the unit of randomisation), on an intention-to-treat basis.

Sub-Studies

Data from this trial will be used to form the basis of sub-studies. These will include a sub-study comparing rates of persistent wound drainage between LMWH and aspirin groups at two participating sites and a sub-study investigating rates of post-hospital discharge compliance to either study drug.

Conclusions

CRISTAL aims to provide much needed definitive evidence about the effectiveness and safety of aspirin compared to LMWH in preventing symptomatic VTE following HA or KA. This statistical analysis plan details the study’s planned analyses, including modifications to intended analyses to account for early stopping of the trial.

DECLARATIONS

Ethics Approval

Ethics approval was granted by the Sydney Local Health District (Royal Prince Alfred Zone) Human Research and Ethics Committee, which is a lead ethics committee in Australia (approval number X18-0424) prior to study commencement. Site-specific approvals for each participating hospital were granted from the following ethics committees prior to study commencement: Calvary John James Memorial Hospital Australian Capital Territory (3-2019 CRISTAL), Mid-North Coast Local Health District New South Wales (NSW – SSA/19/NCC/41), South Western Sydney Local Health District (NSW, SSA/10/LPOOL/22), Sydney Local Health District (NSW, SSA/19/RPAH/12, SSA/18/RPAH/762), Ramsay Hospital Research Foundation (NSW, HREC/18/RPAH/603), South Eastern Sydney Local Health District (NSW, 19/G/028, 18/G/338), Nepean Blue Mountains Local Health District (NSW, SSA/19/NEPAN/11), Northern Sydney Local Health District (NSW, RESP/19/027, RESP/19/028), Metro North Hospital and Health Service (Queensland, HREC/18/RPAH/603), University of South Australia (SA – 201215), Calvary Health Care Adelaide (SA, 19-CHREC-F001), Southern Adelaide Local Health Network (SA, HREC/18/RPAH/603), Bendigo Health Victoria (SSA/48255/BHCG-2019), Barwon Health (18/246), Peninsula Health Victoria (SSA/48255/PH-2019), Western Health Victoria (48255), St John of God Health Care Victoria (1540), South Metropolitan Health Western Australia (WA – Western Australia, RGS0000001358), North Metropolitan Health (WA – RGS0000001358), Sir Charles Gardiner Hospital (WA – RGS0000001358) and Launceston General Hospital Tasmania (H0017903).

Consent for publication

Not applicable.

Availability of data and materials

The datasets during and/or analysed during the current study will be made available from the corresponding author on reasonable request

Competing interests

The authors declare that they have no competing interests

Funding

The study is being funded by a four-year Medical Research Futures Fund (MRFF – Australian Federal Government) grant. The funding was not provided with any conditions and the MRFF has no authority over the design of the study or collection, management, analysis or interpretation of data or the writing of manuscripts for submission. No industry funding or other sources of funding are being used for this trial.

Authors' contributions

All authors listed have contributed to this protocol and the ICMJE guidelines were consulted for determining authorship. VS, IAH, RB, SG, SA, JMN, RdS, NP, INA, ML, DB, KC and TLK were responsible for the planning of the trial, protocol development and writing, TLK and

NP were responsible for the description of the statistical analyses used. All authors reviewed the final version of this manuscript prior to submission.

Acknowledgements

Not applicable.

REFERENCES

1. Mirkazemi C, Bereznicki LR, Peterson GM. Comparing Australian orthopaedic surgeons' reported use of thromboprophylaxis following arthroplasty in 2012 and 2017. BMC Musculoskelet Disord. 2019;20:57.
2. Adam SS, McDuffie JR, Lachiewicz PF, Ortel TL, Williams JW, Jr. Comparative effectiveness of new oral anticoagulants and standard thromboprophylaxis in patients having total hip or knee replacement: a systematic review. Ann Intern Med. 2013;159:275-84.
3. Gomez-Outes A, Terleira-Fernandez AI, Suarez-Gea ML, Vargas-Castrillon E. Dabigatran, rivaroxaban, or apixaban versus enoxaparin for thromboprophylaxis after total hip or knee replacement: systematic review, meta-analysis, and indirect treatment comparisons. BMJ. 2012;344:e3675.
4. Cao YB, Zhang JD, Shen H, Jiang YY. Rivaroxaban versus enoxaparin for thromboprophylaxis after total hip or knee arthroplasty: a meta-analysis of randomized controlled trials. Eur J Clin Pharmacol. 2010;66:1099-108.
5. Cohen A, Drost P, Marchant N, Mitchell S, Orme M, Rublee D, et al. The efficacy and safety of pharmacological prophylaxis of venous thromboembolism following elective knee or hip replacement: systematic review and network meta-analysis. Clin Appl Thromb Hemost. 2012;18:611-27.
6. Harenberg J, Marx S, Dahl OE, Marder VJ, Schulze A, Wehling M, et al. Interpretation of endpoints in a network meta-analysis of new oral anticoagulants following total hip or total knee replacement surgery. Thromb Haemost. 2012;108:903-12.
7. Neumann I, Rada G, Claro JC, Carrasco-Labra A, Thorlund K, Akl EA, et al. Oral direct Factor Xa inhibitors versus low-molecular-weight heparin to prevent venous thromboembolism in patients undergoing total hip or knee replacement: a systematic review and meta-analysis. Ann Intern Med. 2012;156:710-9.
8. Turun S, Banghua L, Yuan Y, Zhenhui L, Ying N, Jin C. A systematic review of rivaroxaban versus enoxaparin in the prevention of venous thromboembolism after hip or knee replacement. Thromb Res. 2011;127:525-34.
9. Bozic KJ, Vail TP, Pekow PS, Maselli JH, Lindenauer PK, Auerbach AD. Does aspirin have a role in venous thromboembolism prophylaxis in total knee arthroplasty patients? J Arthroplasty. 2010;25:1053-60.
10. Jameson SS, Baker PN, Charman SC, Deehan DJ, Reed MR, Gregg PJ, et al. The effect of aspirin and low-molecular-weight heparin on venous thromboembolism after knee replacement: a non-randomised comparison using National Joint Registry Data. J Bone Joint Surg Br. 2012;94:914-8.
11. Jameson SS, Charman SC, Gregg PJ, Reed MR, van der Meulen JH. The effect of aspirin and low-molecular-weight heparin on venous thromboembolism after hip replacement: a non-randomised comparison from information in the National Joint Registry. J Bone Joint Surg Br. 2011;93:1465-70.
12. Anderson DR, Dunbar M, Murnaghan J, Kahn SR, Gross P, Forsythe M, et al. Aspirin or Rivaroxaban for VTE Prophylaxis after Hip or Knee Arthroplasty. N Engl J Med. 2018;378:699-707.
13. Anderson DR, Dunbar MJ, Kahn SR. Aspirin versus low-molecular-weight heparin after total hip arthroplasty. Ann Intern Med. 2013;159:502-3.

14. Campbell MK, Piaggio G, Elbourne DR, Altman DG, Group C. Consort 2010 statement: extension to cluster randomised trials. BMJ. 2012;345:e5661.
15. Sidhu VS, Graves SE, Buchbinder R, Naylor JM, Pratt NL, de Steiger RS, et al. CRISTAL: protocol for a cluster randomised, crossover, non-inferiority trial of aspirin compared to low molecular weight heparin for venous thromboembolism prophylaxis in hip or knee arthroplasty, a registry nested study. BMJ Open. 2019;9:e031657.
16. [https://www.sealedenvelope.com]
17. Bjornara BT, Gudmundsen TE, Dahl OE. Frequency and timing of clinical venous thromboembolism after major joint surgery. J Bone Joint Surg Br. 2006;88:386-91.
18. Kulshrestha V, Kumar S. DVT prophylaxis after TKA: routine anticoagulation vs risk screening approach - a randomized study. J Arthroplasty. 2013;28:1868-73.
19. Bawa H, Weick JW, Dirschl DR, Luu HH. Trends in Deep Vein Thrombosis Prophylaxis and Deep Vein Thrombosis Rates After Total Hip and Knee Arthroplasty. J Am Acad Orthop Surg. 2018;26:698-705.
20. Giraudeau B, Ravaud P, Donner A. Sample size calculation for cluster randomized cross-over trials. Stat Med. 2008;27:5578-85.
21. Kelly T-L, Pratt N. A note on sample size calculations for cluster randomised crossover trials with a fixed number of clusters. Stat Med. 2019;38:3342-5.
22. AOA. Australian Orthopaedic Association National Joint Replacement Registry Annual Report. 2019;
23. Peto R, Pike MC, Armitage P, Breslow NE, Cox DR, Howard SV, et al. Design and analysis of randomized clinical trials requiring prolonged observation of each patient. I. Introduction and design. Br J Cancer. 1976;34:585-612.
24. Haybittle JL. Repeated assessment of results in clinical trials of cancer treatment. Br J Radiol. 1971;44:793-7.
25. Forbes AB, Akram M, Pilcher D, Cooper J, Bellomo R. Cluster randomised crossover trials with binary data and unbalanced cluster sizes: application to studies of near-universal interventions in intensive care. Clin Trials. 2015;12:34-44.
26. Turner RM, White IR, Croudace T, Group PIPS. Analysis of cluster randomized cross-over trial data: a comparison of methods. Stat Med. 2007;26:274-89.
27. White IR, Daniel R, Royston P. Avoiding bias due to perfect prediction in multiple imputation of incomplete categorical variables. Comput Stat Data Anal. 2010;54:2267-75.
28. Rodwell L, Lee KJ, Romaniuk H, Carlin JB. Comparison of methods for imputing limited-range variables: a simulation study. BMC Med Res Methodol. 2014;14:57.
29. Hughes RA, Heron J, Sterne JAC, Tilling K. Accounting for missing data in statistical analyses: multiple imputation is not always the answer. Int J Epidemiol. 2019;48:1294-304.
30. Schumi J, Wittes JT. Through the looking glass: understanding non-inferiority. Trials. 2011;12:106.

CRISTAL: Aspirin or LMWH for VTE Prophylaxis After Hip or Knee Arthroplasty

Historical Summary of Amendments for Statistical Analysis Plan

Previous Statistical Analysis (In Protocol): 29 October 2020

Updated in Initial Statistical Analysis Plan: 14 May 2021

AMENDMENT 1 – Changes from Protocol to Initial Statistical Analysis Plan

8. **ITEM:** STUDY OVERVIEW – Outcome Variables. Change in time point for secondary outcome of mortality as written in protocol
   CHANGE: Mortality to be measured at 90 days only, not at 90 days and 6 months
   RATIONALE: Due to the lack of sensitivity in measuring VTE-related mortality at 6 months and due to the lag in data availability for mortality, we changed the analysis to measure mortality at 90 days only
9. **ITEM:** MAIN ANALYSES
   CHANGE: Inclusion of composite analysis method to account for incomplete crossover
   RATIONALE: After the interim analyses demonstrated that the stopping rule had been met, a composite analysis method was required given that a number of sites had not completed crossover
10. **ITEM:** MAIN ANALYSES – Subgroup Analyses.
    CHANGE: Addition of patients with a prior history of VTE or not and of patients on long-term single antiplatelet therapy as subgroup analyses (not previously in study protocol)
    RATIONALE: Given this data was collected preoperatively, the senior author (IAH), corresponding author (VS), lead statistician (TLK) and senior statistician (NP) decided to add these subgroup analyses
11. **ITEM:** MAIN ANALYSES – Subgroup Analyses.
    CHANGE: Clarification of the method used for subgroup analyses (using summaries by cluster and subgroup and an interaction term)
    RATIONALE: Methods used for subgroup analyses added to initial version of statistical analysis plan
12. **ITEM:** MAIN ANALYSES – Sensitivity Analyses.
    CHANGE: Addition of sensitivity analyses to determine: (a) the effect of high-volume arthroplasty sites; (b) sites with high and low overall registration rates; (c) sites that required multiple compliance audits and method used for sensitivity analyses provided (same as subgroup analyses)

RATIONALE: To determine if the results for the primary outcome were consistent amongst sites despite variations in the above parameters. Addition of methods used for sensitivity analyses

13. **ITEM:** MAIN ANALYSES – Order of Analyses
    CHANGE: Order of planned analyses changed to include interim analyses and specification of the Haybittle-Peto threshold
    RATIONALE: The initial statistical analyses in the protocol did not include the interim analysis and this was included in the initial version of the statistical analysis plan

Previous Statistical Analysis Plan (Initial): 14 May 2021

Updated in Statistical Analysis Plan (Final): 20 July 2021

AMENDMENT 2 – Changes from Initial Statistical Analysis Plan to Final Statistical Analysis Plan

1. **ITEM:** <u>STATISTICAL ANALYSIS PLAN – Methods used for Interim Analyses</u>
   CHANGE: Addition of using harmonic mean weighting as a method to account for unequal cluster sizes
   RATIONALE: Given early trial cessation and unequal cluster sizes, further information provided on how unequal cluster sizes were analysed
2. **ITEM:** <u>STATISTICAL ANALYSIS PLAN – Methods for handling missing data</u>
   CHANGE: Addition of methods used for multiple imputation, "The imputation model will use auxiliary variables gathered from routine AOANJRR data (including age, sex, baseline health, pain and function, diagnosis and surgical factors), as well as cluster and period effects. One hundred datasets will be imputed at the patient level, then each dataset will be analysed using the main analysis method with cluster summaries and combined using Rubin's rules."
   RATIONALE: More in-depth description provided to account for clustering
3. **ITEM:** <u>STATISTICAL ANALYSIS PLAN – Methods for handling missing data</u>
   CHANGE: Addition of the use of inverse probability weighting as a sensitivity analysis for the primary outcome to handle missing data
   RATIONALE: An additional method (to that of multiple imputation) to account for missing data
4. **ITEM:** <u>STATISTICAL ANALYSIS PLAN – Trial and baseline characteristics</u>
   CHANGE: Addition of demographic information to Table 1 for participating hospitals (clusters) – number of joint replacements performed in year preceding trial, insurance status
   RATIONALE: Prior to this, there was no demographic description of participating clusters, only numbers registered by each hospital
5. **ITEM:** <u>MAIN ANALYSES:</u> Analyses of primary and secondary outcomes
   CHANGE: Description of composite methods used (cluster weighted estimates) for primary and secondary outcomes
   RATIONALE: To explain methods used to mitigate bias and any possible loss of precision from sites that had incomplete crossover or did not crossover
