# Supplementary material for: Effect of Aspirin vs Enoxaparin on 90-Day Mortality in Patients Undergoing Hip or Knee Arthroplasty: A Secondary Analysis of the CRISTAL Cluster Randomized Trial
Source: JAMA Netw Open. 2023 Jun 9;6(6):e2317838. doi: 10.1001/jamanetworkopen.2023.17838 (PMC10257098; doi:10.1001/jamanetworkopen.2023.17838)
Supplement: Supplement 2. — Data Sharing Statement [file jamanetwopen-e2317838-s002.pdf]

Data Sharing Statement

The CRISTAL Study Group. Effect of Aspirin vs Enoxaparin on 90-Day Mortality in Patients Undergoing Hip or Knee Arthroplasty. *JAMA Netw Open*. Published June 09, 2023. doi:10.1001/jamanetworkopen.2023.17838

Data
Data available: Yes
Data types: Deidentified participant data
How to access data: De-identified data will be made available upon reasonable request from the senior author, Prof Ian Harris, via email: ianharris@unsw.edu.au.
When available: With publication

Supporting Documents
Document types: None

Additional Information
Who can access the data: Data will be made available to those whose proposed use of the data has been approved by the governing human research ethics committee.
Types of analyses: Data must be used for non-commercial, research purposes only.
Mechanisms of data availability: Data will be made available with investigator support subject to approval by the governing human research ethics committee.
Any additional restrictions: Any future analyses using study data must include at least one senior investigator from the original study to provide context and ensure correct use and appropriate interpretation of the data.
